# O_2_‑Coupled Copper–Cysteine Redox Chemistry Drives Oxidative Modifications and Higher-Order Assembly of Monomeric Transthyretin

**DOI:** 10.1021/acscentsci.6c00619

**Published:** 2026-10-05

**Authors:** Yelim Yi, Wooyeol Ryu, Bokyung Kim, Srinivasan Muniyappan, Jin Hae Kim, Kiyoung Park, Mi Hee Lim

**Affiliations:** † Department of Chemistry, Korea Advanced Institute of Science and Technology (KAIST), Daejeon 34141, Republic of Korea; ‡ Department of New Biology, Daegu Gyeongbuk Institute of Science and Technology (DGIST), Daegu 42988, Republic of Korea

## Abstract

Copper–cysteine (Cu–Cys) coordination is pervasive in biology, yet how Cu–thiol redox chemistry engages O_2_ and translates into protein-level consequences remains incompletely understood. Here, we establish a generalizable protein-based platform that directly captures O_2_-coupled Cu­(II)–Cys redox processes and delineates their structural and functional outcomes. Integrated spectroscopic, mass spectrometric, and computational investigations reveal that Cu­(II) coordination to a protein Cys residue initiates redox cycling that generates thiyl radicals and/or disulfide radical anions. This chemically defined process drives disulfide bond formation together with O_2_-dependent oxidative transformations, producing site-specific Cys modifications, dityrosine cross-links, and higher-order protein assemblies. Under these conditions, these structural changes abolish the protein’s anti-amyloidogenic activity and result in apoptotic responses. By directly linking Cu–thiol redox chemistry to protein oxidation and aggregation, this work establishes a fundamental chemical principle by which redox-active metal ions govern protein fate under aerobic conditions. More broadly, these findings provide a mechanistic framework for understanding how metal-mediated oxidative chemistry contributes to protein misfolding and aggregation in human diseases.

## Graphical abstract

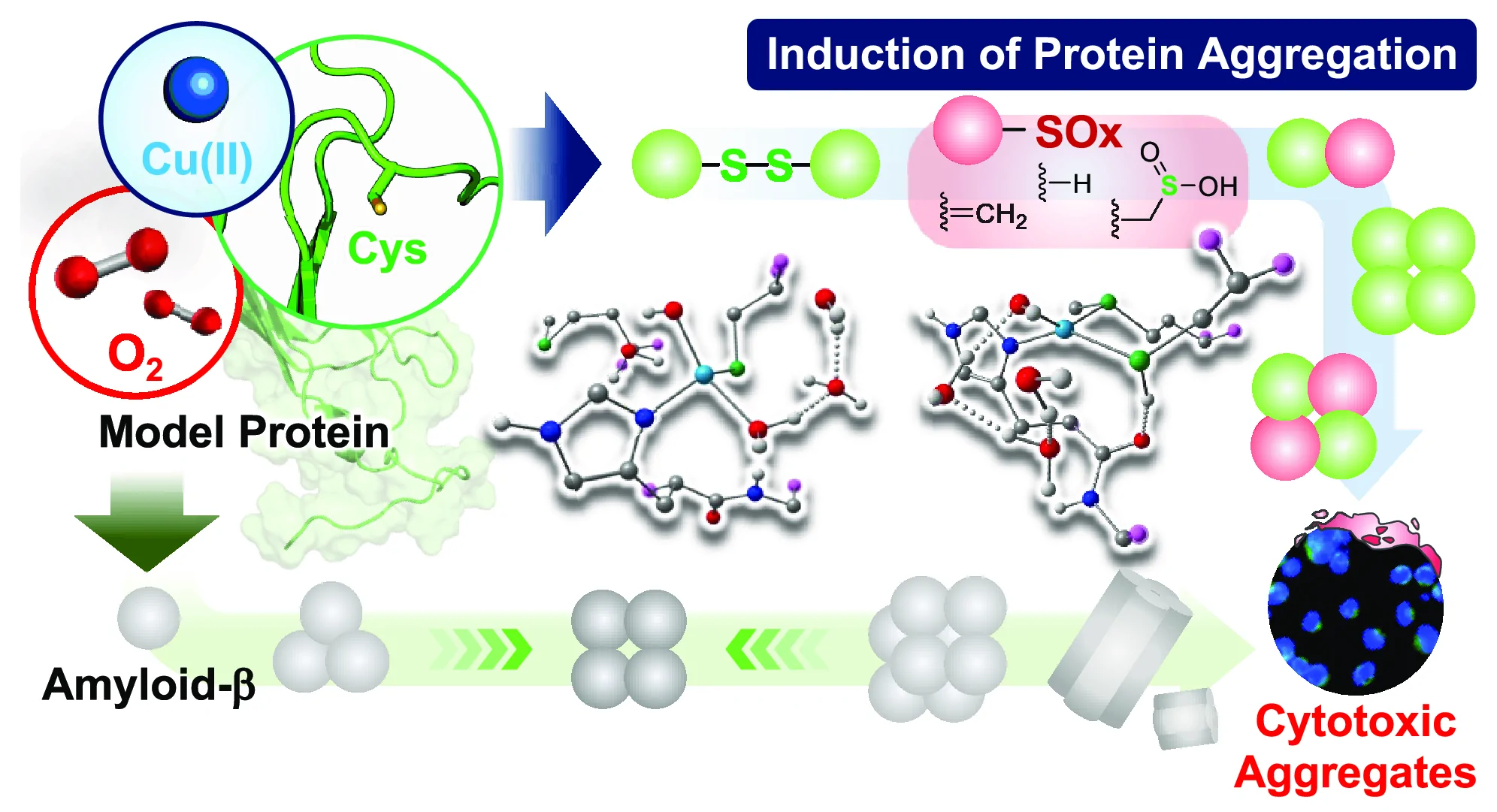

## Introduction

Copper–cysteine (Cu–Cys) coordination is ubiquitous in biological systems, particularly in proteins that mediate electron transfer and dioxygen (O_2_) activation.^1,2^ The redox versatility of Cu–thiol interactions underlies essential catalytic and regulatory processes; however, under aerobic conditions, this same reactivity can also promote oxidative protein damage. Despite extensive studies on copper redox chemistry, the mechanistic links between O_2_-coupled Cu–Cys redox processes and defined structural and functional consequences at the protein level remain incompletely understood. Addressing this gap is critical for understanding oxidative stress-driven protein dysfunction, especially in neurodegenerative disorders.^1,2^

Previous efforts to interrogate Cu–Cys redox chemistry have largely relied on synthetic model complexes.^3–10^ The intrinsic redox lability of Cu­(II)–thiolate species, however, often necessitates substitution with more stable ligands, such as thioethers, which fail to reproduce the O_2_ reactivity characteristic of native Cu–Cys coordination (Figure 1a).^4–16^ Moreover, synthetic systems inherently lack the structural, dynamic, and interactional complexity of proteins, where local environments and conformational changes critically influence redox outcomes.^16^ As a result, the mechanisms by which Cu–Cys redox chemistry operates within protein environments, and how these chemical events are translated into structural and functional changes at the protein-level, remain poorly defined.

**Figure 1:**
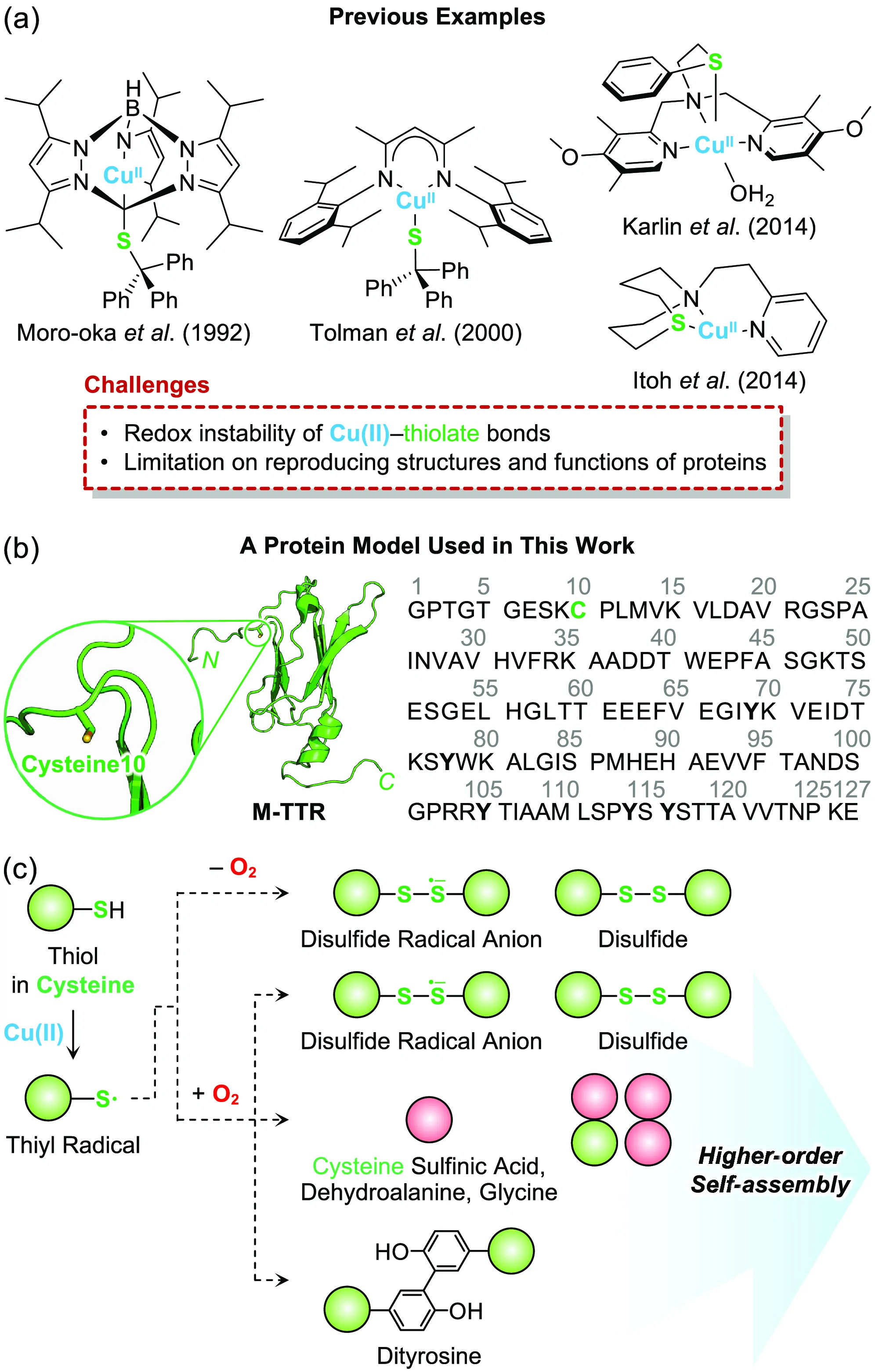
Overview of a protein-based strategy to elucidate Cu­(II)–Cys redox chemistry in the presence of O_2_. (a) Previous examples of Cu­(II)–thiolate or thioether coordination in synthetic models. (b) Structure and sequence of M-TTR (PDB 2NBO^17^) as a protein model used in this work. (c) Schematic representation of O_2_-dependent Cu­(II)-induced chemical modifications at a Cys residue with conformational changes discovered in this study.

In this study, we establish a generalizable protein-based strategy to directly capture O_2_-coupled Cu­(II)–Cys redox chemistry and identify its structural and functional consequences. To overcome the limitations of model systems, we employ a metal-free protein scaffold containing a single, solvent-exposed Cys residue, enabling site-specific formation of a Cu­(II)–Cys complex *in situ*. Using monomeric transthyretin (M-TTR) as a tractable and biologically relevant platform (Figure 1b), we integrate spectroscopic, mass spectrometric, and computational approaches to define Cu­(II) coordination and its redox evolution within a controlled protein environment.

Within this framework, we delineate the progression from Cu­(II)–Cys to the formation of thiyl radical and/or disulfide radical anion as a key initiating event that drives disulfide bond formation and O_2_-dependent oxidative transformations (Figure 1c). These chemically defined processes generate site-specific Cys modifications and dityrosine cross-links that lead to higher-order protein assembly. These aggregates are not able to modulate the aggregation and toxicity of another amyloidogenic amyloid-β (Aβ) peptide closely linked to Alzheimer’s disease (AD).^18–20^ By directly linking Cu–thiol redox chemistry to protein oxidation and aggregation, this work establishes a molecular principle by which redox-active metal ions determine protein oxidation, assembly, and activity under aerobic conditions. More broadly, these findings provide a mechanistic framework for understanding how metal-mediated oxidative chemistry contributes to protein misfolding and aggregation processes associated with neurodegenerative disorders.

## Results and Discussion

### Proof-of-Concept Demonstration Using a Protein Model

To directly probe the chemical and biological consequences of Cu­(II)–Cys coordination, we established a structurally defined protein model that enables the controlled interrogation of Cu–thiol redox chemistry. We reasoned that introducing Cu­(II) into a protein containing a single accessible Cys residue would allow the precise characterization of Cu–Cys redox processes and O_2_-dependent reactions, thereby revealing their specific chemical and biological outcomes at the protein level. For this purpose, we employed M-TTR, a tractable protein scaffold containing a single solvent-exposed Cys residue (Cys10),^21,22^ as depicted in Figure 1b. The presence of a solitary Cys eliminates complications arising from multiple thiol sites,^21,22^ enabling the unambiguous analysis of Cu­(II) coordination chemistry. Upon Cu­(II) binding to Cys10, the metal center remains solvent-accessible and is therefore well positioned to participate in redox reactions with O_2_. This simplified yet biologically relevant system provides a controlled platform to examine Cu­(II)–Cys coordination and its impact on protein structure and function.

Beyond its structural simplicity, the M-TTR scaffold also offers a biologically meaningful framework for investigating the conformational and functional consequences of Cu­(II) coordination. Native TTR exists as a stable tetramer, but the dissociation into monomeric units (M-TTR) represents a key intermediate in the formation of pathological amyloid fibrils associated with various human diseases.^18,23–25^ Accordingly, M-TTR provides a physiologically relevant model for examining how chemical perturbations induced by Cu­(II) coordination may influence protein misfolding and aggregation.

For M-TTR, we utilized the engineered TTR monomer, TTR­(Phe87Met/Leu110Met), which remains stably monomeric under neutral aqueous conditions yet undergoes aggregation in response to defined environmental triggers, such as acidic pH.^17,26^ This controlled aggregation behavior makes M-TTR an attractive platform for probing whether Cu­(II) coordination to Cys10 can induce structural perturbations that initiate or accelerate aggregation processes. Furthermore, M-TTR exhibits intrinsic anti-amyloidogenic activity toward amyloid-β (Aβ), a peptide closely linked to AD.^27–33^ This property further provides a functional context to assess how Cu­(II)–Cys coordination may alter protein activity and influence amyloid-related processes.

### Cu­(II) Coordination to the Protein Model

To characterize Cu­(II) binding and confirm the involvement of the Cys10 residue, we employed integrated spectroscopic, mass spectrometric, and computational analyses. Upon addition of CuCl_2_ (1 equiv) to M-TTR, a pronounced electronic absorption (Abs) band appeared at 380 nm (Figure 2a), accompanied by weaker, broad features spanning approximately 500–1,000 nm. These spectral signatures were absent in the control spectra of M-TTR alone or free CuCl_2_ (Figure S1a), indicating that the observed Abs signals arise specifically from Cu­(II) coordination to the protein. Over the course of 24 h, the intense band at 380 nm gradually decreased, whereas the lower-energy Abs features remained largely unchanged, suggesting structural or redox evolution within the Cu­(II) complex over time.

**Figure 2:**
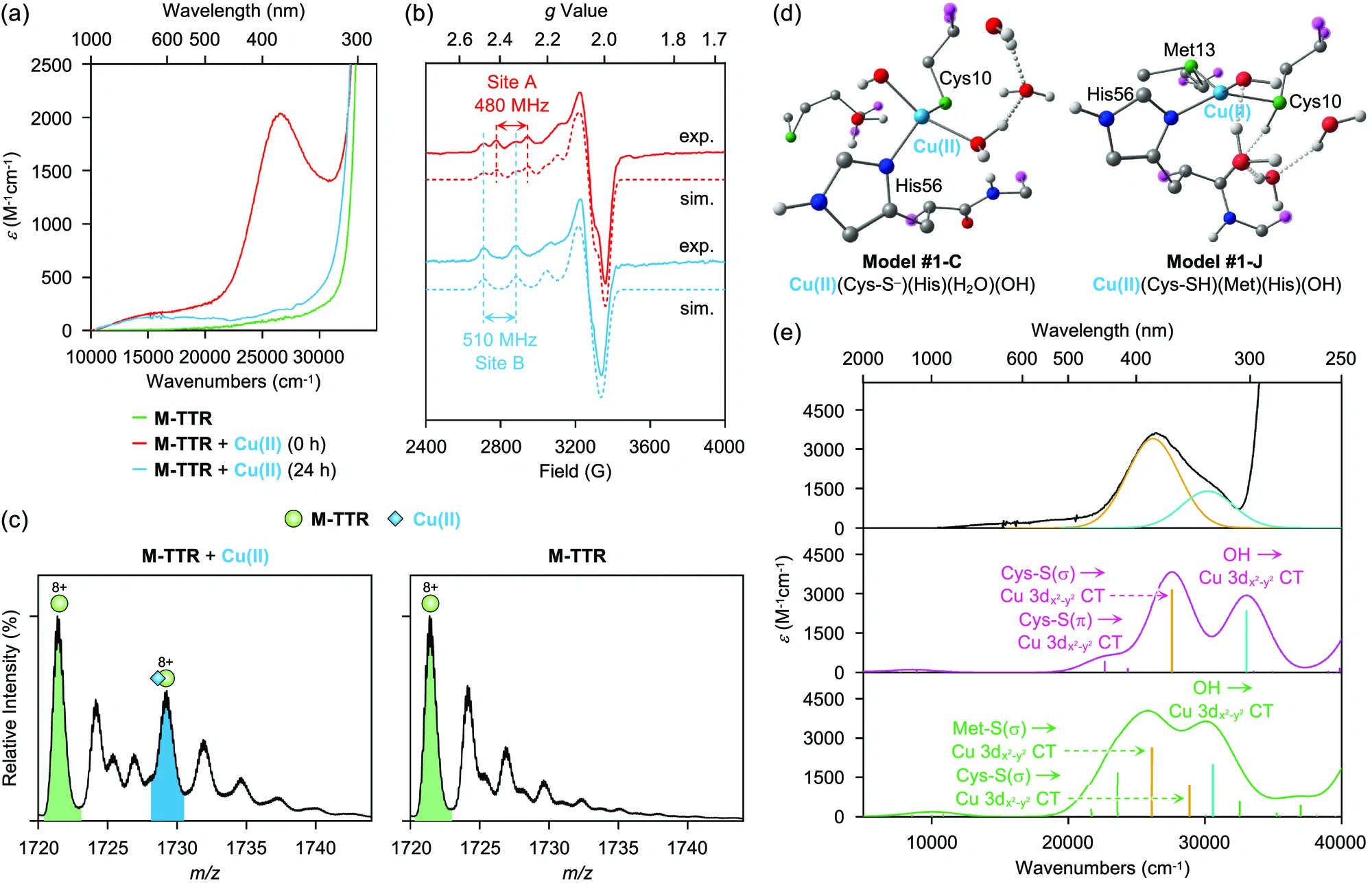
Spectroscopic and spectrometric studies of Cu­(II)–M-TTR and spectroscopically validated DFT models for site A. (a and b) Abs and EPR spectra collected at 37 °C and 100 K, respectively, of the M-TTR samples in the absence (green) and presence of CuCl_2_ (1 equiv) obtained at 0 (red) and 24 h (cyan) after mixing. The latter EPR spectrum could be simulated with a single site (referred to as site B), while the former exhibited two distinct Cu­(II) centers, 60% attributed to site B and the remaining 40% designated as site A. The simulated spectra (dashed) were obtained with the EPR parameters: (for site A) *g*_*x*_, *g*_*y*_, *g*_*z*_ = 2.017, 2.075, 2.225 and *A*_*x*_, *A*_*y*_, *A*_*z*_ = 0, 50, 480 MHz; (for site B) *g*_*x*_, *g*_*y*_, *g*_*z*_ = 2.030, 2.065, 2.270 and *A*_*x*_, *A*_*y*_, *A*_*z*_ = 0, 0, 510 MHz. Conditions: [M-TTR] = 100 μM; [CuCl_2_] = 100 μM; 20 mM HEPES, pH 7.4, 150 mM NaCl. (c) Mass spectra of M-TTR incubated with or without Cu­(II). Conditions: [M-TTR] = 100 μM; [CuCl_2_] = 100 μM; 20 mM ammonium acetate, pH 7.4; 37 °C; 3 h incubation; quiescent conditions. The samples were diluted 10-fold before injection into the mass spectrometer. The measurements were conducted in triplicate. (d) DFT models that can reproduce the Abs and EPR spectral features of site A. Cyan, green, gray, blue, red, and white balls indicate copper, sulfur, carbon, nitrogen, oxygen, and hydrogen atoms, respectively. For clarity, hydrogen atoms attached to carbon atoms are omitted, while atoms constrained during geometry optimization are highlighted in pink. (e) Experimental Abs spectrum of site A (black) and time-dependent (TD) DFT-simulated Abs spectra of models #1-C (pink) and #1-J (green). Using the nitrosocyanin-based calibration, the DFT-calculated spectra were down-shifted by 2,600 cm^–1^. Deconvoluted Gaussian bands at 330 and 380 nm and their corresponding TDDFT-calculated states are presented in cyan and orange, respectively.

Titration experiments were performed by incrementally adding Cu­(II) to M-TTR while monitoring the Abs intensity at 380 nm (Figure S2a). The observed spectral changes are consistent with Cu­(II) binding to M-TTR with an affinity in the submicromolar range under our experimental conditions (Figure S2b). It should be noted that a thermodynamic *K*_d_ value could not be reliably determined from these data because the Cu­(II)–Cys complex undergoes redox-dependent changes over time and protein aggregation occurs at higher Cu­(II)-to-M-TTR ratios (*vide infra*), precluding rigorous equilibrium binding analysis. The Cu­(II)-to-M-TTR stoichiometry of the 380 nm-absorbing species was further examined by Job’s method of continuous variation, as shown in Figure S2c. M-TTR and Cu­(II) were mixed at varying molar ratios while maintaining a constant total concentration, and the Abs at 380 nm was monitored. As depicted in Figure S2d, the resulting Job plot exhibited a maximum at a Cu­(II) mole fraction of *ca*. 0.5, consistent with an approximately 1:1 Cu­(II)-to-M-TTR stoichiometry for the 380 nm-absorbing species (*vide infra*) present during the Cu­(II)–Cys redox process. This stoichiometry describes the composition of the 380 nm-absorbing species and should be distinguished from the relative populations of the EPR-detectable Cu­(II) species assigned to sites A and B (*vide infra*).

Electron paramagnetic resonance (EPR) spectroscopy further revealed two distinct Cu­(II)-binding sites within M-TTR (Figure 2b). Immediately after mixing Cu­(II) with the protein, rapidly freezing in liquid nitrogen produced two sets of hyperfine features: one with *A*_||_ ∼ 480 MHz at *g*_||_ ∼ 2.23 (site A) and the other with *A*_||_ ∼ 510 MHz at *g*_||_ ∼ 2.27 (site B). After incubation for 24 h, the EPR spectrum converged to a single Cu­(II) species corresponding to site B. Using the pure spectrum from site B, the initial spectrum (0 h) was deconvoluted to estimate a composition of *ca*. 40% site A and 60% site B (Figure S3).

Guided by this ratio, the Abs spectra in Figure 2a were similarly resolved (Figure S1b-d). The intense Abs at 380 nm primarily originates from site A, whereas the weaker bands at lower energies arise from contributions of both sites. The low-intensity bands (ε = *ca*. 150–240 M^–1^cm^–1^) are consistent with Cu­(II) *d-d* transitions. In contrast, the strong band at 380 nm is assigned to a charge transfer (CT) transition. Analysis of the deconvoluted Abs spectrum of site A revealed two major transitions at approximately 330 and 380 nm, suggesting coordination by strong σ-donor ligands. This interpretation is supported by the smaller EPR *g*_||_ value observed for site A, which indicates a larger energy gap between the Cu 3d_*xy*_-based nonbonding molecular orbital (MO) and 3d_x^2^‑y^2^_-based σ*-antibonding MO at site A relative to site B. These spectroscopic characteristics closely resemble those of type II red copper proteins, which exhibit intense S-to-Cu­(II) CT transitions near 380–400 nm and typical Cu­(II) EPR signals (*A*_||_ > *ca*. 400 MHz) (Table S1). Formation of the Cu­(II)–M-TTR complex was further confirmed by electrospray ionization–mass spectrometry (ESI–MS). As shown in Figure 2c, the addition of CuCl_2_ (1 equiv) generated a distinct peak at *ca*. 1,729 *m*/*z*, corresponding to the [M-TTR + Cu­(II) + 6H]^8+^ species, indicative of the complexation between Cu­(II) and M-TTR. This signal was absent in the spectrum of M-TTR alone.

To identify plausible Cu­(II)-binding sites, density functional theory (DFT) calculations were performed following the spectroscopic characteristics described above, with the reliability of the DFT methods independently verified (Figures S4–S6).^34,35^ These Cu­(II)-binding sites were modeled based on the crystal structure of Zn­(II)–TTR (PDB 3GRG^36^). This structure contains three potential metal-binding sites: (i) site 1 (His56, Cys10, and two water molecules); (ii) site 2 (His88, His90, Glu92, and a water molecule); (iii) site 3 (His31, Glu72, Asp74, and a water molecule). In this study, Met13 was also considered for site 1 because of its proximity to the metal center. A total of 19 structural models were constructed by varying ligand protonation states and coordination environments while constraining backbone atoms adjacent to the α-carbons (Cαs) (Figures S7–S12).

Models derived from sites 2 and 3, which lack Cys coordination, failed to reproduce the Abs features at 330 and 380 nm associated with site A, although they may correspond to the spectroscopic properties of site B (Figures S13–S15 and Table S2). In contrast, 15 models derived from site 1 successfully reproduced the experimental spectra when Cys coordination was included. Two models in particular, Cys/His/H_2_O/OH^–^ (model #1-C) and Cys/Met/His/OH^–^ (model #1-J), accurately reproduced the characteristic Abs features (Figure 2d-e). These calculations indicate that the 380 nm band originates from an S-to-Cu­(II) CT transition, while the 330 nm feature arises from a hydroxide-to-Cu­(II) CT transition. Collectively, these spectroscopic, mass spectrometric, and computational results support direct coordination of Cu­(II) to the Cys10 residue of M-TTR, validating this protein as an effective model system for investigating Cu­(II)–Cys redox chemistry. In addition, sites A and B represent distinct Cu­(II)-binding environments with likely different mechanistic roles. Site A is directly associated with Cu­(II)–Cys redox chemistry and the resulting protein oxidation and aggregation (*vide infra*), whereas the mechanistic role of the more persistent site B remains to be elucidated.

### Disulfide Bond Formation

Disulfide bond formation is a hallmark outcome of thiol oxidation as a key consequence of Cu­(II)–Cys redox chemistry.^34,35,37–39^ To determine whether Cu­(II) coordination to M-TTR induces disulfide bond formation, Cu­(II)-treated protein samples were incubated in the absence or presence of 1,4-dithiothreitol (DTT), a well-established disulfide-reducing agent (Figure 3a),^40,41^ and analyzed by sodium dodecyl sulfate–polyacrylamide gel electrophoresis (SDS–PAGE) followed by Western blotting using an anti-TTR antibody (Figure S16). As shown in Figure 3b, the treatment of M-TTR with 1 equiv of Cu­(II) resulted in the appearance of bands corresponding to dimeric species (*ca*. 28 kDa; 25–35 kDa). The intensity of this band decreased upon DTT addition. To exclude the possibility that DTT interferes with Cu-mediated redox chemistry^42^ rather than reducing preformed disulfide bonds, Cu­(II) was removed from the reaction mixture by Chelex treatment prior to DTT addition. Although Chelex treatment itself reduced the overall intensities of the dimer and higher-order bands, subsequent DTT treatment eliminated the dimer band, confirming that the dimers are linked by disulfide bonds. In addition to dimerization, Cu­(II) treatment promoted the formation of higher-order aggregates, which became particularly pronounced with 2 equiv of Cu­(II). Notably, even after Cu­(II) removal by Chelex treatment, DTT substantially diminished not only the higher-order aggregate bands but also the remaining dimer band, indicating that these larger assemblies are likewise stabilized predominantly by disulfide cross-links. It should be noted that dimeric M-TTR was detected even in the absence of Cu­(II), although at a lower band intensity than that observed for the Cu­(II)-treated M-TTR samples without Chelex or DTT treatment. The band was abolished by DTT treatment. This background dimerization likely arises from the intrinsic instability of M-TTR at 37 °C^40,43^ or trace amounts of residual Cu remaining from protein purification (Figure S17).

**Figure 3:**
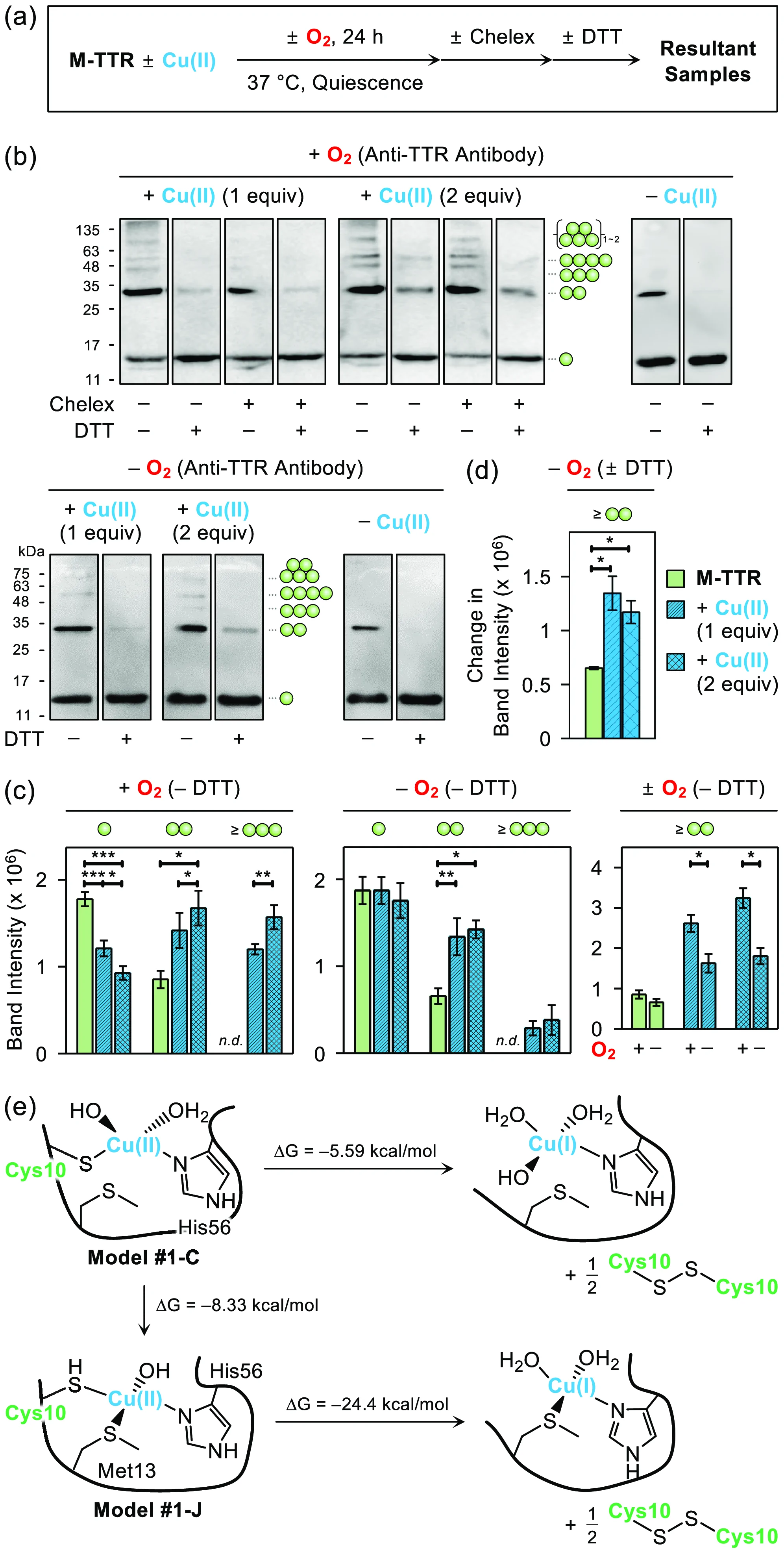
Cu­(II)-driven disulfide bond formation and aggregate formation. (a) Scheme of the experiments. (b) Size distribution of the resultant M-TTR species with or without Cu­(II) probed by SDS–PAGE with Western blotting using an anti-TTR antibody. Conditions: [M-TTR] = 25 μM; [CuCl_2_] = 25 μM and 50 μM; 20 mM HEPES, pH 7.4, 150 mM NaCl; 37 °C; 24 h incubation; quiescent conditions. (c,d) Intensity of the bands corresponding to M-TTR species generated dependent on O_2_ and DTT. These are quantified by the Image Lab software. *n*.*d*., not determined. Data are presented as mean ± s.e.m. of three independent experiments (*n* = 3). All statistical significance was determined by a two-sided unpaired Student’s *t*-test. *P < 0.05, **P < 0.01, and ***P < 0.001. (e) Thermodynamics involved in Cu­(II)-induced disulfide bond formation in M-TTR.

To evaluate the involvement of O_2_ in Cu­(II)-mediated disulfide bond formation, parallel experiments were carried out under anaerobic conditions. In the presence of Cu­(II) but the absence of O_2_, the bands corresponding to dimers and aggregates up to the pentamers (below 75 kDa) were still observed (Figure 3c). The intensity of higher-order aggregates, however, was noticeably lower than that observed under aerobic conditions. As in the aerobic samples, these bands were largely eliminated upon treatment with DTT, indicating that disulfide bond formation occurs even in the absence of O_2_. Samples lacking Cu­(II) displayed only monomer and dimer bands under anaerobic conditions. The quantitative analysis of band intensities under anaerobic conditions (Figure 3d) further confirmed that Cu­(II) significantly promotes disulfide bond formation, as evidenced by the notable reduction in aggregate levels following DTT treatment. Furthermore, the quantitative analysis of dimer and higher-order aggregate formation under aerobic and anaerobic conditions revealed a pronounced O_2_ dependence of Cu­(II)-induced M-TTR aggregation. As shown in Figure 3c, aggregate abundance under aerobic conditions was *ca*. 38% and 44% higher than under anaerobic conditions in the presence of 1 and 2 equiv of Cu­(II), respectively, whereas no significant difference was observed in the absence of Cu­(II). Together, these results demonstrate that O_2_ acts in concert with Cu­(II) to enhance the formation of dimeric and higher-order M-TTR assemblies.

The spectroscopic and computational studies described above further support this redox process. In particular, the disappearance of the site A signal after 24 h in the EPR analysis (Figure 2b) correlates with the oxidation of the Cys residues and subsequent disulfide bond generation, as presented in Figure 3e. Among the computational models, model #1-J was identified as the most energetically favorable structure, in which a nearby Met residue stabilizes the resulting Cu­(I) species more effectively than a water ligand in model #1-C. These findings suggest that Cu­(II) coordination to the Cys residue initiates Cys oxidation and disulfide bond formation through a Cu-driven redox process that can proceed even in the absence of O_2_.

### Oxidative Modifications

To directly examine whether Cu­(II) is reduced during its reaction with M-TTR, Cu­(I) formation was monitored using the BCA assay (BCA = 2,2′-biquinoline-4,4′-dicarboxylic acid). As shown in Figure S18, the characteristic Abs band of the Cu­(I)­(BCA)_2_ complex at 562 nm increased progressively in the samples containing M-TTR and Cu­(II), whereas negligible signal was detected for M-TTR in the absence of added Cu­(II). The estimated concentration of Cu­(I)­(BCA)_2_ increased from *ca*. 6 μM immediately after BCA addition to *ca*. 21 μM after 2 h of incubation, indicating the time-dependent formation of Cu­(I)­(BCA)_2_. These results are consistent with the reduction of Cu­(II) to Cu­(I) occurring in the presence of M-TTR. It should be noted, however, that BCA can facilitate the reduction of Cu­(II) to Cu­(I);^44,45^ therefore, the observed Cu­(I)­(BCA)_2_ formation cannot be attributed solely to Cu­(II) reduction by M-TTR.

Such oxidation is expected to proceed through transient protein-centered radical intermediates, including thiyl radical and/or disulfide radical anion.^46–51^ To probe their formation, we employed TEMPO [(2,2,6,6-tetramethylpiperidin-1-yl)­oxyl], a stable nitroxide radical whose EPR signal decreases upon reaction with radical intermediates.^52,53^ As illustrated in Figure 4a, TEMPO was mixed with M-TTR and Cu­(II) under anaerobic conditions, and the resulting sample was immediately frozen without prolonged incubation prior to EPR analysis. Under these conditions, the EPR spin number decreased by 41 ± 3% relative to TEMPO alone, indicating a loss of EPR-detectable TEMPO radicals during the reaction. These results support the generation of transient radical intermediates upon Cu­(II)-mediated oxidation of M-TTR under anaerobic conditions. Notably, the extent of TEMPO signal loss closely matches the relative population of site A (Figure 2b), suggesting that the Cys-containing Cu­(II)-binding site is the primary locus for the generation of protein-centered radicals, including thiyl radicals and/or disulfide radical anions. It should be noted that the EPR signal of TEMPO was unaffected by either Cu­(II) or Cu­(I) under our experimental conditions (Figure S19), confirming that the observed decrease of TEMPO signal arises from its reaction with transient radical intermediates rather than direct interactions with Cu­(I/II).

**Figure 4:**
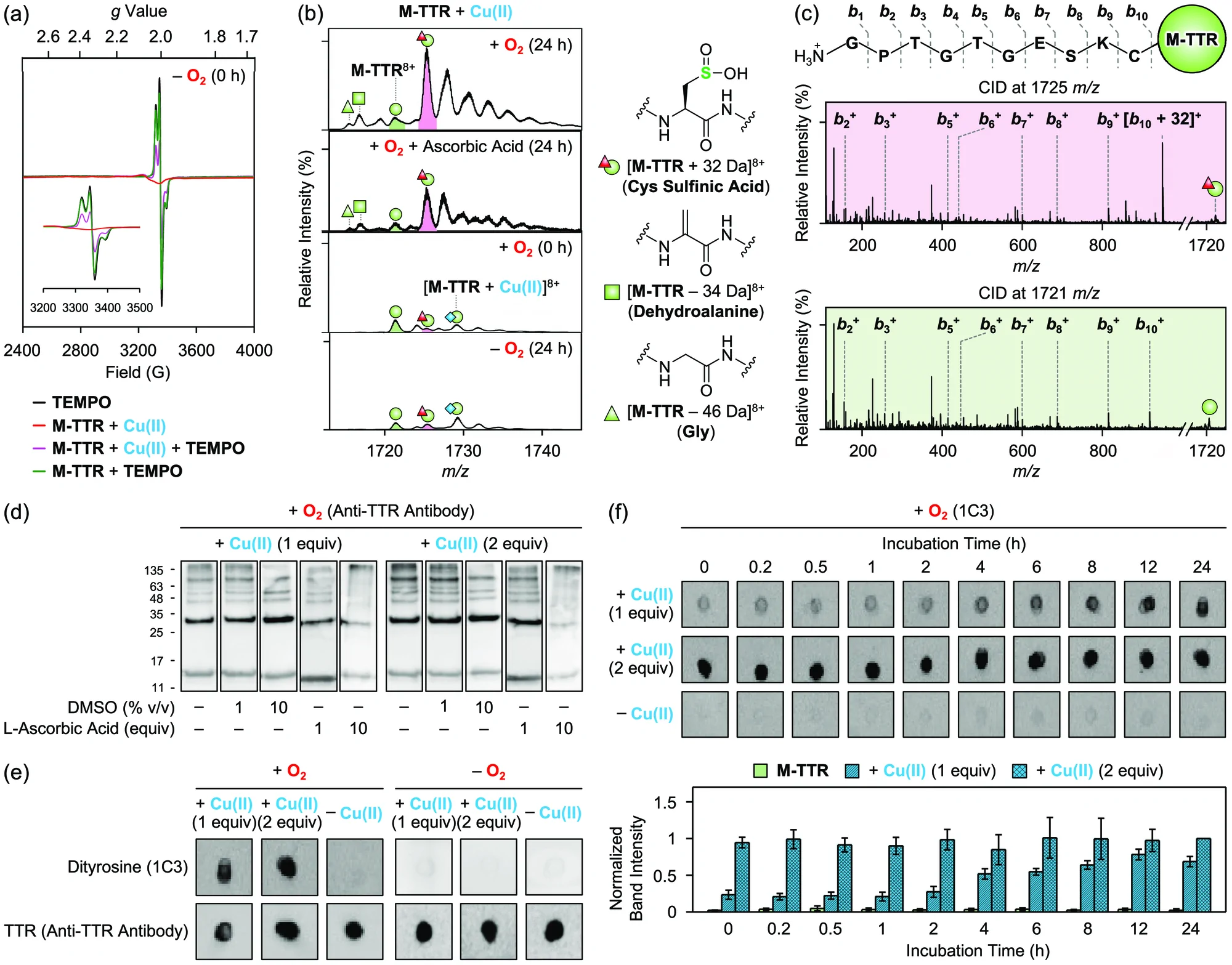
O_2_-coupled, Cu­(II)-induced modifications of M-TTR. (a) EPR spectra of TEMPO mixed with M-TTR in the presence or absence of Cu­(II) under anaerobic conditions and immediately frozen without prolonged incubation. Conditions: [M-TTR] = 100 μM; [CuCl_2_] = 100 μM; [TEMPO] = 100 μM; 20 mM HEPES, pH 7.4, 150 mM NaCl. (b) Mass spectra of M-TTR incubated with Cu­(II) under aerobic and anaerobic conditions and chemical structures of the products obtained by Cu­(II)-induced chemical modifications under aerobic conditions. The peaks of native and doubly oxidized M-TTR are highlighted in green and pink, respectively. Conditions: [M-TTR] = 100 μM; [CuCl_2_] = 100 μM; [l-ascorbic acid] = 100 μM; 20 mM ammonium acetate, pH 7.4; 37 °C; 24 h incubation; quiescent conditions. The samples were diluted 10-fold before injection into the mass spectrometer. The measurements were conducted in triplicate. (c) ESI–MS^2^ analysis of the doubly oxidized (1,725 *m*/*z*; pink) and native (1,721 *m*/*z*; green) M-TTR in the presence of Cu­(II) and O_2_. (d) Size distribution of Cu­(II)-treated M-TTR incubated with or without DMSO or l-ascorbic acid monitored by SDS–PAGE with Western blotting using an anti-TTR antibody. Conditions: [M-TTR] = 25 μM; [CuCl_2_] = 25 μM and 50 μM; 1% and 10% v/v DMSO; [l-ascorbic acid] = 25 μM and 250 μM; 20 mM HEPES, pH 7.4, 150 mM NaCl; 37 °C; 24 h incubation; quiescent conditions. (e) Dot blot analysis of Cu­(II)-treated and untreated protein samples using anti-dityrosine (1C3) and anti-TTR antibodies. The measurements were conducted in triplicate. Conditions: [M-TTR] = 25 μM; [CuCl_2_] = 25 μM and 50 μM; 20 mM HEPES, pH 7.4, 150 mM NaCl; 37 °C; 24 h incubation; quiescent conditions. (f) Time-dependent dityrosine formation in Cu­(II)-treated protein samples probed by the dot blot assay using an anti-dityrosine (1C3) antibody. The measurements were conducted in triplicate. Conditions: [M-TTR] = 25 μM; [CuCl_2_] = 25 μM and 50 μM; 20 mM HEPES, pH 7.4, 150 mM NaCl; 37 °C; quiescent conditions.

To determine whether the Cu­(II)-mediated Cys redox chemistry observed under anaerobic conditions follows the same Cu­(II)-binding behavior previously identified under aerobic conditions (Figure 2b), EPR spectroscopy was performed under anaerobic conditions, as shown in Figure S20. Immediately after mixing Cu­(II) with M-TTR, the EPR spectrum exhibited the same hyperfine features corresponding to the two distinct Cu­(II)-binding sites (sites A and B) observed under aerobic conditions. After 24 h of anaerobic incubation, the spectrum converged to a single Cu­(II) species corresponding to site B. Interestingly, when this sample was subsequently exposed to O_2_ for 20 min and frozen again for the EPR analysis, the Cu­(II) spin number increased by *ca*. 20%, while the spectral features remained characteristic of site B. This recovery of the Cu­(II) signal is consistent with the generation of EPR-silent Cu­(I) during the 24 h incubation under anaerobic conditions via Cu­(II)-mediated Cys redox chemistry, followed by the reoxidation of Cu­(I) to Cu­(II) upon O_2_ exposure. It should be noted that Cu­(II) alone produced only a weak EPR signal under anaerobic conditions, whereas Cu­(II) coordinated to EDTA, similar to Cu­(II)–M-TTR, exhibited readily detectable EPR signals. These experiments support the assignment of sites A and B to Cu­(II) coordinated to M-TTR rather than to free or weakly associated Cu­(II) species in solution.

To further investigate O_2_-dependent oxidative modifications at the protein level, we conducted ESI–MS and tandem MS (ESI–MS^2^) measurements. As displayed in Figure 4b, the incubation of M-TTR with Cu­(II) under aerobic conditions for 24 h resulted in a pronounced peak at *ca*. 1,725 *m*/z, corresponding to a + 32 Da mass shift relative to unmodified M-TTR. Collision-induced dissociation (CID)^54–56^ analysis localized this modification to the *b*_10_ fragment (Figure 4c), identifying Cys10 as the site of oxidation and indicating the formation of Cys sulfinic acid (Figure 4b). In contrast, the fragmentation spectrum of the unmodified M- TTR peak (*ca*. 1,721 *m*/*z*) showed no corresponding mass shift. Notably, the +32 Da peak was negligible under anaerobic conditions and absent in the Cu­(II)-free samples even after O_2_ exposure (Figure S21), confirming that this oxidation arises specifically from O_2_-coupled Cu­(II)–Cys redox chemistry.

In addition to the +32 Da oxidative modification, the Cu­(II)-treated M-TTR samples incubated aerobically for 24 h exhibited new peaks at *ca*. 1,716 *m*/*z* and 1,714 *m*/*z* (Figure 4b), corresponding to mass losses of −34 Da and −46 Da relative to the unmodified protein. The −34 Da shift is consistent with β-elimination of a disulfide bond, generating dehydroalanine at Cys10.^57–61^ The −46 Da shift is assigned to the formation of a glycine (Gly) residue at position 10, indicating the modification of Cys10 to Gly.

These transformations are in line with known radical-mediated pathways involving Cys residues. Mechanistic studies using Cys-containing peptides have shown that thiyl radicals formed at Cys can undergo 1,2-hydrogen atom transfer to generate a C_β_-centered radical. In the presence of O_2_, this intermediate can be converted into peroxyl and alkoxyl radicals that promote cleavage of the C_α_–C_β_ bond.^57,60^ The resulting Gly radical can subsequently react with thiols to generate Gly residues. These findings demonstrate that Cu­(II)–Cys coordination triggers diverse O_2_-dependent oxidative transformations at the Cys residue, including the formation of sulfinic acid, dehydroalanine, and Gly. Collectively, these results reveal that O_2_-coupled Cu­(II)–Cys redox chemistry drives multiple oxidative pathways in proteins, generating covalent cross-links and site-specific oxidative modifications that contribute to higher-order protein assembly.

To evaluate whether these oxidative modifications arise from outer-sphere oxidation mediated by diffusible reactive oxygen species (ROS)^62,63^ or instead from localized, inner-sphere Cu-centered redox chemistry, we performed ESI–MS analyses of Cu­(II)-treated M-TTR incubated in the presence of DMSO or l-ascorbic acid, both of which have been widely used as ROS scavengers.^64,65^ As shown in Figures 4b and S22, the peak at *ca*. 1,725 *m*/z, corresponding to Cys sulfinic acid, remained the predominant oxidized species even in the presence of excess DMSO or l-ascorbic acid. These results indicate that scavenging diffusible ROS does not substantially suppress Cys oxidation, supporting the conclusion that the observed oxidative modifications predominantly proceed through localized Cu-centered inner-sphere redox chemistry rather than oxidation by freely diffusible ROS. It should be noted, however, that l-ascorbic acid can also act as a prooxidant in the presence of Cu;^66^ thus, its effects under these conditions should be interpreted with consideration of its potential dual roles as both a radical scavenger and a prooxidant.

In addition, the size distribution of Cu­(II)-treated M-TTR incubated in the presence of DMSO or l-ascorbic acid was analyzed by SDS–PAGE followed by Western blotting using an anti-TTR antibody (Figure S16). As depicted in Figure 4d, DMSO at concentrations up to 10% v/v produced little change in the size distribution of M-TTR species compared to the DMSO-free sample. Similarly, incubation with 1 equiv of l-ascorbic acid relative to M-TTR had minimal effect on the aggregation profile. Increasing the concentration of l-ascorbic acid to 10 equiv reduced the overall intensities of bands corresponding to M-TTR species ranging from the monomer to higher-order aggregates (*ca*. 11–135 kDa), compared with the sample without l-ascorbic acid. Together with the ESI–MS results, these findings indicate that the presence of DMSO or l-ascorbic acid did not substantially alter the oxidative modifications and aggregation pattern of M-TTR, supporting that localized Cu-centered oxidation following Cu­(II) coordination is the predominant pathway underlying these processes. It should be noted that M-TTR displayed comparable size distributions in the presence and absence of Cu­(II) in both HEPES and MOPS buffers, indicating that the observed aggregation is not attributable to HEPES-derived radical chemistry (Figure S23).^67^

As shown in Figure 3b-d, the treatment of M-TTR with Cu­(II) produced SDS-stable higher-order aggregates in addition to dimers. Because M-TTR contains only a single Cys residue, each protein molecule can form at most one intermolecular disulfide bond, limiting disulfide-mediated cross-linking to dimers. Moreover, aggregate formation was enhanced under aerobic conditions and persisted even after DTT treatment, particularly with 2 equiv of Cu­(II). These observations suggest that covalent cross-links other than disulfide bonds contribute to Cu­(II)-induced M-TTR aggregation. One potential source of such cross-linking is oxidative coupling of tyrosine (Tyr) residues to form dityrosine cross-links. M-TTR contains five Tyr residues (Tyr69, Tyr78, Tyr105, Tyr114, and Tyr116; Figure 1b) that are potential substrates for this reaction.

To test this possibility, we performed the dot blot assay using an anti-dityrosine antibody (1C3) on the samples of M-TTR incubated with Cu­(II) under aerobic or anaerobic conditions. As depicted in Figure 4e, noticeable 1C3 signals were detected only in the aerobic samples added with Cu­(II), whereas no signal was observed in the Cu­(II)-free samples or in the samples incubated under anaerobic conditions regardless of Cu­(II) treatment. Furthermore, as illustrated in Figure 4f and S24, the 1C3 signal increased progressively over time in the samples treated with 1 equiv of Cu­(II). In contrast, the samples treated with 2 equiv of Cu­(II) reached a near-maximal 1C3 signal immediately after Cu­(II) addition, which remained essentially unchanged throughout the incubation period. After 24 h, treatment with 2 equiv of Cu­(II) produced a *ca*. 30% stronger 1C3 signal than treatment with 1 equiv of Cu­(II). Overall results indicate that dityrosine cross-linking requires both Cu­(II) and O_2_, providing an additional covalent mechanism for Cu­(II)-induced M-TTR aggregation beyond disulfide bond formation.

### Protein Assembly and Proposed Mechanisms

In parallel with SDS–PAGE analyses (Figure 3b-d), the morphology of Cu­(II)-treated M-TTR aggregates was examined by transmission electron microscopy (TEM). As illustrated in Figure 5a, the treatment with Cu­(II) resulted in the formation of amorphous aggregates, whereas no comparable structures were observed in the absence of Cu­(II), demonstrating that Cu­(II) promotes M-TTR self-assembly. Under anaerobic conditions, the resultant aggregates were noticeably smaller than those observed under aerobic conditions, supporting that O_2_-dependent oxidative processes further enhance protein assembly. Together, SDS–PAGE and TEM analyses reveal that Cu­(II)–Cys coordination initiates M-TTR aggregation and that the presence of O_2_ markedly amplifies this process.

**Figure 5:**
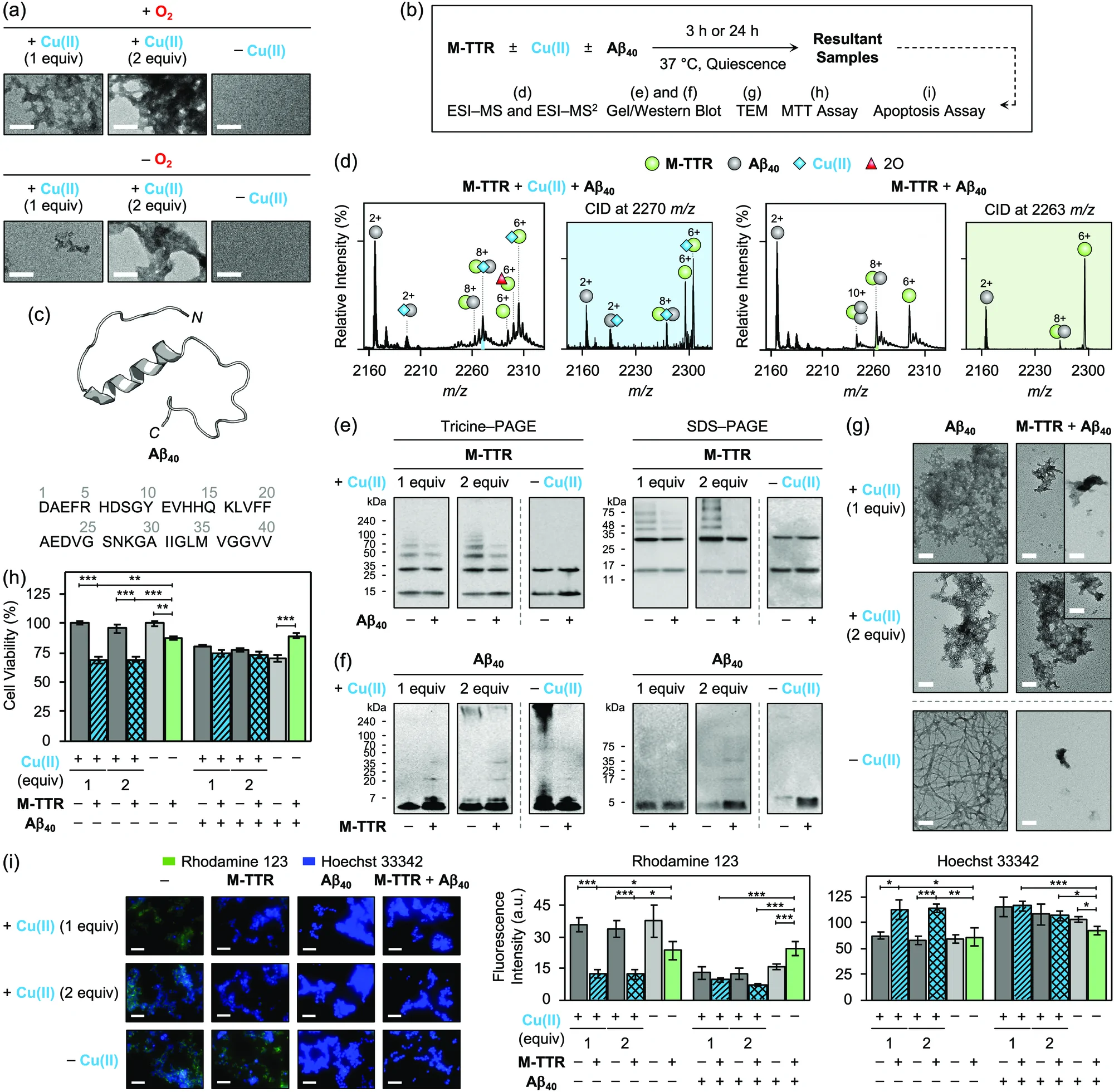
Cu­(II)-induced aggregation and dysfunction of M-TTR. (a) Resultant M-TTR aggregates monitored by TEM. Conditions: [M-TTR] = 25 μM; [CuCl_2_] = 25 μM and 50 μM; 20 mM HEPES, pH 7.4, 150 mM NaCl; 37 °C; 24 h incubation; quiescent conditions. Scale bars, 200 nm. (b) Scheme of the experiments. (c) Structure of monomeric Aβ_40_ determined by nuclear magnetic resonance spectroscopy (PDB 2LFM^19^). (d) M-TTR binding to Aβ_40_ with and without Cu­(II) monitored by ESI–MS and ESI–MS^2^. The peaks corresponding to [M-TTR + Cu­(II) + Aβ_40_]^8+^ (2,270 *m*/*z*; blue) and [M-TTR + Aβ_40_]^8+^ (2,263 *m*/*z*; green), observed in the mixture of M-TTR and Aβ_40_ with and without Cu­(II), respectively, were further characterized by ESI–MS^2^. Charge states are marked above the peaks in the mass spectra. Conditions: [M-TTR] = 100 μM; [Aβ_40_] = 100 μM; [CuCl_2_] = 100 μM; 20 mM ammonium acetate, pH 7.4; 37 °C; 3 h incubation; quiescent conditions. The samples were diluted 10-fold before injection into the mass spectrometer. The measurements were conducted in triplicate. (e and f) Size distribution of the resultant M-TTR and Aβ species with or without Cu­(II) investigated by tricine– and SDS–PAGE with Western blotting using anti-TTR and anti-Aβ (6E10) antibodies, respectively. Conditions: [M-TTR] = 25 μM; [Aβ_40_] = 25 μM; [CuCl_2_] = 25 μM and 50 μM; 20 mM HEPES, pH 7.4, 150 mM NaCl; 37 °C; 24 h incubation; quiescent conditions. The measurements were performed in triplicate. (g) Morphologies of the resultant peptide and protein aggregates detected by TEM. Scale bars, 200 nm. (h) Survival of the cells upon incubation of M-TTR, Cu­(II), Aβ_40_, or their mixtures following 24 h of preincubation. Cell viability, analyzed by the MTT assay, was calculated in comparison to that with an equivalent amount of the buffered solution (20 mM HEPES, pH 7.4, 150 mM NaCl). Conditions: [M-TTR] = 25 μM; [Aβ_40_] = 25 μM; [CuCl_2_] = 25 μM and 50 μM; nondifferentiated SH-SY5Y cells. The error bars denote s.e.m. for *n* = 6 examined over three independent experiments. (i) Overlay of the rhodamine 123- and Hoechst 33342-stained cells treated with or without M-TTR, Cu­(II), Aβ_40_, or their mixtures following 24 h of preincubation. The fluorescence intensity was quantified by the ImageJ software. The error bars represent s.e.m. for *n* = 22–138 cells. All statistical significance was determined by a two-sided unpaired Student’s *t*-test. *P < 0.05, **P < 0.01, and ***P < 0.001. Conditions: [M-TTR] = 25 μM; [Aβ_40_] = 25 μM; [CuCl_2_] = 25 μM and 50 μM; 20 mM HEPES, pH 7.4, 150 mM NaCl; nondifferentiated SH-SY5Y cells. Scale bars, 100 μm.

On the basis of the experimental observations described above, we propose a mechanistic framework for Cu­(II)-induced assembly of Cys-containing proteins, as illustrated in Figure 1c. In this model, Cu­(II) initially coordinates to the Cys S donor atom to form a Cu­(II)–Cys complex. This interaction promotes electron transfer from the Cys thiol group to Cu­(II),^68–70^ generating Cu­(I) together with a Cys-derived radical, such as a thiyl radical and/or a disulfide radical anion. These transient radical intermediates can initiate multiple covalent transformations,^57,60,71,72^ including disulfide bond formation, thereby promoting protein dimerization under anaerobic conditions.

Under aerobic conditions, the reoxidation of Cu­(I) by O_2_ sustains the Cu redox cycle, enabling additional oxidative transformations beyond disulfide formation, including dityrosine cross-linking, as well as oxidative transformations of Cys residues to sulfinic acid, dehydroalanine, and Gly. Overall, these reactions result in higher-order protein assembly and stabilize aggregated states. In addition to these covalent processes, other mechanisms may also contribute to Cu­(II)-driven protein assembly, including thioaldehyde-mediated cross-linking between Cys and neighboring residues (*e*.*g*., histidine, methionine, tryptophan, and lysine),^57,60,71,72^ radical-mediated cross-linking between oxidation-sensitive residues,^57,60,71,72^ intermolecular Cu coordination, and noncovalent protein–protein interactions.

### Reactivity toward Aβ

Recent studies have suggested that TTR variants may exert neuroprotective effects in AD.^73^ In the brains of AD patients, extracellular deposits arise from the aggregation of Aβ peptides into toxic oligomers and fibrils.^20^ M-TTR has been reported to regulate Aβ assembly.^27–29^ We therefore investigated whether Cu­(II)-induced M-TTR aggregation alters its anti-amyloidogenic activity (Figure 5b). Aβ_40_, the predominant Aβ isoform in cerebrospinal fluid, where TTR is abundant, was used in this study (Figure 5c).^19,74^ First, we examined direct interactions among M-TTR, Aβ_40_, and Cu­(II) by ESI–MS and ESI–MS^2^. As depicted in Figure 5d, the analysis of a mixture containing Aβ_40_ (*ca*. 4,330 Da), M-TTR (*ca*. 13,763 Da), and Cu­(II) revealed a peak at *ca*. 2,270 *m*/*z* corresponding to an 8+-charged species. ESI–MS^2^ analysis of this peak generated signals assigned to [M-TTR + Cu­(II)]^6+^ and [Aβ_40_ + Cu­(II)]^2+^, supporting the formation of a ternary complex composed of M-TTR, Aβ_40_, and Cu­(II). Furthermore, the CID of a peak at *ca*. 2,263 *m*/*z* detected in both Cu­(II)-treated and untreated samples produced multiply charged ions corresponding to M-TTR bound to Aβ_40_, indicating the presence of a heterodimeric complex, in agreement with previous reports.^27^

Next, we evaluated whether interactions among M-TTR, Aβ_40_, and Cu­(II) influence M-TTR aggregation. The samples containing M-TTR and Aβ_40_ in the presence of Cu­(II) were analyzed by tricine– and SDS–PAGE followed by Western blotting using an anti-TTR antibody. As presented in Figures 5e and S16, the size distribution and signal intensity of M-TTR aggregates above 35 kDa were reduced in the presence of Aβ_40_, indicating that Aβ_40_ partially suppresses Cu­(II)-induced M-TTR aggregation. In contrast, Aβ_40_ had minimal influence on M-TTR self-assembly in the absence of Cu­(II).

We then determined the effect of Cu­(II)-treated M-TTR on Aβ_40_ aggregation. Tricine–PAGE analysis of Aβ_40_ (4.3 kDa) incubated with M-TTR and 1 equiv of Cu­(II), followed by Western blotting using the Aβ antibody (6E10), revealed bands between 20–25 kDa and around 35 kDa (Figures 5f and S16). Increasing the concentration of Cu­(II) to 2 equiv further intensified these bands, while decreasing signal intensity above 240 kDa, suggesting a redistribution of aggregate populations. Under Cu­(II)-free conditions, M-TTR treatment of Aβ_40_ produced a band at 20–25 kDa and reduced smear intensity between 7–20 kDa and above 70 kDa, indicating a different mode of aggregation modulation.

To assess the stability of the resulting Aβ aggregates, SDS-PAGE analysis was performed. In the samples containing M-TTR and 1 equiv of Cu­(II), Aβ_40_ aggregates were barely detectable. In contrast, the treatment with 2 equiv of Cu­(II) produced clear bands below 5 kDa, at *ca*. 17 kDa, and between 25–35 kDa, suggesting the formation of SDS-stable oligomeric species likely corresponding to tetramers through octamers. In the absence of Cu­(II), the M-TTR-treated Aβ_40_ sample displayed smearing below 5 kDa, indicative of the formation of monomeric Aβ_40_ species that were not observed in the samples of Cu­(II)–Aβ_40_ without M-TTR. Together, these results indicate that M-TTR modulates Aβ aggregation in both Cu­(II)-free and Cu­(II)-containing environments but produces distinct aggregation outcomes.

Lastly, TEM revealed pronounced changes in aggregate morphology (Figure 5g). M-TTR incubated with Aβ_40_ and Cu­(II) (1 equiv) produced a mixture of short fibrils and amorphous aggregates. In contrast, the Cu­(II)–Aβ_40_ sample lacking M-TTR formed larger, unstructured aggregates. Increasing the Cu­(II) concentration to 2 equiv further promoted the formation of larger aggregates in the sample containing both M-TTR and Aβ_40_. Under Cu­(II)-free conditions, M-TTR added with Aβ_40_ yielded small amorphous aggregates, whereas metal-free Aβ_40_ alone formed large fibrillar structures, consistent with previous reports describing the ability of M-TTR to modulate Aβ_40_ aggregation.^27,28,31^ Thus, these results demonstrate that Cu­(II)–Cys redox chemistry alters the modulatory effect of M-TTR on Aβ_40_ aggregation.

### Cellular Responses

To verify the biological consequences of M-TTR incubated with Cu­(II), human neuroblastoma SH-SY5Y (5Y) cells were treated with M-TTR preincubated with or without Cu­(II) for 24 h (Figure 5b). Cell viability was then assessed using the MTT assay [MTT = 3-(4,5-dimethylthiazol-2-yl)-2,5-diphenyltetrazolium bromide]. As depicted in Figure 5h, the treatment with Cu­(II)-incubated M-TTR reduced cell viability to approximately 70%, whereas cells added with Cu­(II)-untreated M-TTR maintained viability above 85%. Although these experiments do not distinguish the respective contributions of Cu­(II)-induced protein modifications and residual ROS that may be present in the treated samples, they demonstrate that the products generated during Cu­(II) incubation exhibit enhanced cytotoxicity under the experimental conditions.

To further investigate the mechanism of cytotoxicity, we examined apoptotic responses, which are characterized by the loss of mitochondrial membrane potential and nuclear condensation.^75,76^ Early apoptosis was evaluated using rhodamine 123 to monitor mitochondrial membrane potential, while late-stage apoptosis was assessed using Hoechst 33342 staining of condensed nuclear DNA.^76–78^ Cells treated with Cu­(II)–M-TTR displayed decreased rhodamine 123 fluorescence and increased Hoechst 33342 fluorescence relative to cells added with Cu­(II)-untreated M-TTR (Figures 5i and S25), indicating the loss of mitochondrial membrane potential and DNA condensation, respectively. In contrast, Cu­(II) alone or M-TTR alone produced minimal changes compared with the metal-free buffered control. These observations confirm that Cu­(II)-treated M-TTR promotes apoptotic cell death.

We next investigated whether Cu­(II)-treated M-TTR modulates Aβ_40_-induced cytotoxicity. As displayed in Figure 5h, M-TTR did not significantly attenuate the cytotoxicity induced by Cu­(II)–Aβ_40_. Under metal-free conditions, however, the preincubation with M-TTR increased cell survival by *ca*. 20%, consistent with its reported protective effect against Aβ toxicity.^27–33,73^ Fluorescence imaging using rhodamine 123 and Hoechst 33342 supported these observations (Figures 5i and S25). While the samples of Cu­(II)–Aβ_40_ exhibited comparable apoptotic responses regardless of the presence of M-TTR, M-TTR alleviated apoptosis in cells treated with metal-free Aβ_40_, as indicated by increased rhodamine 123 fluorescence and reduced Hoechst 33342 fluorescence.

Moreover, M-TTR was preincubated with Cu­(II) for 24 h before the addition of Aβ_40_ for a further 24 h to distinguish the effects of prior Cu­(II)-mediated oxidation of M-TTR from those of simultaneous exposure to Cu­(II) and Aβ_40_. As illustrated in Figure S26b, the preincubation of M-TTR with Cu­(II) (condition A in Figure S26a) failed to suppress Aβ_40_-induced cytotoxicity, yielding cell viability comparable to that observed under simultaneous incubation conditions (condition B in Figure S26a). Consistent with these findings, as shown in Figures S25 and S26c, apoptosis analysis showed no reduction in Aβ_40_-induced apoptotic cell death following prior Cu­(II)-mediated oxidation of M-TTR.

Collectively, these results demonstrate that Cu­(II)-treated M-TTR, together with its oxidized and aggregated forms, no longer suppresses Aβ_40_-induced cytotoxicity or apoptosis under our experimental conditions. Notably, the SDS-stable Aβ_40_ oligomers formed in the presence of both Cu­(II) and M-TTR (*e*.*g*., tetramers to octamers; Figure 5f) correspond to aggregation states previously associated with neurotoxicity.^79,80^ These findings indicate that Cu­(II) treatment compromises the protective effect of M-TTR observed under metal-free conditions, thereby permitting the formation of neurotoxic Aβ_40_ assemblies and the resulting apoptotic response. It should be noted, however, that because M-TTR, Cu­(II), and Aβ can form ternary complexes (Figure 5d), the Cu­(II)/O_2_-mediated chemistry occurring in the presence of Aβ may differ from that characterized for M-TTR alone. Accordingly, the biological effects observed in the ternary system cannot be attributed solely to the oxidative modifications identified for M-TTR in the absence of Aβ. In addition, the present study establishes a proof-of-principle mechanistic framework primarily under simplified *in vitro* conditions, with the influence of serum on the cell viability assay further examined in Figure S27. Given that physiological environments contain competing metal-binding proteins, antioxidants, and numerous other factors that affect metal speciation and protein oxidation, the pathophysiological relevance of Cu­(II)–M-TTR interactions remains to be determined through future studies under more physiologically and disease-relevant conditions.

## Conclusions

In this work, we establish a protein-based platform that directly captures O_2_-coupled Cu­(II)–Cys redox chemistry and defines its structural and functional consequences. Using M-TTR as a tractable model, we show that Cu­(II) coordination to a Cys residue initiates the formation of thiyl radical and/or disulfide radical anion, triggering a cascade of oxidative transformations that generate disulfide bonds and O_2_-dependent modifications, including sulfinic acid, dehydroalanine, glycine, and dityrosine cross-links. These chemically defined events drive higher-order protein assembly and compromise the native anti-amyloidogenic function of the protein. The use of M-TTR, which contains a single solvent-exposed Cys residue, enabled this reaction sequence to be delineated with minimal mechanistic ambiguity. Although proteins containing multiple Cu-binding sites, thiols, or other oxidizable residues are expected to exhibit more complex and competing redox pathways, the fundamental O_2_-coupled Cu­(II)–Cys redox chemistry established here provides a mechanistic basis for understanding how localized metal–thiol redox reactions initiate oxidative protein modifications and assembly in more complex biological systems.

Beyond this model system, our findings establish a general chemical principle in which O_2_-coupled metal–thiol redox chemistry drives protein oxidation and higher-order assembly. Specifically, we demonstrate that redox-active metal ions promote radical-mediated oxidative modifications, leading to protein structural alteration, higher-order assembly, and cytotoxicity. More broadly, the platform introduced here provides a foundation for systematically interrogating metal–thiol reactivity in complex biological environments and offers mechanistic insight into how localized metal-centered redox chemistry contributes to protein dysfunction associated with neurodegenerative and oxidative stress-associated diseases.

## Supplementary Material



## Notes

Experimental details, characterization of Cu­(II)-bound protein species, DFT models for Cu­(II)-binding modes, TDDFT-simulated Abs spectra, additional gel/Western and dot blot analyses, MS analyses under various conditions, and cell-based studies (PDF)
