# Supplementary material for: O2‑Coupled Copper–Cysteine Redox Chemistry Drives Oxidative Modifications and Higher-Order Assembly of Monomeric Transthyretin
Source: ACS Cent Sci. 2026 Oct 5;12(10):1659–71. doi: 10.1021/acscentsci.6c00619 (PMC13720927; doi:10.1021/acscentsci.6c00619)
Supplement: Supplementary file 1 [file oc6c00619_si_001.pdf]

Supporting Information

for

O_2-Coupled Copper–Cysteine Redox Chemistry Drives Oxidative Modifications and Higher-order Assembly of Monomeric Transthyretin

Yelim Yi[1], Wooyeol Ryu[1], Bokyung Kim[2], Srinivasan Muniyappan[2], Jin Hae Kim[2]*, Kiyoung Park[1]*, and Mi Hee Lim[1]*

[1]Department of Chemistry, Korea Advanced Institute of Science and Technology (KAIST), Daejeon 34141, Republic of Korea

[2]Department of New Biology, Daegu Gyeongbuk Institute of Science and Technology (DGIST), Daegu 42988, Republic of Korea

*To whom correspondence should be addressed: miheelim@kaist.ac.kr, kiyoung.park@kaist.ac.kr, and jinhaekim@dgist.ac.kr

Table of contents

Experimental section

Materials and methods	S4
Preparation of M-TTR	S4
Electronic absorption (Abs) spectroscopy	S5
Electron paramagnetic resonance (EPR) spectroscopy	S6
Electrospray ionization–mass spectrometry (ESI–MS)	S7
Computational details	S7
Gel electrophoresis with Western blotting (Gel/Western blot)	S8
Dot blot assay	S9
Transmission electron microscopy (TEM)	S9
Preparation of Aβ	S10
Cell viability studies	S10
Apoptosis measurements	S11

**Table S1**	Abs and EPR spectroscopic features of Cu(II)-containing enzymes and synthetic complexes	S12
**Table S2**	DFT-calculated EPR parameters of Cu(II)–M-TTR models in Figures S7–S13	S13

**Figure S1**	Analysis of $CuCl_2$ treated with M-TTR by electronic Abs spectroscopy	S14
**Figure S2**	Cu(II) binding to M-TTR	S15
**Figure S3**	EPR spectra decomposition	S16
**Figure S4**	DFT models for the Cu(II) sites of nitrosocyanin	S17
**Figure S5**	TDDFT calculations of the nitrosocyanin models	S19
**Figure S6**	Calibration of DFT-calculated spectroscopic properties	S20
**Figure S7**	DFT models for Cu(II) binding to site 1 in M-TTR without Met13 ligation	S21
**Figure S8**	TDDFT-simulated Abs spectra of the site 1 models without Met13 coordination	S22
**Figure S9**	DFT models for Cu(II) binding to site 1 in M-TTR with Met13 coordination	S23
**Figure S10**	TDDFT-simulated Abs spectra of the site 1 models with Met13 coordination	S24
**Figure S11**	DFT models for Cu(II) binding to site 1 in M-TTR without Cys10 coordination	S25
**Figure S12**	TDDFT-simulated Abs spectra of the site 1 models without Cys10 coordination	S26
**Figure S13**	DFT models for Cu(II) binding to site 2 or 3 in M-TTR	S27
**Figure S14**	TDDFT-simulated Abs spectra of the site 2 models	S28
**Figure S15**	TDDFT-simulated Abs spectra of the site 3 models	S29

**Figure S16**	Unprocessed gel/Western blot data from Figures 3, S23, and 5	S30
**Figure S17**	Characterization of the sample of M-TTR	S31
**Figure S18**	Abs spectra of BCA-containing samples incubated with M-TTR and Cu(II) for 2 h	S32
**Figure S19**	EPR spectra of Cu(I/II), TEMPO, and their mixtures without O_2	S33
**Figure S20**	EPR spectra of $CuCl_2$, M-TTR, and their mixtures under anaerobic conditions	S34
**Figure S21**	MS spectra of M-TTR incubated without Cu(II) under aerobic conditions	S35
**Figure S22**	MS spectra of Cu(II)-treated M-TTR incubated with either DMSO or L-ascorbic acid under aerobic conditions	S36
**Figure S23**	Size distribution of the M-TTR incubated with or without Cu(II) in MOPS buffer probed by SDS–PAGE with Western blotting using an anti-TTR antibody	S37
**Figure S24**	Dot blots of Cu(II)-treated and -untreated protein samples using an anti-TTR antibody	S38
**Figure S25**	Apoptosis of the cells treated with or without M-TTR, Cu(II), $A\beta_{40}$, or their mixtures	S39
**Figure S26**	Cell studies for M-TTR, Cu(II), $A\beta_{40}$, or their mixtures	S40
**Figure S27**	Survival of 5Y cells treated with M-TTR, Cu(II), $A\beta_{40}$, or their mixtures in serum-containing media	S41

References S42

Experimental section

Materials and methods. All chemical reagents were purchased from commercial suppliers and used as received unless otherwise stated. Sodium phosphate and Tris-HCl [Tris(hydroxymethyl)aminomethane hydrochloride] were acquired from PanReac AppliChem (Darmstadt, Germany). The buffered solution was prepared in doubly distilled water [ddH_2O; Milli-Q Direct 16 system (18.2 MΩ·cm; Merck KGaA, Darmstadt, Germany)]. Trace metal contamination was removed from all solutions used for experiments by treating with Chelex (Sigma-Aldrich) overnight. The samples were prepared using Eppendorf tubes (Eppendorf, Hamburg, Germany) unless otherwise stated. ESI–MS experiments were performed by an Agilent 6530 Accurate Mass Quadrupole Time-of-Flight mass spectrometer with an ESI source (Agilent, Santa Clara, CA, USA). Abs spectra were recorded on an Agilent 8453 ultraviolet–visible spectrophotometer (Agilent). X-band continuous-wave EPR spectra were obtained with a Bruker EMXplus EPR spectrometer (Bruker BioSpin, Billerica, MA, United States) equipped with an ER 4141VT digital temperature control system (Bruker BioSpin) and an ER 4119HS cavity (Bruker BioSpin). The amount of copper in the sample of M-TTR was measured by an iCAP RQ ICP–MS [Thermo Fisher Scientific, Waltham, MA, USA; KAIST Analysis Center for Research Advancement (KARA), Daejeon, Republic of Korea]. Images obtained by gel/Western blot and the dot blot assay were visualized by a ChemiDoc MP imaging system (Bio-Rad, Hercules, CA, USA). Morphologies of peptide and protein aggregates produced from the aggregation experiments were taken on a Tecnai F20 transmission electron microscope (FEI Company, Eindhoven, Netherlands; KARA). Synthetic $A\beta_{40}$ (DAEFRHDSGYEVHHQKLVFFAEDV-GSNKGAIIGLMVGGVV) was obtained from Peptide Institute (Osaka, Japan) and purified by high-performance liquid chromatography using YMC Pack ODS-A (YMC, Kyoto, Japan) and Imtakt Cadenza CD-C18 columns (Imtakt, Portland, OR, USA). HEPES [2-(4-(2-hydroxyethyl)piperazin-1-yl)ethanesulfonic acid] was purchased from Sigma-Aldrich (St. Louis, MO, USA). The Abs values for cell viability studies were determined by a microplate reader (SpectraMax M5; Molecular Devices, Sunnyvale, CA, USA). The fluorescence imaging required for analyzing apoptosis was conducted by an EVOS FL fluorescence microscope (Advanced Microscopy Group, Bothell, WA, USA).

Preparation of M-TTR. M-TTR (GPTGTGESKCPLMVKVLDAVRGSPAINVAVHVFRKAADDTW-EPFASGKTSESGELHGLTTEEEFVEGIYKVEIDTKSYWKALGISPMHEHAEVVFTANDSGPRRY TIAAMLSPYSYSTTAVVTNPKE) was recombinantly expressed from *Escherichia coli* (*E. coli*) as a fusion protein with a tobacco etch virus (TEV)-cleavable 6x-His tag at the *N*-terminus. We

ensured that the recombinant M-TTR construct does not have any additional amino acids other than the original sequence. Following the previously reported methods, the proteins were expressed in the *E. coli* M15(pREP4) competent cells that were transformed with the pQE30 vector (Qiagen, Hilden, Germany) encoding M-TTR.[1] The competent cells were inoculated into Luria-Bertani (LB) broth (3 mL) and diluted into fresh media (1 L). After reaching an optical density of 0.6 at 600 nm, protein expression was induced by isopropyl β-D-1-thiogalactopyranoside (1 mM; PanReac AppliChem, Darmstadt, Germany) at 37 °C overnight. The final cell pellets were obtained with centrifugation at 4,000 *g* for 20 min at 4 °C and stored at –80 °C until used. The proteins were purified by the resuspension of cell pellets in 20 mM sodium phosphate, pH 7.4, 500 mM NaCl, 30 mM imidazole (PanReac AppliChem). The fully resuspended cells were disrupted by sonication, and the resultant lysate was centrifuged at 40,000 *g* for 40 min at 4 °C to remove any insoluble cell debris. The clear supernatant was then applied to a nickel affinity column (HisTrap HP; Cytiva, Marlborough, MA, USA) connected to an ÄKTAprime plus fast protein liquid chromatography (FPLC) system (Cytiva). Target proteins were eluted with the solution of imidazole (30–500 mM). To remove the 6x-His tag, the eluted fractions containing the target proteins were dialyzed at 25 °C overnight in 50 mM Tris-HCl, pH 7.5, 0.5 mM EDTA (ethylenediaminetetraacetic acid; PanReac AppliChem), 1 mM DTT (1,4-dithiothreitol; PanReac AppliChem), followed by incubation with TEV protease under the same conditions. The reaction mixture was re-applied to a HisTrap HP column, from which the flow-through fraction was collected. To remove any metal contaminants and disulfide bonds, EDTA (0.5 mM) and DTT (1 mM) were again added to the fraction. This fraction was finally applied to a gel filtration column (HiLoad 16/60 Superdex 75 column; Cytiva) connected to an ÄKTA go FPLC system (Cytiva) in 20 mM HEPES, pH 7.4, 150 mM NaCl. The final pure sample was concentrated, aliquoted, flash-frozen with liquid nitrogen, and stored at –80 °C until used. The fractions from each purification step were monitored by sodium dodecyl sulfate–polyacrylamide gel electrophoresis (SDS–PAGE). The concentration of M-TTR solution was determined by measuring the Abs at 280 nm (ε = 18,450 $M^{-1}cm^{-1}$).[2]

Abs spectroscopy. The Abs spectrum of $CuCl_2$ (100 μM) was obtained in 20 mM HEPES, pH 7.4, 150 mM NaCl. To confirm Cu(II) binding to M-TTR, the solution of $CuCl_2$ (100 μM) and M-TTR (100 μM) was prepared in the buffered solution, and the spectral change was monitored at 37 °C. To measure the K_d value for Cu(II)(M-TTR), M-TTR (100 μM) was titrated with various concentrations of $CuCl_2$ (4–126 μM) in the buffered solution. The change in the Abs at 380 nm

was plotted as a function of [Cu(II)]. Data are presented as mean ± s.e.m. for n = 3. The K_d value of M-TTR for Cu(II) was estimated using a non-linear curve fitting in OriginPro 9.6 software (OriginLab, Northampton, MA, USA) based on equation (1)[3]. Note that ΔAbs and ΔAbs_{max} represent the alteration of Abs at 380 nm and its maximum value, respectively.

$$\frac{\Delta Abs}{\Delta Abs_{max}} = \frac{K_d + [\text{M-TTR}]_{total} + [\text{Cu(II)}]_{total} - \sqrt{(K_d + [\text{M-TTR}]_{total} + [\text{Cu(II)}]_{total})^2 - 4 \times [\text{M-TTR}]_{total} \times [\text{Cu(II)}]_{total}}}{2 \times [\text{M-TTR}]_{total}} \quad (1)$$

The binding stoichiometry of M-TTR for Cu(II) was determined by Job's method of continuous variation.[4] Mixtures of M-TTR (45, 90, 112.5, 149.9, 180, 225, 270, 300, 337.5, 360, and 450 μM) and $CuCl_2$ (45, 90, 112.5, 149.9, 180, 225, 270, 300, 337.5, 360, and 450 μM) were prepared in 20 mM HEPES, pH 7.4, 150 mM NaCl while maintaining a constant total concentration of M-TTR and Cu(II) (450 μM). The changes in Abs at 380 nm were measured, and the resulting values were plotted as a function of the mole fraction of Cu(II) to obtain a Job plot.

Cu(I) formation in the presence of M-TTR and Cu(II) was monitored using the BCA assay (BCA = 2,2′-biquinoline-4,4′-dicarboxylic acid; Sigma-Aldrich). M-TTR (25 μM), $CuCl_2$ (25 μM), and BCA (100 μM) were mixed in 20 mM HEPES, pH 7.4, 150 mM NaCl and incubated at room temperature for up to 2 h. Electronic Abs spectra were recorded immediately after mixing and after 2 h of incubation. The concentration of $Cu(I)(BCA)_2$ was quantified from its absorbance at 562 nm using an extinction coefficient (ε) of 7,700 $M^{-1}cm^{-1}$.[5]

EPR spectroscopy. $CuCl_2$ (100 μM) was mixed with M-TTR (100 μM) in 20 mM HEPES, pH 7.4, 150 mM NaCl, and the samples were either frozen immediately or incubated for 24 h at 37 °C under quiescent conditions in the absence and presence of O_2 prior to freezing. To monitor protein-centered radical generation, TEMPO [(2,2,6,6-tetramethylpiperidin-1-yl)oxyl; Sigma-Aldrich] was employed as a radical scavenger. Solutions containing $CuCl_2$ (100 μM) or $[Cu(CH_3CN)_4]PF_6$ (100 μM) and TEMPO (100 μM; with or without 0.2% v/v CH_3CN) were mixed with and without M-TTR (100 μM) under quiescent conditions in an N_2-filled glove box. The resulting samples were immediately frozen without prolonged incubation and subsequently analyzed by EPR spectroscopy using the following experimental parameters: microwave frequency, 9.4 GHz; microwave power, 1 mW; modulation frequency, 100 kHz; modulation amplitude, 10 G; time constant, 0.01 ms; conversion time, 38 ms; sweep time, 60 s; number of scans, 4; temperature, 100 K. All EPR spectra were simulated using the W95EPR program

developed by F. Neese.[6] The loss of TEMPO spin resulting from radical scavenging was quantified using equation (2). Spin quantification by double integration was performed only over the magnetic field range corresponding to the TEMPO signal. Note that $S_{TEMPO+Cu+M\text{-}TTR}$, $S_{Cu+M\text{-}TTR}$, $S_{TEMPO+Cu}$, and S_{Cu} denote the spin numbers of the corresponding samples.

$$Loss\ of\ spin\ by\ radical\ scavenging\ (\%) = \left(1 - \frac{S_{TEMPO+Cu+M\text{-}TTR} - S_{Cu+M\text{-}TTR}}{S_{TEMPO+Cu} - S_{Cu}}\right) \times 100 \quad (2)$$

ESI–MS. M-TTR (100 μM) was incubated with or without $CuCl_2$ (100 μM) in the absence and presence of $A\beta_{40}$ (100 μM), DMSO (1% or 10% v/v; Sigma-Aldrich), or L-ascorbic acid (100 μM and 1000 μM; Sigma-Aldrich) for 3 or 24 h at 37 °C under quiescent conditions in 20 mM ammonium acetate, pH 7.4 (Sigma-Aldrich). To monitor the chemical modifications of Cu(II)-added M-TTR under anaerobic conditions, M-TTR (100 μM) was treated with or without $CuCl_2$ (100 μM) in 20 mM ammonium acetate, pH 7.4 for 24 h at 37 °C under quiescent conditions in a N_2-filled glove box. Before injection into the mass spectrometer, the resultant samples were diluted 10-fold with LC-grade H_2O. The capillary voltage, the drying gas flow, and the gas temperature were set to 5.8 kV, 12 L/min, and 300 °C, respectively. The ESI parameters and experimental conditions for ESI–MS^2 were the same as above. The collision-induced dissociation was conducted by applying the collision energy at 30–82 eV. The measurements were conducted in triplicate.

Computational details. To verify the reliability of DFT methods in reproducing spectroscopic data, nitrosocyanin, a structurally well-characterized red copper enzyme, was selected as a reference system for method calibration. The X-ray crystal structure of nitrosocyanin (PDB 1IBY[7]) contains four monomers, whose active sites differ slightly by the positions of co-crystalized 1,6-hexanediol molecules and thus hydrogen bonds to the coordinated water molecules. The hydrogen-bonded alcohol was replaced with a water molecule in DFT models. All four sites were truncated by capping the backbone atoms adjacent to α-carbons with hydrogen atoms, which were frozen during geometry optimization. Truncated structures of nitrosocyanin were optimized using four distinct DFT functionals, and their Abs spectra were simulated through time-dependent (TD) DFT calculations (Figures S4 and S5). Although all tested DFT methods resulted in overestimated Cu–S bond lengths that varied depending on the presence of hydrogen bonds involving the coordinated water molecule, the computed energies of the S-to-Cu CT transition showed minimal sensitivity to bond-length variations, primarily influenced instead by the choice

of DFT functionals. Among the evaluated functionals, the M06 functional most accurately reproduced experimental spectroscopic data. Its systematic tendencies to slightly overestimate S-to-Cu CT transition energies and underestimate *d-d* transition energies were noted and factored into subsequent modeling of the M-TTR site A (Figure S6).

The same truncation scheme was used to prepare DFT models for Cu(II)-binding sites in M-TTR, which were obtained from three different Zn(II)-binding sites of TTR (PDB 3GRG[8]). Site 1 contained the Cys10, Met13, His56, and Gly57 residues. The His88, His90, and Glu92 residues were included in site 2. Site 3 had the His31, Glu72, and Asp74 residues. As the sites were exposed to solvent, water molecules were added to the models for possible hydrogen-bond interactions. For geometry optimization and TDDFT[9] calculations, the Gaussian 09-d.01 package[10] was used with the M06,[11] BP86[12,13] with 38% or 50% HF exchange[14] and B3LYP[15] functionals, the 6-31g(d) basis set,[16–20] and the PCM (water) solvation model.[21] For comparing models with different number of protons, the solvation energy of proton, $\Delta_s G°(H^+)$ = –265.9 kcal/mol, was utilized.[22] For EPR parameter calculations, the ORCA 4.2.0 software[23,24] was employed along with the B3LYP[15] functional and the mixed basis sets of CP(PPP)[25] for copper, EPR-III[26] for the coordinated nitrogen, oxygen, and sulfur donor atoms, and 6-31g(d)[16–20] for others.

Gel/Western blot. M-TTR (25 μM) was incubated with or without $CuCl_2$ (25 μM and 50 μM), DMSO (1% and 10% v/v), or L-ascorbic acid (25 μM and 250 μM) for 24 h at 37 °C under quiescent conditions in 20 mM HEPES or MOPS, pH 7.4, 150 mM NaCl. In a similar manner, M-TTR (25 μM) and $A\beta_{40}$ (25 μM) were coincubated with or without $CuCl_2$ (25 μM and 50 μM) for 24 h at 37 °C under quiescent conditions in the buffered solution. To investigate the formation of disulfide bond after the removal of Cu, M-TTR (25 μM) incubated with $CuCl_2$ (25 μM and 50 μM) for 24 h was treated with Chelex for 30 min at 4 °C with gentle mixing. The resin was subsequently pelleted by brief centrifugation, and the supernatant was carefully collected for subsequent DTT treatment. To analyze the aggregation of M-TTR in the presence of $CuCl_2$ under anaerobic conditions, M-TTR (25 μM) was treated with or without $CuCl_2$ (25 μM and 50 μM) in the buffered solution for 24 h at 37 °C under quiescent conditions in a N_2-filled glove box. Each sample (10 μL) was separated onto a tricine gel (10–20% w/v acrylamide; Invitrogen, Carlsbad, CA, USA). For the SDS–PAGE, each sample (5 μL) was denatured with SDS (5% w/v; 5 μL; Wako Chemicals, Osaka, Japan) with or without DTT (200 mM; Sigma-Aldrich) and heated for 5 min at 95 °C. The resultant samples were resolved through the homemade gel (5% w/v acrylamide for stacking gels; 12% w/v

acrylamide for resolving gels; 10% w/v SDS). Following separation, the proteins and peptides were transferred onto nitrocellulose membranes and blocked with bovine serum albumin (BSA, 3% w/v; Sigma-Aldrich) in Tris-buffered saline (TBS) containing 0.1% v/v Tween-20 (Sigma-Aldrich) (TBS-T) for 4 h at room temperature or overnight at 4 °C. The membranes were incubated with an anti-TTR antibody (1:3,000; Thermo Fisher Scientific, Waltham, MA, USA) or 6E10 (1:20,000; Biolegend, San Diego, CA, USA) in the solution of BSA (2% w/v in TBS-T) at room temperature (1 h for anti-TTR antibody; 4 h for 6E10). After washing with TBS-T three times (10 min), a horseradish peroxidase-conjugated goat anti-rabbit (1:15,000 for anti-TTR; Promega, Madison, WI, USA) or anti-mouse (1:7,500 for 6E10; Cayman, Ann Arbor, MI, USA) secondary antibody in the solution of BSA (2% w/v in TBS-T) was added for 1 h at room temperature. After washing with TBS-T three times (10 min), a homemade ECL kit[27] was used to visualize the images gained by gel/Western blot on a ChemiDoc MP Imaging System (Bio-Rad, Hercules, CA, USA). The signals from each lane of the gel images were quantified by the Image Lab software (Bio-Rad). The measurements were conducted in triplicate. For statistical analysis, Student's *t*-test was used.

Dot blot assay. M-TTR (25 μM) was incubated with or without $CuCl_2$ (25 μM and 50 μM) for 0.2, 0.5, 1, 2, 4, 6, 8, 12, and 24 h at 37 °C under quiescent conditions in 20 mM HEPES, pH 7.4, 150 mM NaCl. The resultant solutions (5 μL) were spotted on nitrocellulose membranes and blocked with BSA (3% w/v; Sigma-Aldrich) in TBS containing 0.1% v/v Tween-20 (0.1% TBS-T; Sigma-Aldrich) for 2 h at room temperature or overnight at 4 °C. The membranes were incubated with an anti-dityrosine antibody (1C3; 1:1,000; Japan Institute for the Control Aging, Nikken SEIL, Shizuoka, Japan) or anti-TTR antibody (1:3,000; Thermo Fisher Scientific) in the solution of BSA (2% w/v in 0.1% TBS-T for 1C3; in 0.1% TBS-T for the anti-TTR antibody) overnight at room temperature. After washing with 0.1% TBS-T five times (5 min), a horseradish peroxidase-conjugated goat anti-mouse (1:5,000 for 1C3; Cayman) or anti-rabbit (1:15,000 for the anti-TTR antibody; Promega) secondary antibody in the solution of BSA (2% w/v in 0.1% TBS-T) was added for 1 h at room temperature. After washing with 0.1% TBS-T five times (5 min), a homemade ECL kit[27] was used to visualize the results on a ChemiDoc MP Imaging System (Bio-Rad). The signals were quantified by the Image Lab software (Bio-Rad). The measurements were conducted in triplicate.

TEM. Samples were prepared following the procedure described in gel/Western blot. Glow-discharged grids (Formvar/Carbon 300-mesh; Electron Microscopy Sciences, Hatfield, PA, USA)

were treated with the samples containing either M-TTR species, Aβ_{40} species, or their mixture with or without $CuCl_2$ (25 μM for proteins or peptides; 25 μM and 50 μM for $CuCl_2$; 5 μL) for 2 min at room temperature. Excess sample was removed using filter paper followed by washing with ddH_2O three times. Each grid incubated with uranyl acetate (1% w/v, aq; 5 μL; Polysciences, Warrington, PA, USA) for 2 min at room temperature was blotted off and dried overnight at room temperature. The location of the samples on the grids was randomly picked, taking more than 20 images per grid.

Preparation of Aβ. Aβ_{40} was dissolved in NH_4OH (1% w/w, aq; Sigma-Aldrich, St. Louis, MO, USA). The solutions were aliquoted, lyophilized overnight, and stored at –80 °C. A stock solution of Aβ_{40} was prepared by dissolving the lyophilized peptide with NH_4OH (1% w/w, aq; 10 μL) followed by addition of ddH_2O, as previously reported.[28] The concentration of the solution containing Aβ_{40} was determined by measuring the Abs at 280 nm (ε = 1,450 $M^{-1}cm^{-1}$).[28]

Cell viability studies. The human neuroblastoma SH-SY5Y (5Y) cell line was purchased from the American Type Culture Collection (Manassas, VA, USA). 5Y cells were maintained in the media containing 50% v/v minimum essential medium (MEM; GIBCO, Grand Island, NY, USA) and 50% v/v F-12 (GIBCO) supplemented with 10% v/v fetal bovine serum (FBS; Sigma-Aldrich) and 100 U/mL penicillin with 100 mg/mL streptomycin (GIBCO). The cells were grown and maintained at 37 °C in a humidified atmosphere with 5% CO_2. Cell viability was determined by the MTT assay [MTT = 3-(4,5-dimethylthiazol-2-yl)-2,5-diphenyltetrazolium bromide; Biosesang, Seoul, Republic of Korea]. M-TTR (125 μM) with or without $CuCl_2$ (125 and 250 μM) in the absence and presence of Aβ_{40} (125 μM) was preincubated for 24 h or 48 h at 37 °C under quiescent conditions in 20 mM HEPES, pH 7.4, 150 mM NaCl. The samples of M-TTR (125 μM) preincubated with $CuCl_2$ (250 μM) for 24 h at 37 °C under quiescent conditions in 20 mM HEPES, pH 7.4, 150 mM NaCl were also prepared, followed by an additional 24 h incubation with Aβ_{40} (125 μM) at 37 °C under quiescent conditions in the buffered solution.

Cells were seeded in a 96-well plate (50,000 cells/100 μL; SPL Life Sciences, Pocheon, Gyeonggi, Republic of Korea) and incubated for 24 h. The cells were washed with PBS (pH 7.4; GIBCO) and subsequently washed with either serum-free medium (50% v/v MEM and 50% v/v F-12 supplemented with 100 U/mL penicillin with 100 mg/mL streptomycin)[29] or serum-containing medium (50% v/v MEM and 50% v/v F-12 supplemented with 10% v/v FBS and 100 U/mL penicillin with 100 mg/mL streptomycin). The cells were then treated with the samples (final

concentrations, 25 μM for proteins or peptides; 25 μM and 50 μM for $CuCl_2$). After 24 h or 36 h incubation, the cells were washed with PBS (pH 7.4) and serum-free or serum-containing media. MTT [5 mg/mL in PBS (pH 7.4); 25 μL] was added to each well, and the plate was incubated for 3 h at 37 °C. Formazan produced by cells was solubilized using an acidic solution of *N*,*N*-dimethylformamide (pH 4.5, 50% v/v, aq; Daejung Chemicals, Busan, Republic of Korea) and SDS (20% w/v; Wako Chemicals) at room temperature overnight in the dark. Abs was measured at 600 nm by a microplate reader (Molecular Devices, Sunnyvale, CA, USA). Cell viability was calculated, compared to that of the cells treated with an equivalent amount of the buffered solution. Data are presented as mean ± s.e.m. for n = 6 examined over three independent experiments. For the statistical analysis, Student's *t*-test was used.

Apoptosis measurements. The samples were prepared and treated with the cells plated on a 96-well black wall/clear bottom plate (SPL Life Sciences) following the procedure described in cell viability studies. After 36 h incubation, the cells were washed with PBS (pH 7.4; GIBCO) and serum-free media and subsequently stained with rhodamine 123 (1 mM; 1 μL; Sigma-Aldrich) for 30 min at 37 °C in the dark.[30–32] After washing with PBS (pH 7.4) and serum-free media, the cells were stained with Hoechst 33342 (2.5 mM; 1 μL; Thermo Fisher Scientific) for 15 min at 37 °C in the dark.[30,33,34] Fluorescent responses of rhodamine 123 and Hoechst 33342 were detected by an EVOS FL fluorescence microscope (Advanced Microscopy Group, Bothell, WA, USA) with a GFP light cube [excitation at 471 (± 11) nm and emission at 510 (± 21) nm] and a DAPI light cube [excitation at 357 (± 22) nm and emission at 447 (± 30) nm], respectively. At least ten randomly selected fields per condition were captured with a microscope, and the fluorescence intensity was analyzed by the ImageJ software (National Institutes of Health, Bethesda, MD, USA). Data are presented as mean ± s.e.m. for n = 22–138 cells. For the statistical analysis, Student's *t*-test was used.

Table S1. Abs and EPR spectroscopic features of Cu(II)-containing enzymes and synthetic complexes.

Copper(II) Complexes		Ligands	Electronic Transitions λ / nm (ε / $M^{-1}cm^{-1}$)			EPR Parameters	
			N(His) → Cu CT	S(σ) → Cu CT	S(π) → Cu CT	$g_{\parallel}$	$A_{\parallel}$ (MHz)
**Type I**	Cu(azurin)[35]		460 (580)	567 (504)	631 (3798)	2.260	180
	Cu(plastocyanin)[36]		468 (288)	535 (600)	599 (5160)	2.226	189
	Cu(plantacyanin)[37]	2His, Cys, Met	444 (1610)	476 (1320)	585 (3410)	2.207	171
	Cu(nitrite reductase)[38,39]		–	390 (2210)<br>457 (2590)	570 (1490)	2.195	219
**Type II**	Cu(SOD*)[40]	4His, H_2O	256 (3300)	–	–	2.257	396
	Cu(galactose oxidase)[41]	2His, 2Tyr, Acetate	314 (1370)	–	–	2.280	525
	Cu(quercetinase)[42]	3His, H_2O	–	–	–	2.330	447
	Cu(nitrosocyanin)[43]	2His, Cys, Glu, H_2O	–	391 (7000)	491 (2200)	2.245	440
	Cu(DJ-1*)[44]	Cys, Glu, H_2O	–	370 (3300)	–	2.220	559
**Synthetic Complexes**	Cu(tetraazomercaptide)[45]	RS^-, $4NR_2$	–	360 (2200)	418 (1000)	2.117	–
	Cu(EDDA*)[46]	$2RO^-$, $2SR_2$	–	325 (2600)	–	–	–
	Cu(AMPAMBT*)[47]	$2NR_2$, RS^-, SR_2	–	345 (5470)<br>391 (3500)	510 (800)	2.230	–
This Work Cu(II)–M-TTR	*Site A*	–	–	377 (3600)	–	2.225	480
	Site B	–	–	–	–	2.270	510

Systems with sulfur coordination display S-to-Cu CT transitions in the visible region, while N- or O-based ligands alone cannot engender prominent Abs features at energy below 350 nm. With the sulfur coordination, type I copper enzymes with Cys, Met, and 2 His ligations can adopt distorted tetrahedral structures to exhibit unique EPR hyperfine values of $A_{||}$ < *ca*. 200 MHz, while type II red copper enzymes display "normal" EPR values of $A_{||}$ > *ca*. 400 MHz with square planar or pyramidal geometry. The Abs and EPR features of site A in M-TTR indicate that the Cu(II) center is likely ligated by Cys and/or Met ligands with the "normal" geometry, while site B would not contain S-based ligands. These analyses were used for constructing DFT models in Figures S7–S15.

*SOD, Superoxide dismutase; DJ-1, Parkinson disease protein 7; EDDA, (ethylenedithio)diacetate; AMPAMBT, 2-[[[1-(aminomethyl)-3-(methylthio)propyl]amino]methyl]benzenethiolato.

Table S2. DFT-calculated EPR parameters of Cu(II)–M-TTR models in Figures S7–S13.

		Models		g_x	g_y	g_z	A_z (MHz)
**Exp.**		**Cu(II)–M-TTR, Site A**		**2.017**	**2.075**	**2.225**	**480**
DFT	Site 1 (Cys, His, H_2O)	Model #1-A	Cu(II)(Cys-S^-)(His)$(H_2O)_2$	2.039	2.056	2.164	230
		Model #1-B	Cu(II)(Cys-S^-)(His)$(H_2O)_3$	2.048	2.056	2.166	330
		**Model #1-C**	**Cu(II)(Cys-S^-)(His)(H_2O)(OH)**	**2.024**	**2.069**	**2.161**	**463**
		Model #1-D	Cu(II)(Cys-SH)(His)$(H_2O)_2$	2.042	2.062	2.176	481
		Model #1-E	Cu(II)(Cys-SH)(His)$(H_2O)_3$	2.056	2.059	2.188	433
		Model #1-F	Cu(II)(Cys-SH)(His)(H_2O)(OH)	2.03	2.068	2.157	513
	Site 1 (Cys, Met, His, H_2O)	Model #1-G	Cu(II)(Cys-S^-)(Met)(His)(H_2O)	2.024	2.073	2.16	314
		Model #1-H	Cu(II)(Cys-S^-)(Met)(His)(OH)	2.014	2.093	2.161	352
		Model #1-I	Cu(II)(Cys-SH)(Met)(His)(H_2O)	2.041	2.046	2.156	442
		**Model #1-J**	**Cu(II)(Cys-SH)(Met)(His)(OH)**	**2.027**	**2.053**	**2.149**	**452**
	Site 1 (Met, His, H_2O)	Model #1-K	Cu(II)(Met)(His)$(H_2O)_2$-(Cys-S^-)	2.036	2.045	2.147	573
		Model #1-L	Cu(II)(Met)(His)(H_2O)(OH)-(Cys-S^-)	2.037	2.039	2.142	581
		Model #1-M	Cu(II)(Met)(His)$(H_2O)_3$-(Cys-SH)	2.024	2.082	2.167	494
		Model #1-N	Cu(II)(Met)(His)$(H_2O)_2$(OH)-(Cys-SH)	2.009	2.099	2.15	482
		Model #1-O	Cu(II)(Met)(His)$(H_2O)_3$(OH)-(Cys-SH)	2.017	2.097	2.16	437
**Exp.**		**Cu(II)–M-TTR, Site B**		2.030	2.065	2.270	510
DFT	Site 2	Model #2-A	Cu(II)$(His)_2$(Glu)$(H_2O)_2$	2.026	2.095	2.192	471
		Model #2-B	Cu(II)$(His)_2$(Glu)(H_2O)(OH)	2.022	2.087	2.17	370
	Site 3	Model #3-C	Cu(II)(His)(Glu)(Asp)(H_2O)	2.065	2.08	2.222	474
		Model #3-D	Cu(II)(His)(Glu)(Asp)$(H_2O)_2$	2.033	2.105	2.205	484

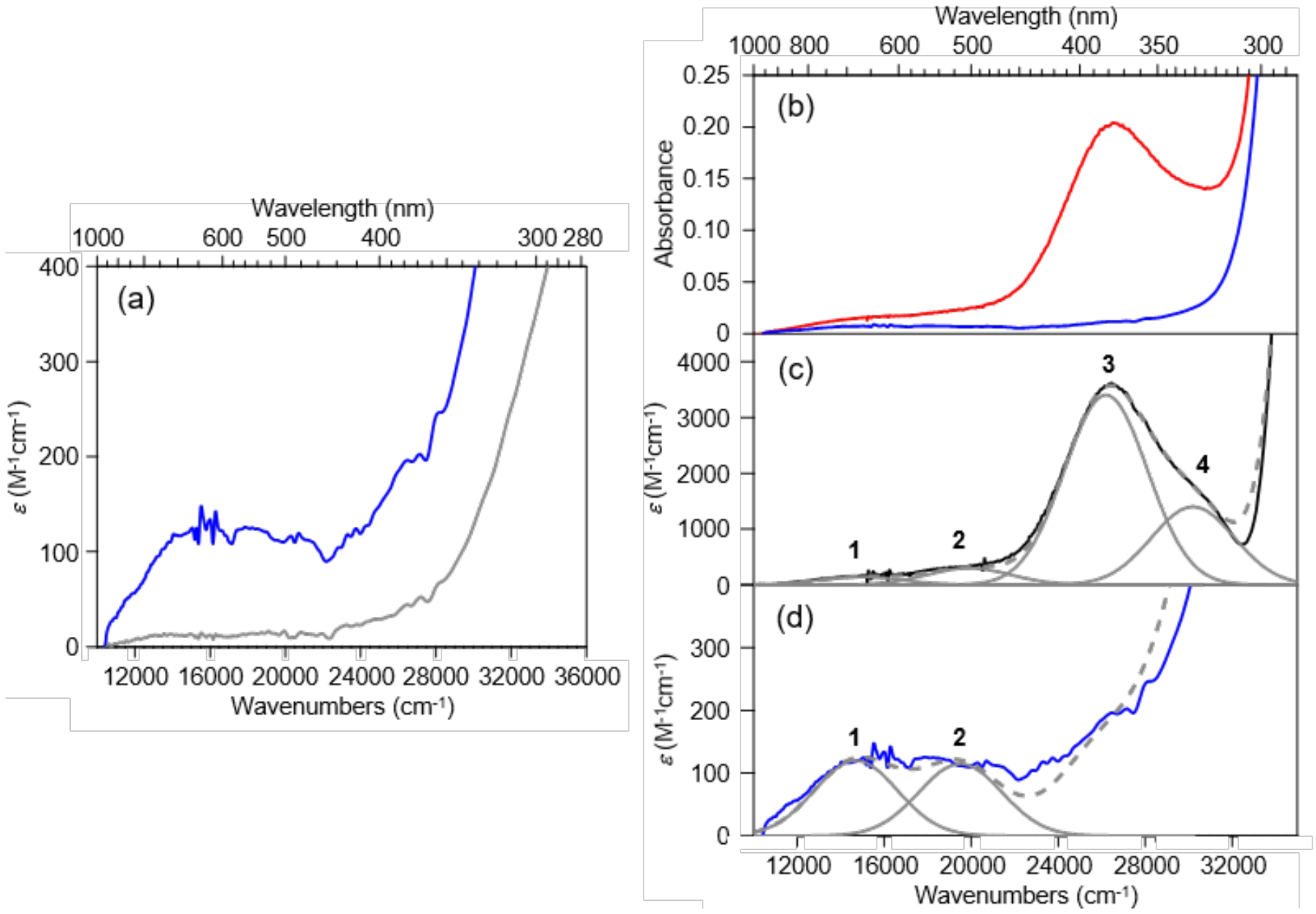

#	Position (cm^{-1})	ε($M^{-1}cm^{-1}$)
1	14900	114
2	19900	302
3	26200	3400
4	30200	1400

#	Position (cm^{-1})	ε($M^{-1}cm^{-1}$)
1	14700	120
2	19500	115

Figure S1. Analysis of $CuCl_2$ treated with M-TTR by Abs spectroscopy. (a) Abs spectra of $CuCl_2$ incubated with (blue) and without (gray) M-TTR for 24 h. This comparison highlights that the emergence of ligand-field transitions at 500–1,000 nm can be attributed to Cu(II) bound to M-TTR, specifically site B, as protein-free Cu(II) species do not exhibit Abs features at this low concentration of 100 μM. Conditions: [M-TTR] = 100 μM; [$CuCl_2$] = 100 μM; 20 mM HEPES, pH 7.4, 150 mM NaCl. The measurements were performed in triplicate. (b) Overlay of the Abs spectrum obtained upon mixing the solutions of M-TTR and $CuCl_2$ (red) and the 60%-scaled Abs spectrum of site B (blue). (c) Abs spectrum of site A only (black solid) obtained by subtracting the latter from the former in (b). The spectrum of site A contains at least two *d-d* transitions, bands 1 and 2, and two CT transitions, bands 3 and 4. (d) Abs spectrum of site B (blue) exhibiting at least two *d-d* transitions, bands 1 and 2. The Abs feature growing at energy above 24,000 cm^{-1} could not be specified as its band maximum is obscured by the Abs of M-TTR. For the Gaussian deconvolution, the bandwidth of 2,700 cm^{-1} was used. Each transition is represented by a Gaussian band (gray solid) and their sum spectra are given in dashed gray.

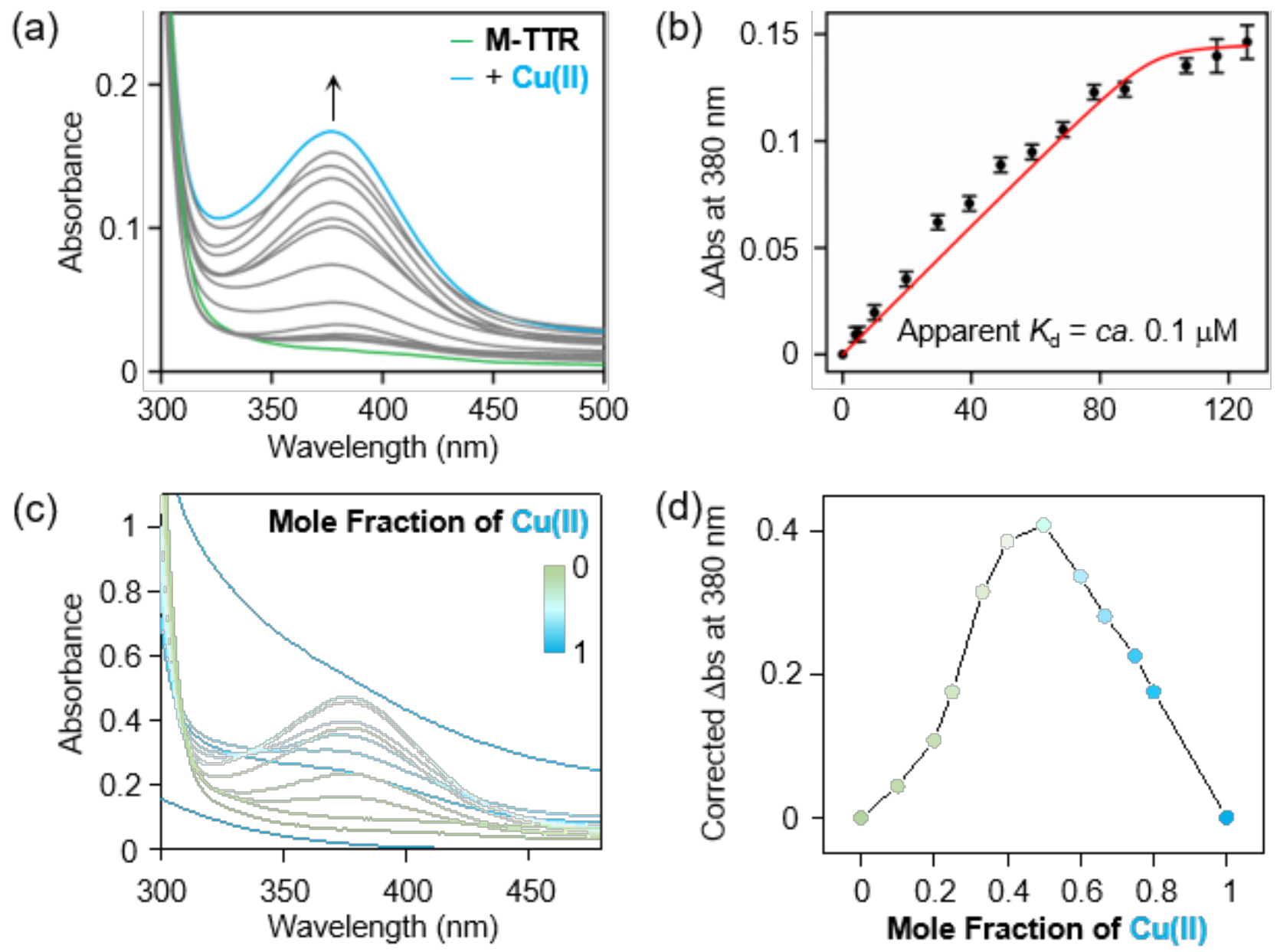

Figure S2. Cu(II) binding to M-TTR. (a) Titration experiments performed by Abs spectroscopy to monitor the Abs change of M-TTR at 380 nm upon addition of various concentrations of Cu(II). Conditions: [M-TTR] = 100 μM; [$CuCl_2$] = 4–126 μM; 20 mM HEPES, pH 7.4, 150 mM NaCl; room temperature. (b) Alteration of Abs at 380 nm as a function of [Cu(II)]. The data were fitted to the red curve to estimate the apparent binding affinity. The titration profile suggests Cu(II) binding in the submicromolar range under these experimental conditions. Data are presented as mean ± s.e.m. for n = 3. (c) Abs spectra of M-TTR with Cu(II) at varying Cu(II) mole fractions. Conditions: [M-TTR] = 0–450 μM; [$CuCl_2$] = 0–450 μM; 20 mM HEPES, pH 7.4, 150 mM NaCl; room temperature. (d) Job plot analysis based on the Abs at 380 nm from the spectra shown in (c).

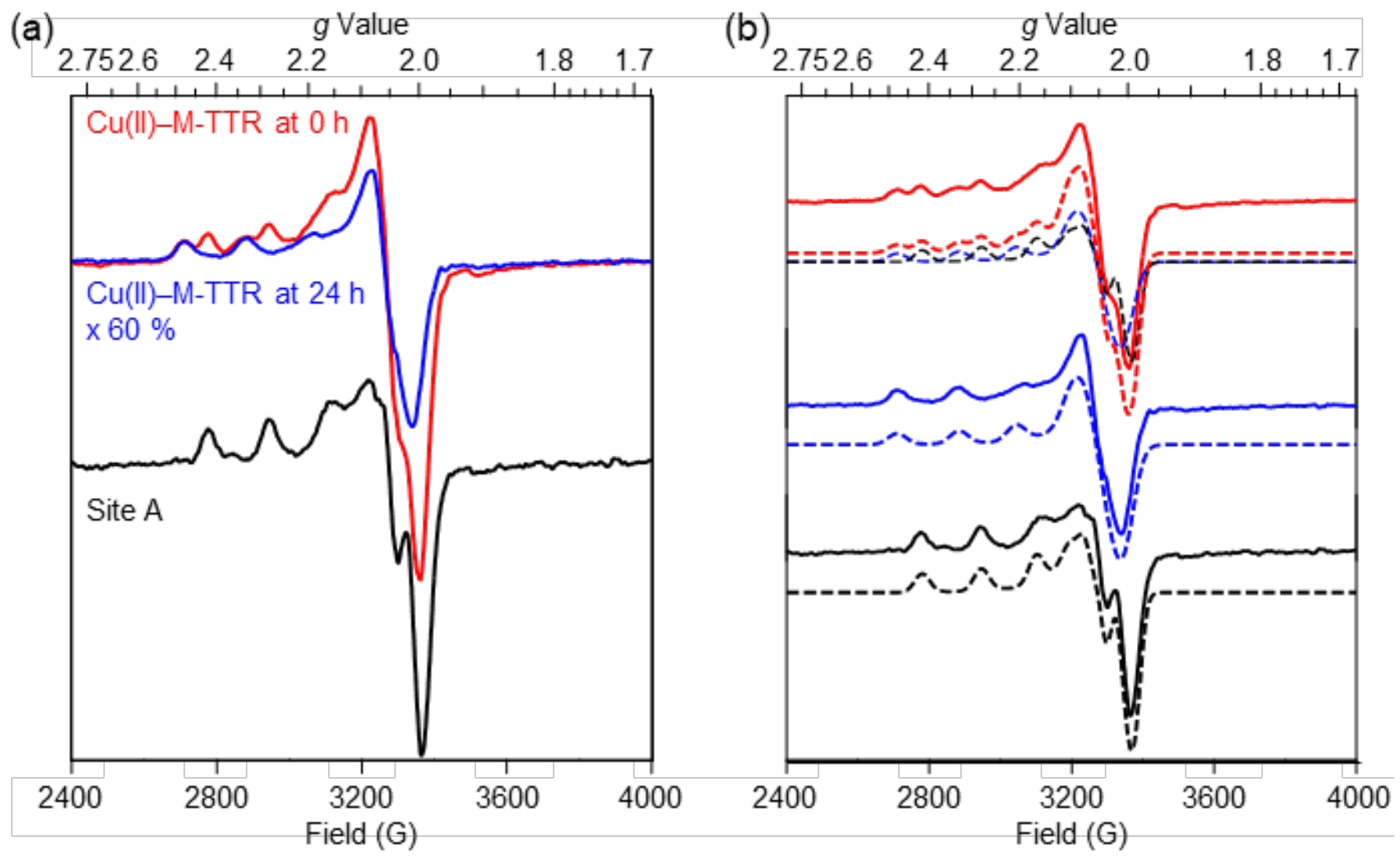

Figure S3. EPR spectra decomposition. (a) The EPR spectrum of a sample obtained by freezing the solution containing both M-TTR and $CuCl_2$ right after mixing (red) is well overlapped with 60 % of that of a sample prepared by freezing the solution mixture after 24 h incubation (blue). By subtracting the latter from the former, the EPR spectrum of site A alone (black) was gained. (b) Experimental (solid) and simulated (dashed) EPR spectra of site A (black) and site B (blue). For simulations, EPR parameters of g_x = 2.017, g_y = 2.075, g_z = 2.225, A_x = 0 MHz, A_y = 50 MHz, and A_z = 480 MHz for site A and g_x = 2.030, g_y = 2.065, g_z = 2.270, A_x = 0 MHz, A_y = 0 MHz, and A_z = 510 MHz for site B were utilized, while the 0-h spectrum (red) could be reconstructed by adding the simulated spectra of 40% site A and 60% site B.

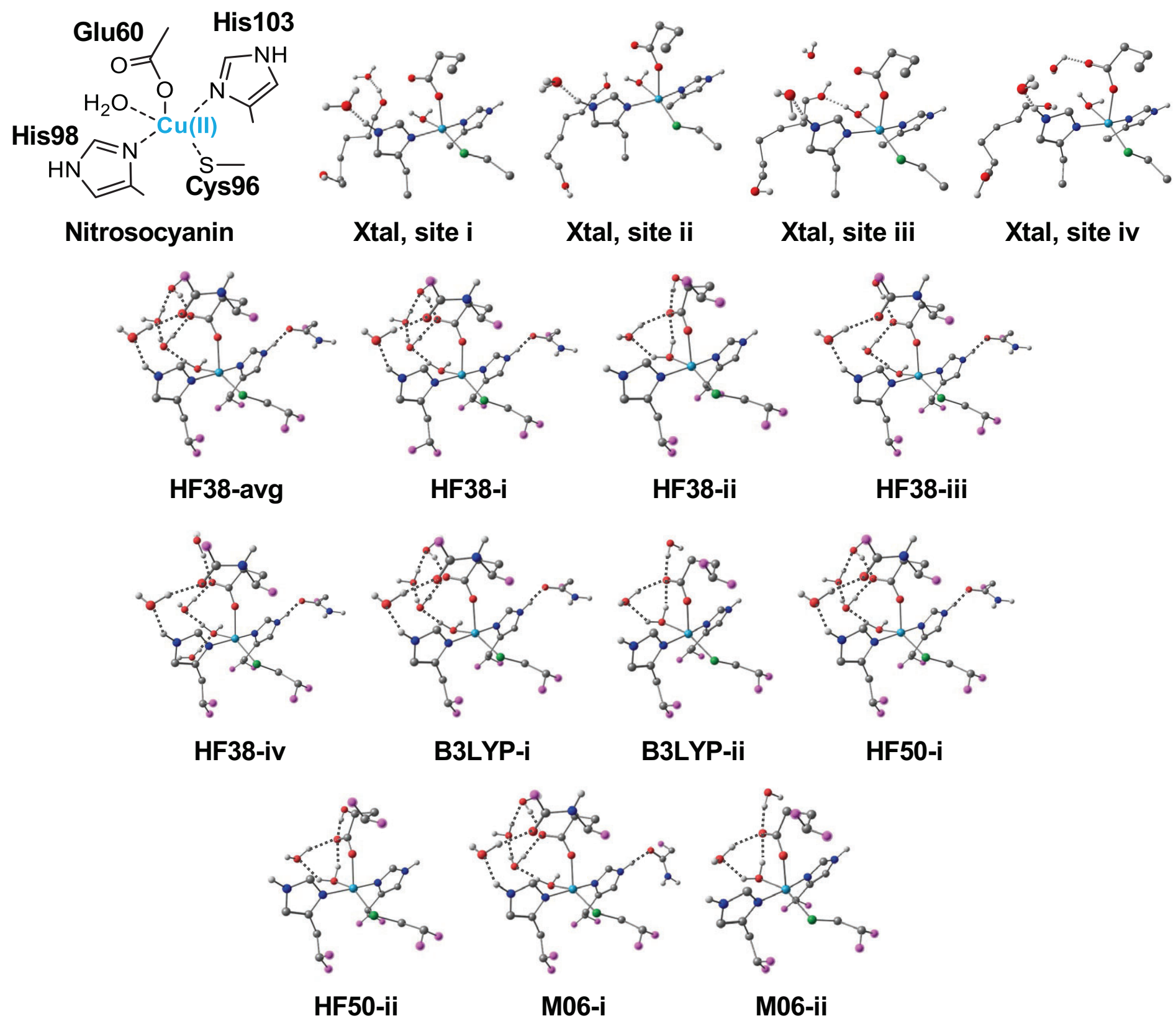

Copper Sites in Nitrosocyanin	Exp. (Crystal)				
	i	ii	iii	iv	Average
**Cu-S(Cys) / Å**	2.260	2.291	2.251	2.274	2.26
**Cu-O(Glu) / Å**	2.053	2.105	2.097	2.106	2.09
**Cu-O(H_2O) / Å**	2.059	2.415	2.381	2.145	2.25
**Cu-N(His98) / Å**	2.006	2.057	1.981	2.019	2.02
**Cu-N(His103) / Å**	1.981	1.974	1.983	1.951	1.97

DFT Models	HF38-avg	HF38-i	HF38-ii	HF38-iii	HF38-iv
**Cu-S(Cys) / Å**	2.260	2.377	2.324	2.364	2.362
**Cu-O(Glu) / Å**	2.202	2.150	2.089	2.164	2.135
**Cu-O(H_2O) / Å**	2.059	2.126	2.149	2.121	2.212
**Cu-N(His98) / Å**	2.006	1.959	1.985	1.961	1.950
**Cu-N(His103) / Å**	1.981	1.953	1.970	1.954	1.946

DFT models	B3LYP-i	B3LYP-ii	HF50-i	HF50-ii	M06-i	M06-ii
**Cu-S(Cys) / Å**	2.404	2.354	2.383	2.326	2.353	2.300
**Cu-O(Glu) / Å**	2.184	2.096	2.127	2.075	2.137	2.076
**Cu-O(H_2O) / Å**	2.189	2.268	2.121	2.138	2.187	2.212
**Cu-N(His98) / Å**	1.970	1.993	1.963	1.991	1.940	1.976
**Cu-N(His103) / Å**	1.960	1.974	1.959	1.977	1.938	1.960

Figure S4. DFT models for the Cu(II) sites of nitrosocyanin. Four Cu(II)-binding sites (i–iv) obtained from the X-ray crystal structure of nitrosocyanin (PDB 1IBY[7]). As constraining backbone atoms highlighted in purple, the truncated models for sites (i–iv) were optimized using the BP86 functional mixed with 38% HF exchange. The resulting structures, HF38-i–HF38-iv, overestimated Cu–S bond lengths. Thus, an additional model, HF38-avg, was prepared by constraining the Cu–S bond length at 2.26 Å, the average distance obtained from the four sites of the crystal structure. Sites (i) and (ii), with the shortest and longest Cu–S bond lengths, were further examined with the B3LYP, BP86 with 50% HF exchange (HF50), and M06 functionals. Their TDDFT-simulated Abs spectra are given in Figure S5. Cyan, green, gray, blue, red, and white balls indicate copper, sulfur, carbon, nitrogen, oxygen, and hydrogen atoms, respectively.

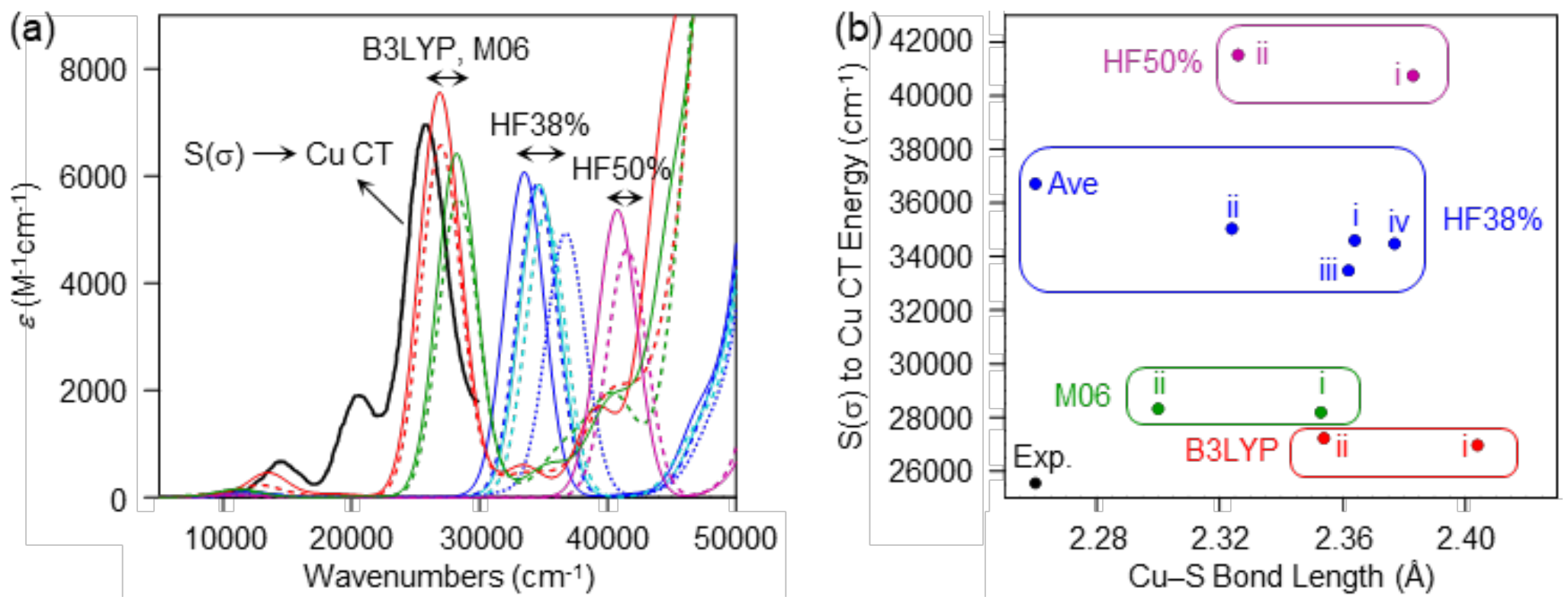

Figure S5. TDDFT calculations of the nitrosocyanin models. (a) TDDFT-simulated Abs spectra of models HF38-avg (solid blue), HF38-i (solid cyan), HF38-ii (dashed cyan), HF38-iii (dashed blue), HF38-iv (dotted blue), B3LYP-i (solid red), B3LYP-ii (dashed red), HF50-i (solid magenta), HF50-ii (dashed magenta), M06-i (solid green) and M06-ii (dashed green). The experimental Abs spectrum of nitrosocyanin (black) was reconstructed from reference[43] using the WebPlotDigitizer 4.6 program[48]. (b) Plot between the S(σ) → Cu CT energy and the Cu–S bond length of the nitrosocyanin DFT models. Unexpectedly no significant correlation was found between the CT energy and the bond length, while the plot emphasizes that the functional determines the calculated S(σ) → Cu CT energy. As model M06-ii best reproduced the experimental values, the M06 functional was selected for screening M-TTR models.

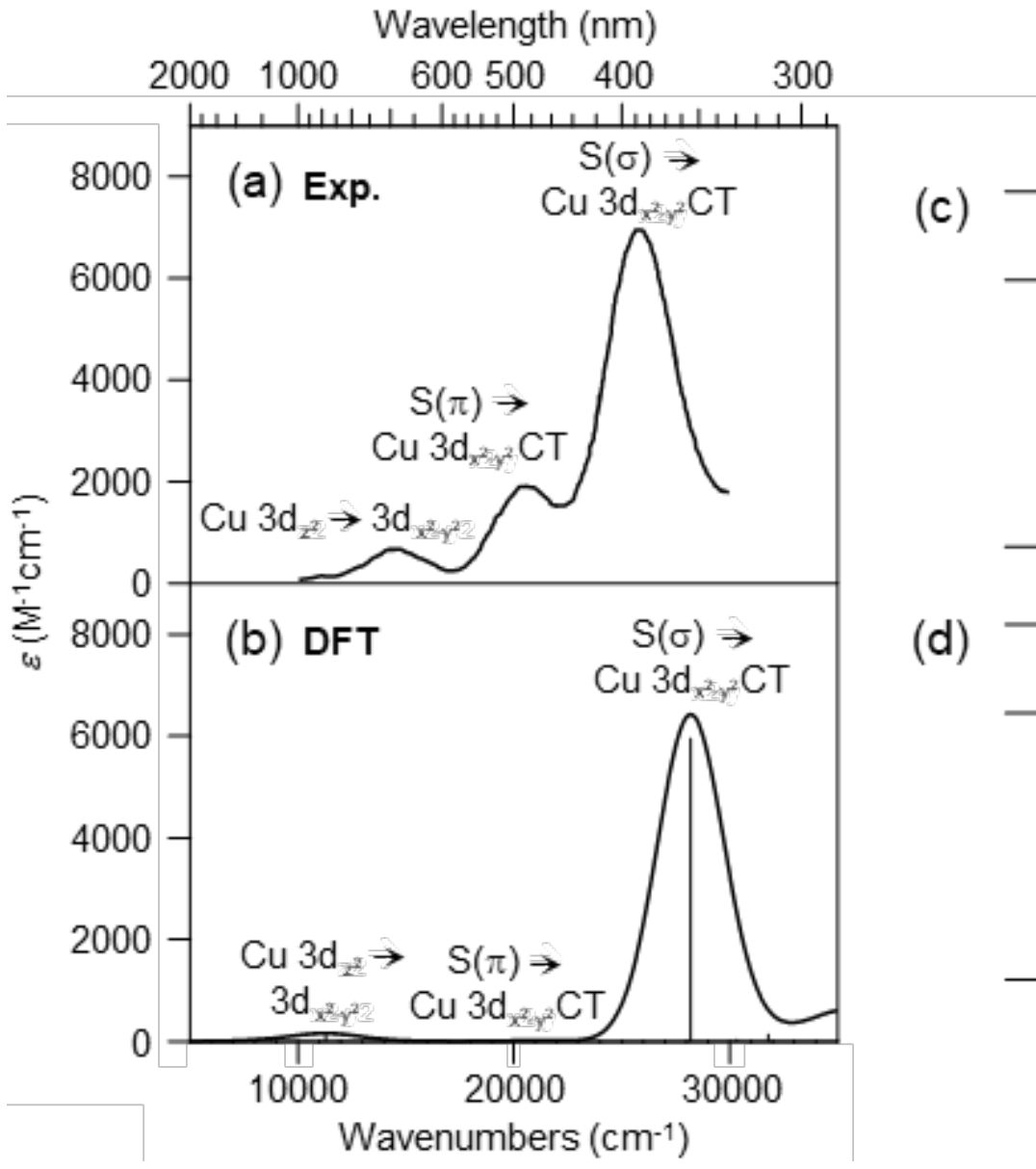

(c)

Band	E, cm^{-1} (Exp.)	E, cm^{-1} (DFT)	Δ (DFT-Exp.)
**Cu $3d_{z^2}$**	15000	11286	–3714
**Cys-S(π)**	20350	20892	+542
**Cys-S(σ)**	25550	28183	+2633

(d)

EPR	g_x	g_y	g_z	A_z (MHz)
**Exp.**	2.036	2.059	2.245	440
**DFT**	2.022	2.072	2.147	468
**Δ (DFT-Exp.)**	–0.014	+0.013	–0.098	+28

Figure S6. Calibration of DFT-calculated spectroscopic properties. (a) Experimental[43] and (b) M06-simulated Abs spectra of nitrosocyanin. (c) Comparison between the experimental and DFT-calculated d-d and CT transition energies. (d) Comparison between the experimental and B3LYP-calculated EPR parameters of nitrosocyanin. The overestimation of the S(σ) → Cu CT energy by 2,600 cm^{-1} and the underestimation of the g_z value were considered for further calculations of M-TTR.

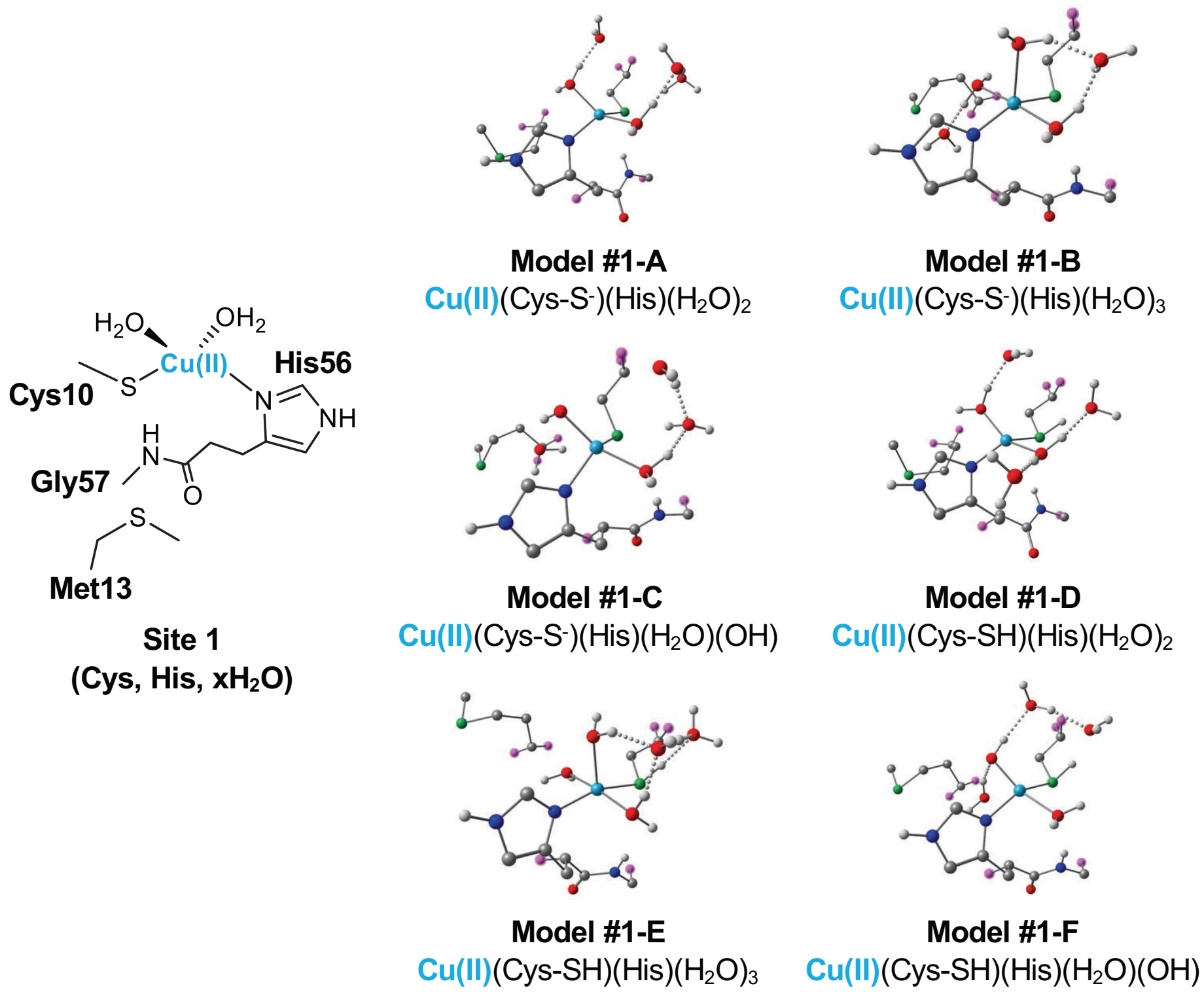

Figure S7. DFT models for Cu(II) binding to site 1 in M-TTR without Met13 ligation. The ligation of Cys10, His56, and two water molecules was considered with variations in the protonation level of the Cys and water ligands. For the better visualization, hydrogens attached to carbons are omitted. Cyan, green, gray, blue, red, and white balls indicate copper, sulfur, carbon, nitrogen, oxygen, and hydrogen atoms, respectively. Atoms constrained during geometry optimization are highlighted in purple.

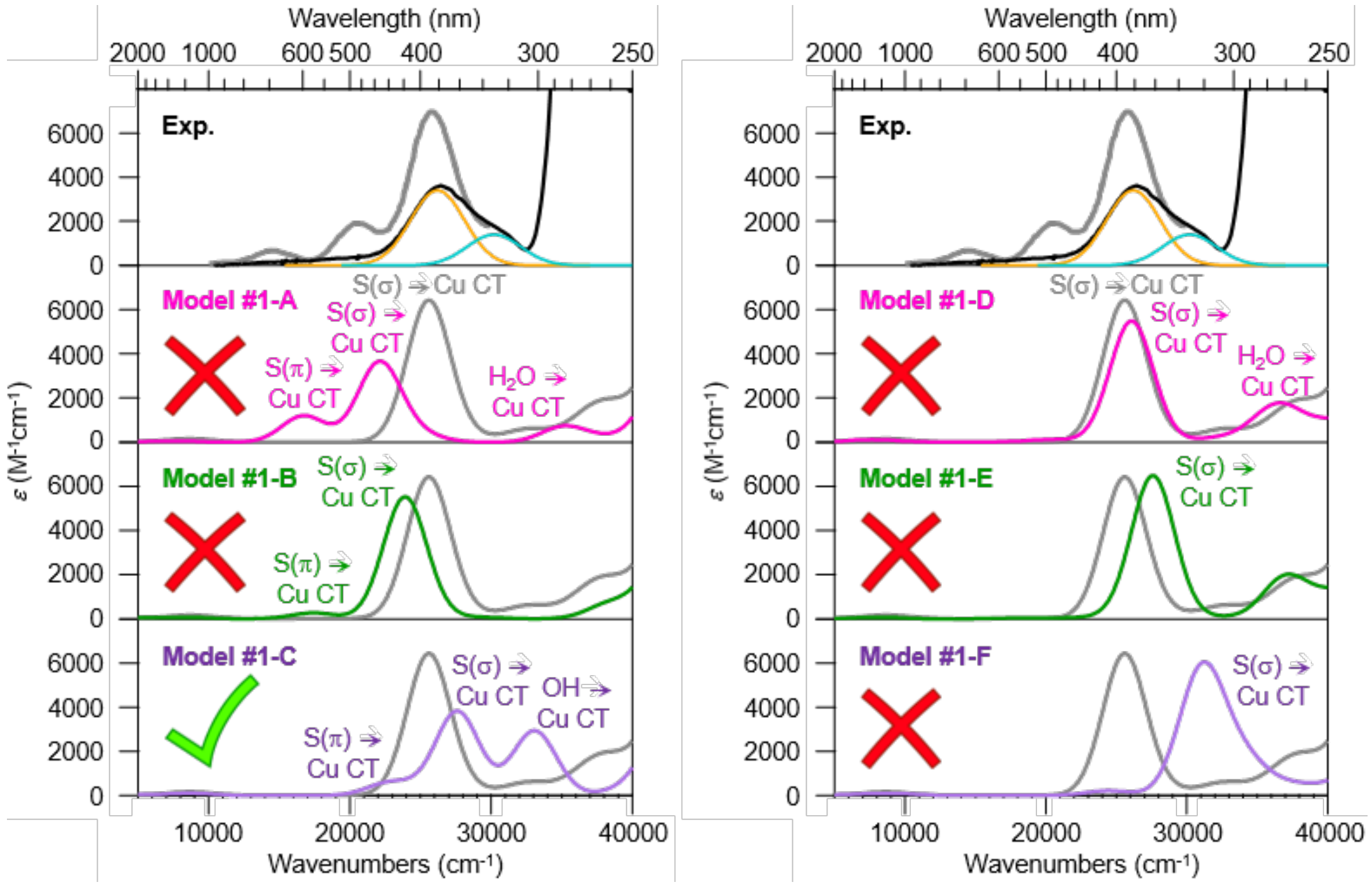

Figure S8. TDDFT-simulated Abs spectra of the site 1 models without Met13 coordination. The experimental Abs spectrum of M-TTR site A (black) and its Gaussian deconvolution bands for the CT features (orange and cyan) are given in the top panel. The TDDFT-simulated Abs spectra of the site 1 models given in Figure S7 are shown in color, while the experimental and TDDFT spectra of nitrosocyanin are presented in gray as an energy calibration standard. Using the nitrosocyanin-based calibration, all TDDFT-calculated Abs spectra were down-shifted by 2,600 cm^{-1}. A suitable model should be capable of reproducing two CT bands, one with its energy that matches the band maximum of nitrosocyanin, and the other with a higher energy and a lower intensity than the former. Models #1-B and #1-F exhibit a single CT band and thus could be excluded. Models #1-A, #1-D, and #1-E display two CT bands, which are too apart in energy to reproduce the data. Model #1-C effectively reproduces the experimental Abs features, suggesting it to be a proper candidate for site A.

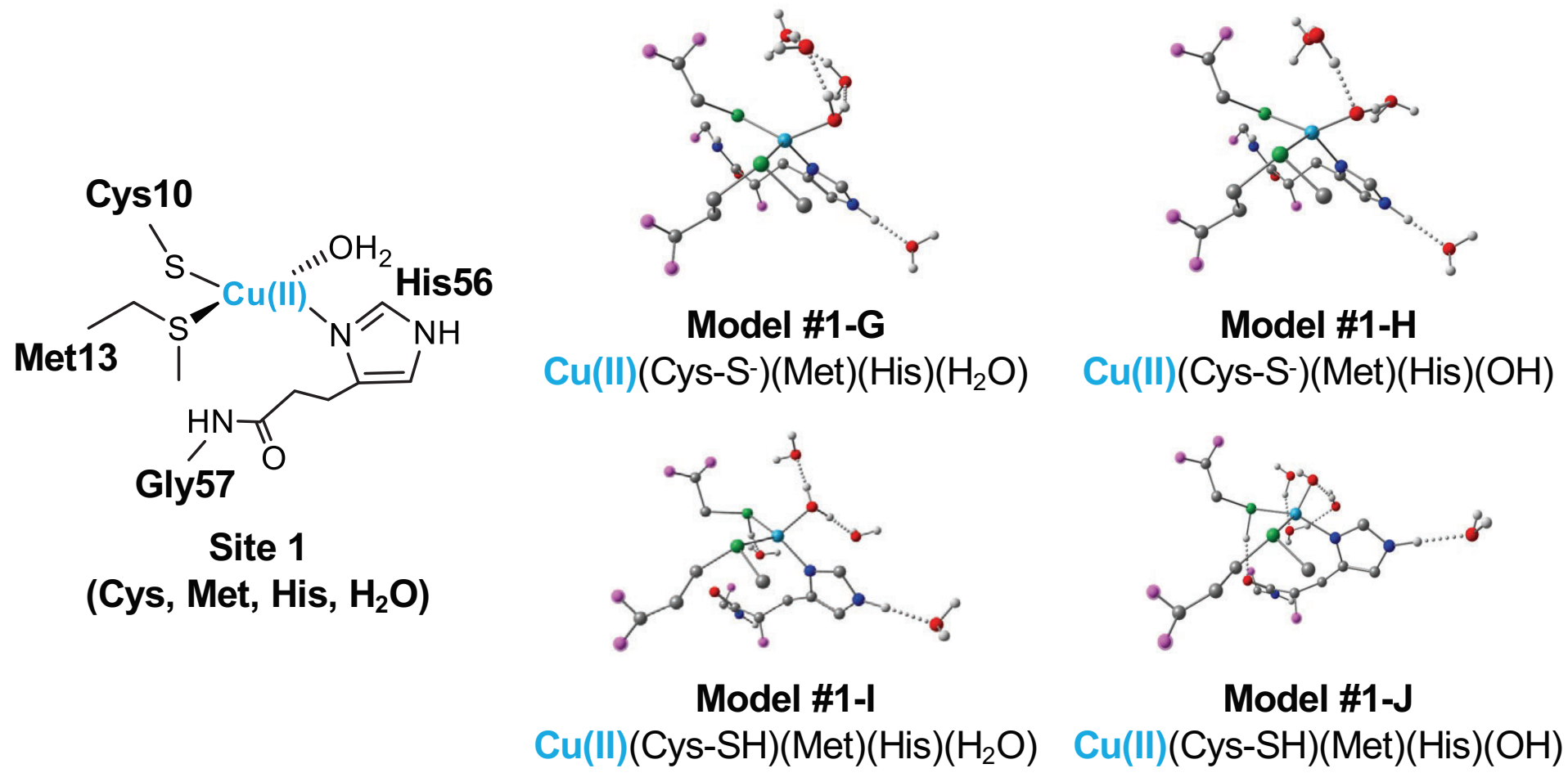

Figure S9. DFT models for Cu(II) binding to site 1 in M-TTR with Met13 coordination. The ligation of Cys10, Met13, His56, and a water molecule was considered with variations in the protonation level of the Cys and water ligands. For the better visualization, hydrogens attached to carbons are omitted. Cyan, green, gray, blue, red, and white balls indicate copper, sulfur, carbon, nitrogen, oxygen, and hydrogen atoms, respectively. Atoms constrained during geometry optimization are highlighted in purple.

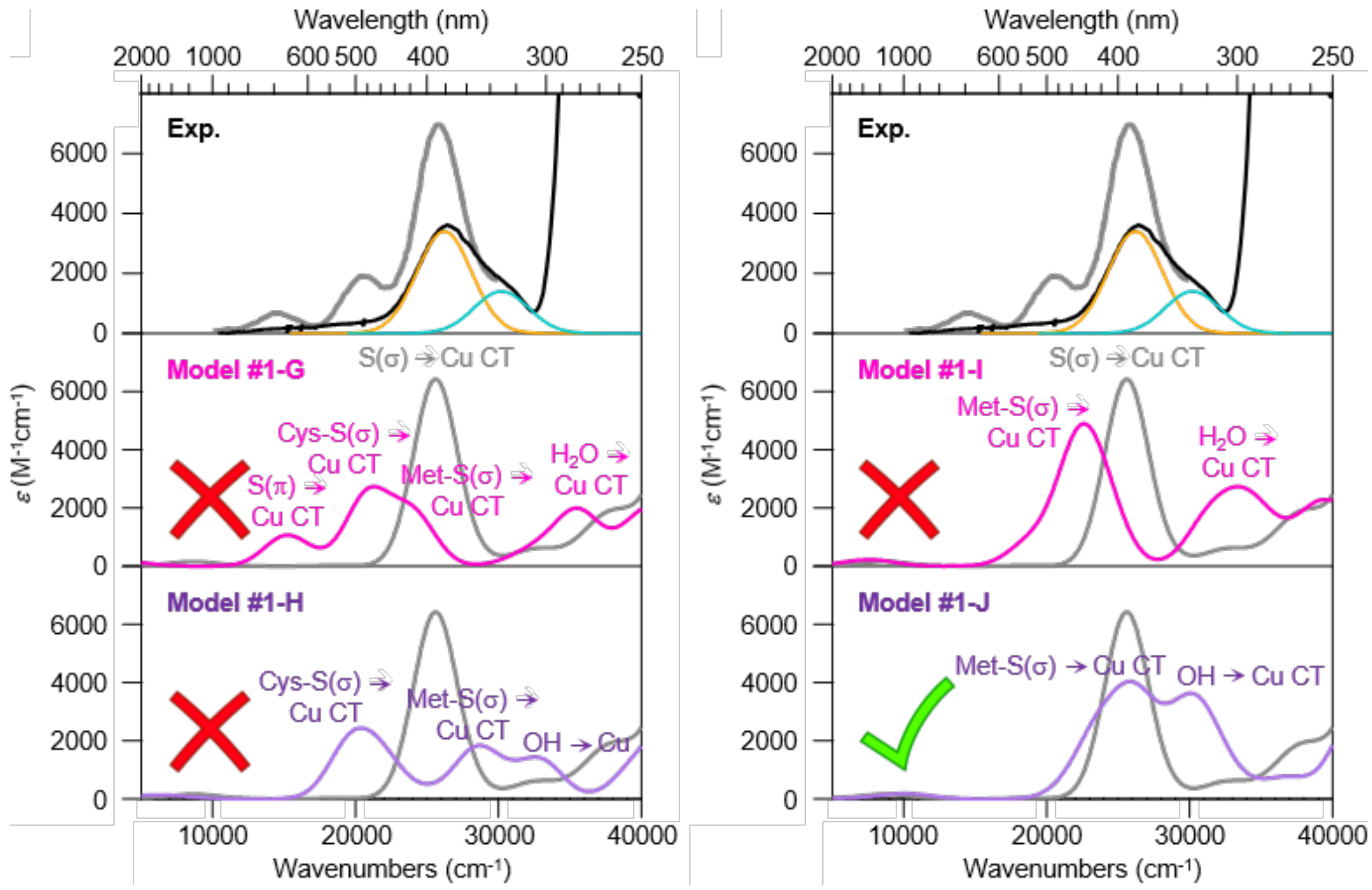

Figure S10. TDDFT-simulated Abs spectra of the site 1 models with Met13 coordination. The experimental Abs spectrum of M-TTR site A (black) and its Gaussian deconvolution bands for the CT features (orange and cyan) are given in the top panel. The TDDFT-simulated Abs spectra of the site 1 models given in Figure S9 are shown in color, while the experimental and TDDFT spectra of nitrosocyanin are presented in gray as an energy calibration standard. Using the nitrosocyanin-based calibration, all TDDFT-calculated Abs spectra were down-shifted by 2,600 cm^{-1}. A suitable model should be capable of reproducing two CT bands, one with its energy that matches the band maximum of nitrosocyanin, and the other with a higher energy and a lower intensity than the former. Models #1-G, #1-H, and #1-I exhibit multiple CT bands, but their energies do not align with the data. Model #1-J effectively reproduces the experimental Abs features and thus was chosen as a feasible structure for site A.

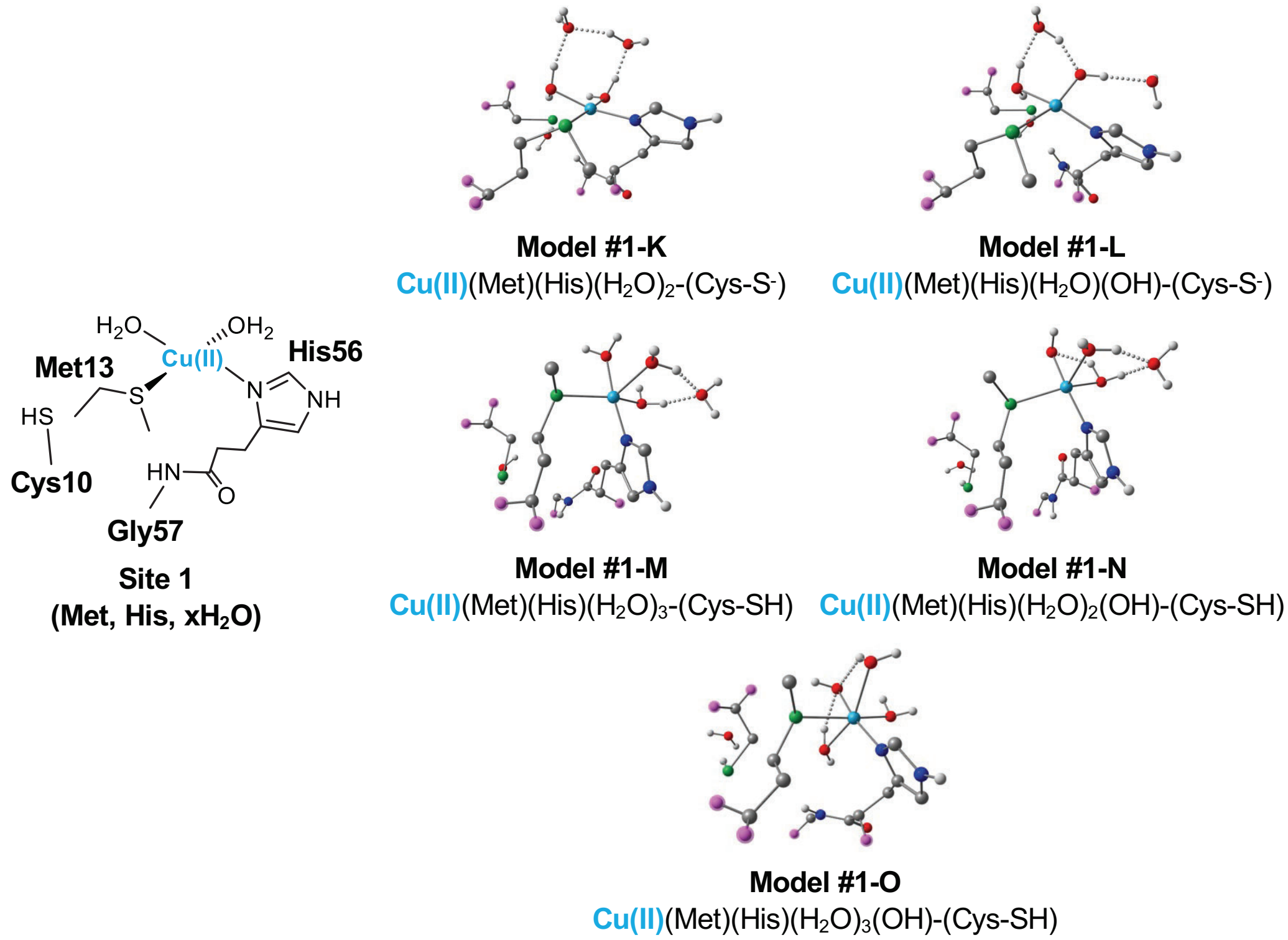

Figure S11. DFT models for Cu(II) binding to site 1 in M-TTR without Cys10 coordination. The ligation of Met13, His56, and two water molecules was considered with variations in the protonation level of the water ligands and Cys10. For the better visualization, hydrogens attached to carbons are omitted. Cyan, green, gray, blue, red, and white balls indicate copper, sulfur, carbon, nitrogen, oxygen, and hydrogen atoms, respectively. Atoms constrained during geometry optimization are highlighted in purple.

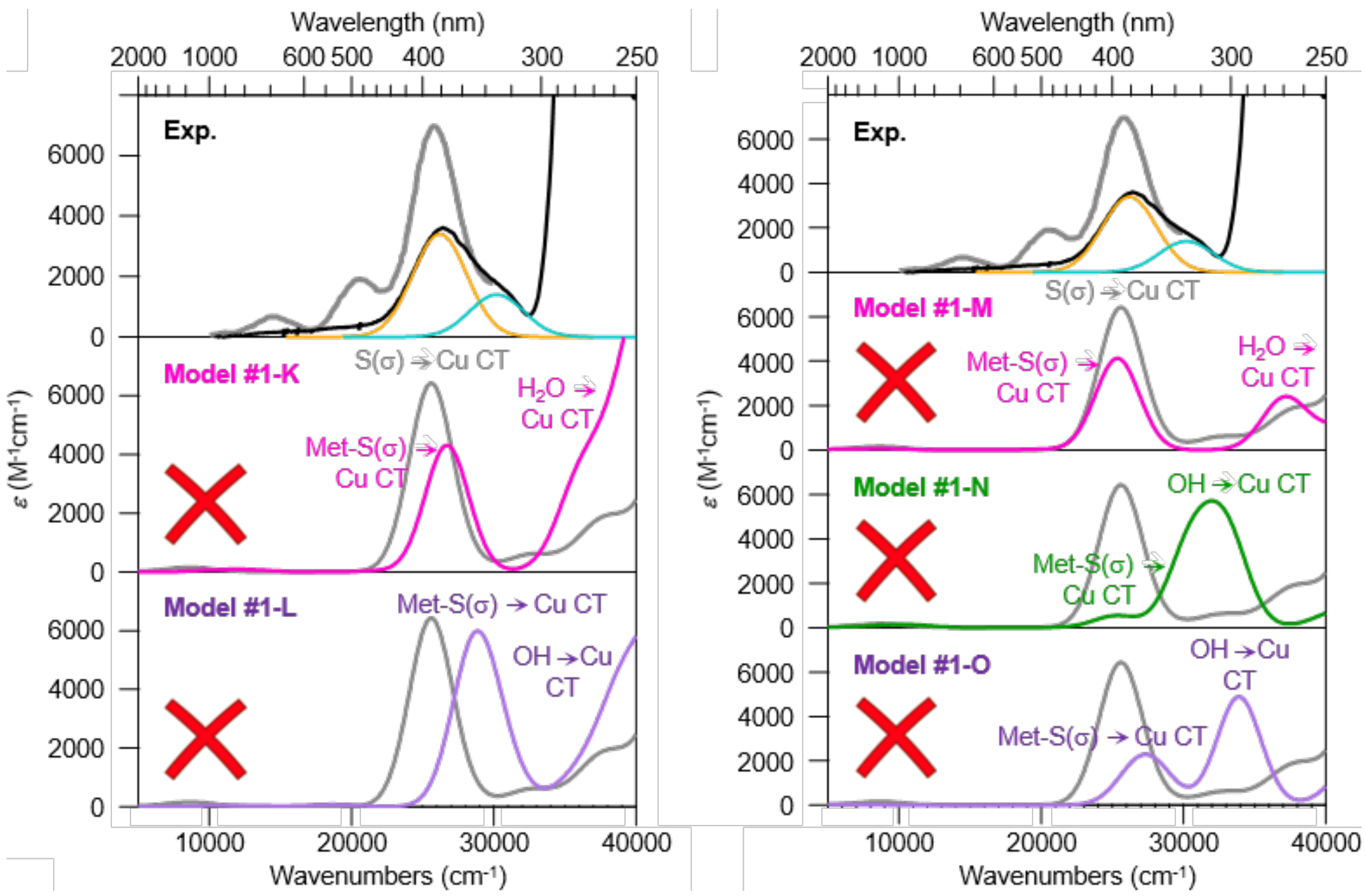

Figure S12. TDDFT-simulated Abs spectra of the site 1 models without Cys10 coordination. The experimental Abs spectrum of M-TTR site A (black) and its Gaussian deconvolution bands for the CT features (orange and cyan) are given in the top panel. The TDDFT-simulated Abs spectra of the site 1 models given in Figure S11 are shown in color, while the experimental and TDDFT spectra of nitrosocyanin are presented in gray as an energy calibration standard. Using the nitrosocyanin-based calibration, all TDDFT-calculated Abs spectra were down-shifted by 2,600 cm^{-1}. A suitable model should be capable of reproducing two CT bands, one with its energy that matches the band maximum of nitrosocyanin, and the other with a higher energy and a lower intensity than the former. Models #1-K, #1-L, #1-M, and #1-N exhibit only one major band rather than two at energy near 380 nm, and thus could be excluded. Model #1-O shows two CT bands but with inversed relative intensities and a significant energy separation, and thus was excluded.

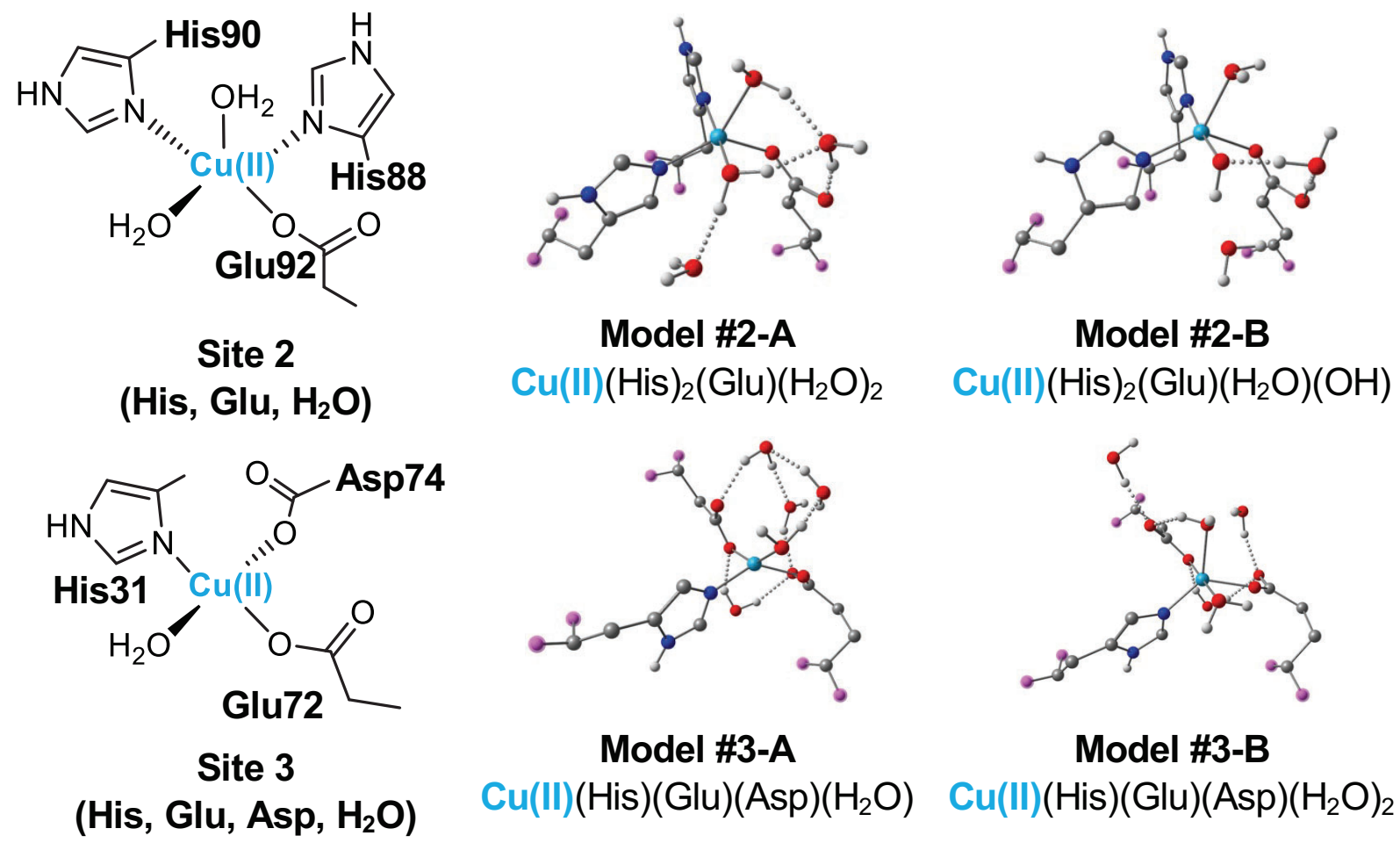

Figure S13. DFT models for Cu(II) binding to site 2 or 3 in M-TTR. The coordination of His88, His90, Glu92, and two water molecules for site 2 and the coordination of His31, Glu72, Asp74, and a water molecule for site 3 were considered with variations in the protonation level of water ligands. For the better visualization, hydrogens attached to carbons are omitted. Cyan, gray, blue, red, and white balls indicate copper, carbon, nitrogen, oxygen, and hydrogen atoms, respectively. Atoms constrained during geometry optimization are highlighted in purple.

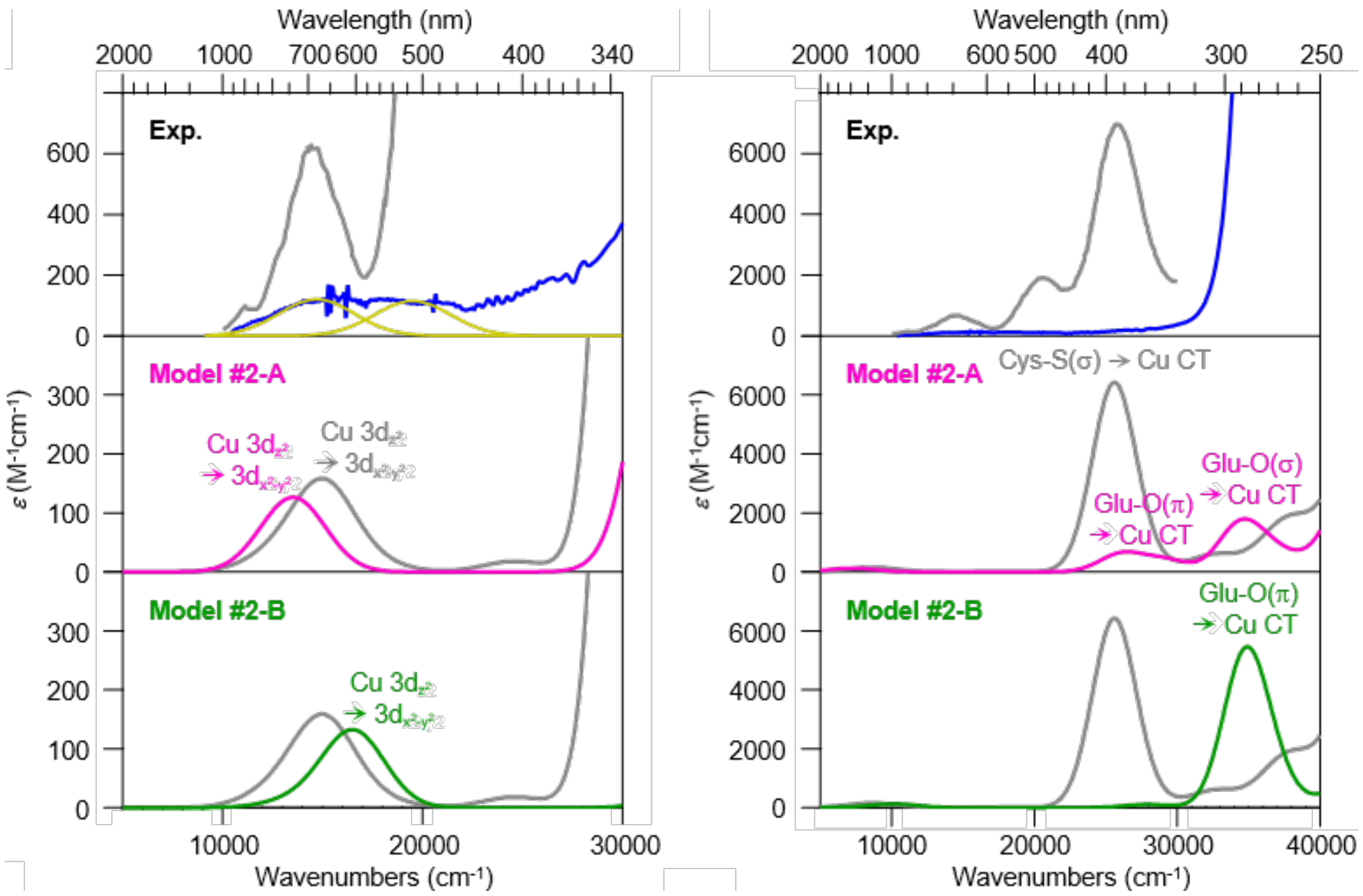

Figure S14. TDDFT-simulated Abs spectra of the site 2 models. The experimental Abs spectrum of site B (blue) with its deconvoluted *d-d* transition bands (left, yellow) are given in the top panel. For the better visualization, spectra in the near IR and visible regions are separately shown on the left and right, respectively. The TDDFT-simulated Abs spectra of the site 2 models given in Figure S13 are shown in color, while the experimental and TDDFT spectra of nitrosocyanin are given in gray as an energy calibration standard. Using the nitrosocyanin-based calibration, for the CT bands, the TDDFT-calculated Abs spectra were down-shifted by 2,600 cm^{-1} (right), while for the *d-d* transition bands, the TDDFT-calculated Abs spectra were up-shifted by 3,700 cm^{-1} (left). All models exhibit CT bands with energies exceeding 30,000 cm^{-1}, which indicates that these models are not suitable candidates for site A but can still be considered as potential candidates for site B. In experiment, site B and nitrosocyanin display *d-d* transitions at a similar energy range, and thus both models can be possible structures for site B.

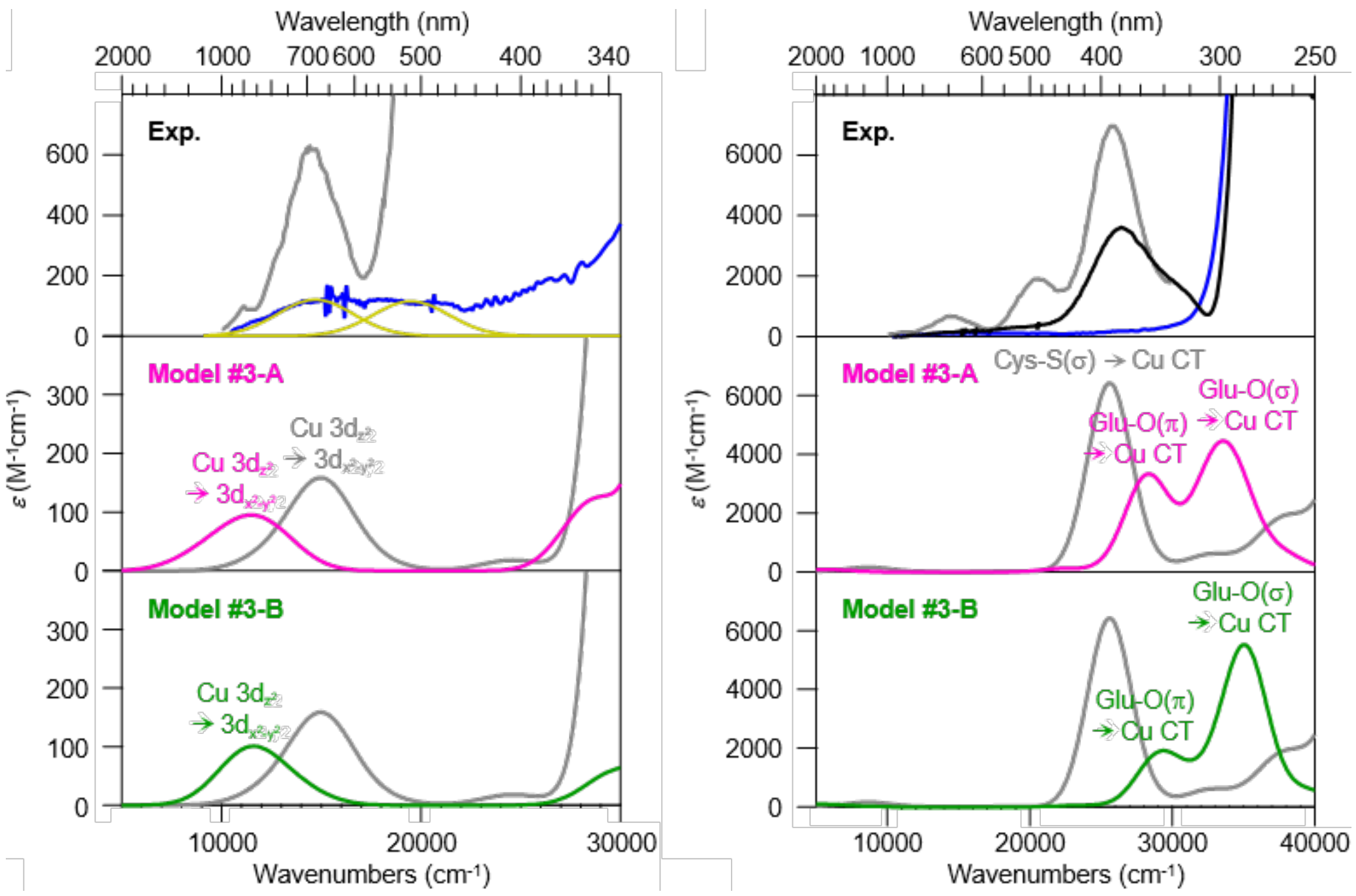

Figure S15. TDDFT-simulated Abs spectra of the site 3 models. The experimental Abs spectrum of site B (blue) with its deconvoluted *d-d* transition bands (left, yellow) are given in the top panel. For the better visualization, spectra in the near IR and visible regions are separately shown on the left and right, respectively. The TDDFT-simulated Abs spectra of the site 3 models given in Figure S13 are shown in color, while the experimental and TDDFT spectra of nitrosocyanin are given in gray as an energy calibration standard. Using the nitrosocyanin-based calibration, for the CT bands, the TDDFT-calculated Abs spectra were down-shifted by 2,600 cm^{-1} (right), while for the *d-d* transition bands, the TDDFT-calculated Abs spectra were up-shifted by 3,700 cm^{-1} (left). All models exhibit CT bands with energies exceeding 30,000 cm^{-1}, which indicates that these models are not suitable candidates for site A but can still be considered as potential candidates for site B. In experiment, site B and nitrosocyanin display *d-d* transitions at a similar energy range, and thus both models may be possible structures for site B.

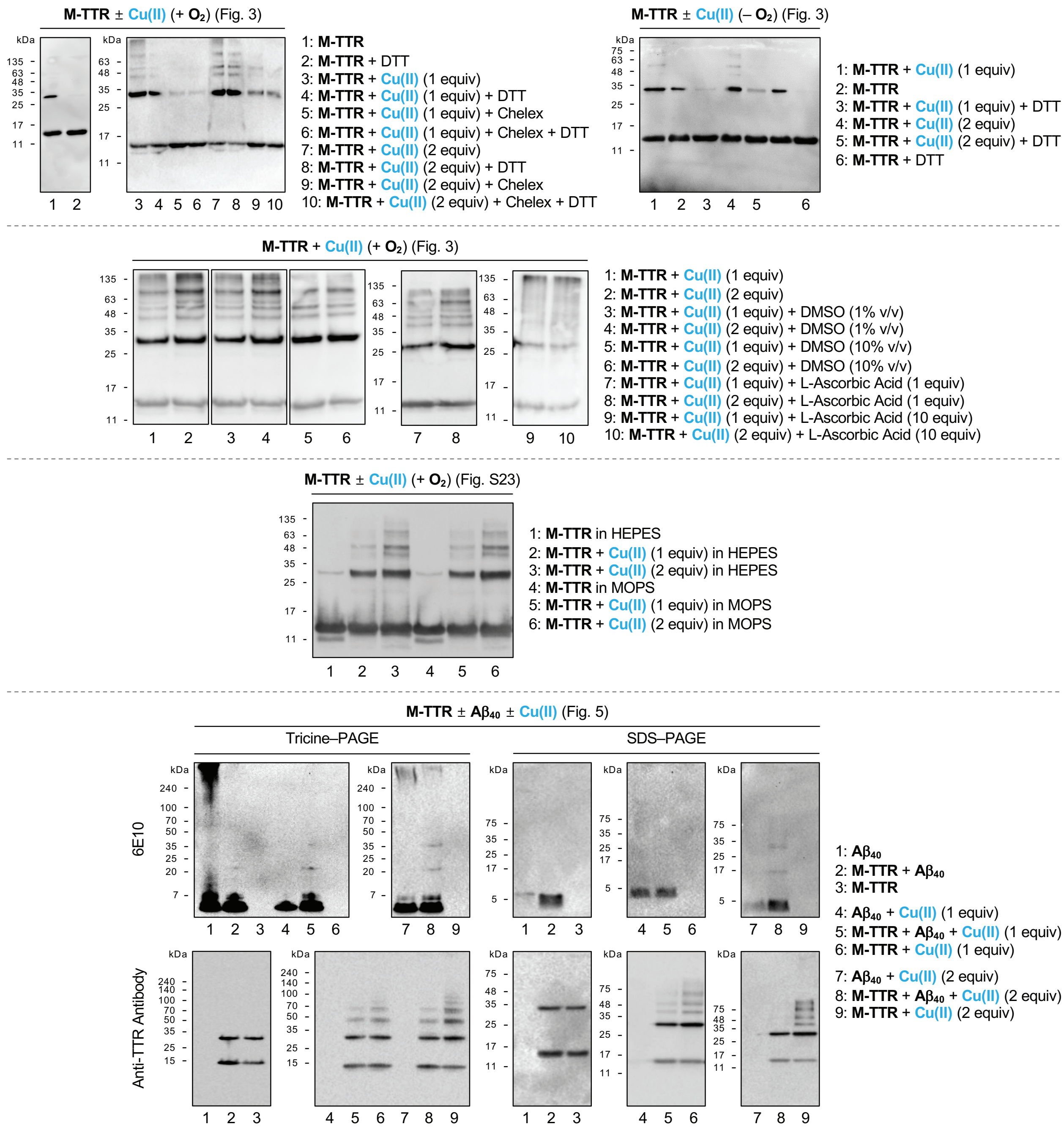

Figure S16. Unprocessed gel/Western blot data from Figures 3, S23, and 5.

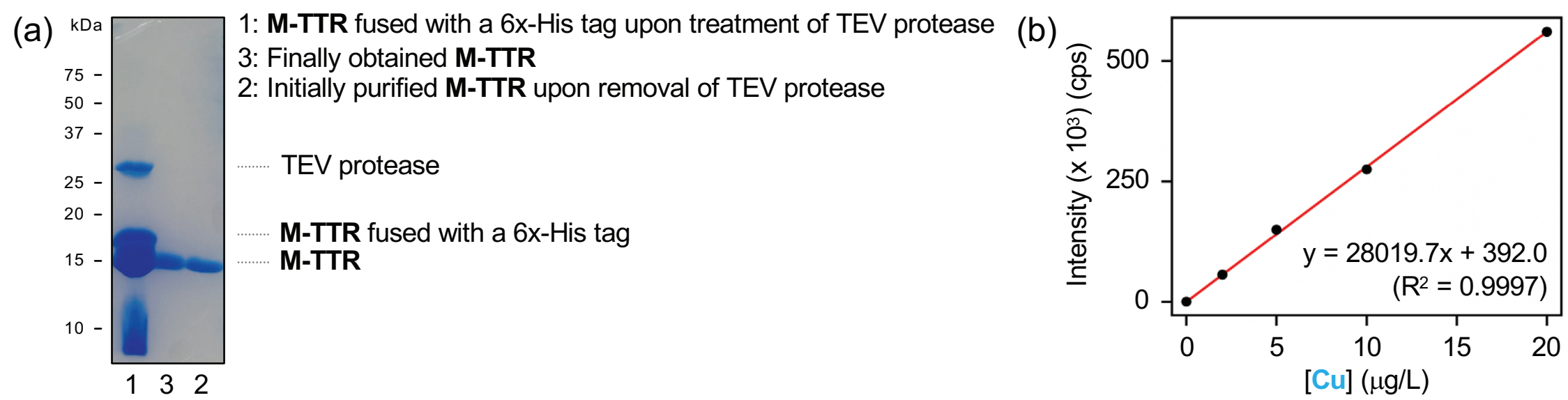

	Cu
μg/L	125.2
μM	2.7
%	0.4

Figure S17. Characterization of the sample of M-TTR. (a) Purification of the sample of M-TTR confirmed by SDS–PAGE. Conditions: [M-TTR] = 8.8 mg/mL; 20 mM HEPES, pH 7.4, 150 mM NaCl for the finally obtained sample. (b) Concentration of copper in the sample of M-TTR measured by ICP–MS. The amount of copper in the sample of M-TTR was analyzed following the standard curve obtained using various concentrations of copper (2, 5, 10, and 20 μg/L). Conditions: [M-TTR] = 74 μM; ddH_2O.

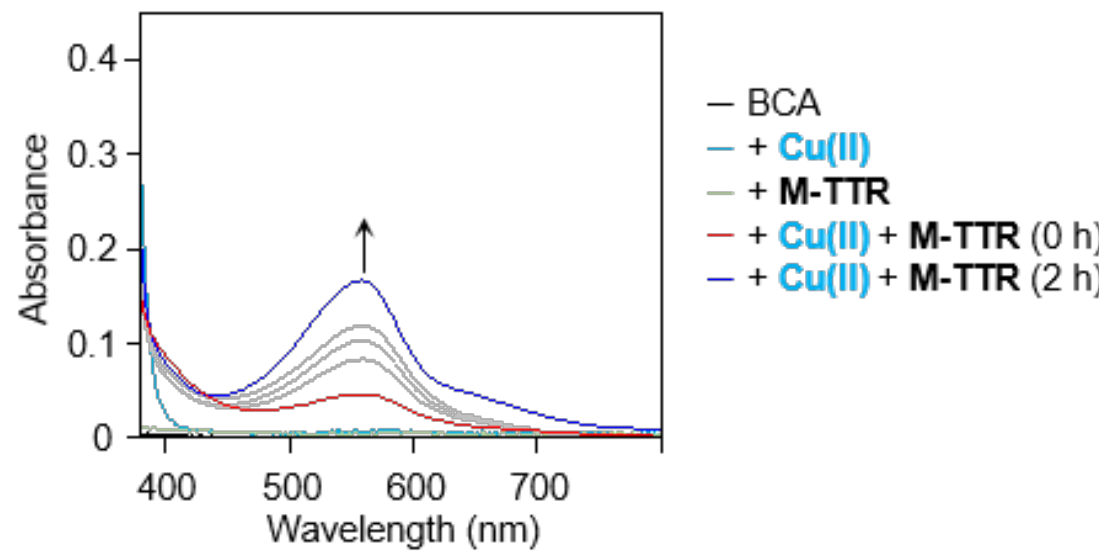

Figure S18. Abs spectra of BCA-containing samples incubated with M-TTR and Cu(II) for 2 h. Conditions: [M-TTR] = 25 μM; [$CuCl_2$] = 25 μM; [BCA] = 100 μM; 20 mM HEPES, pH 7.4, 150 mM NaCl; room temperature.

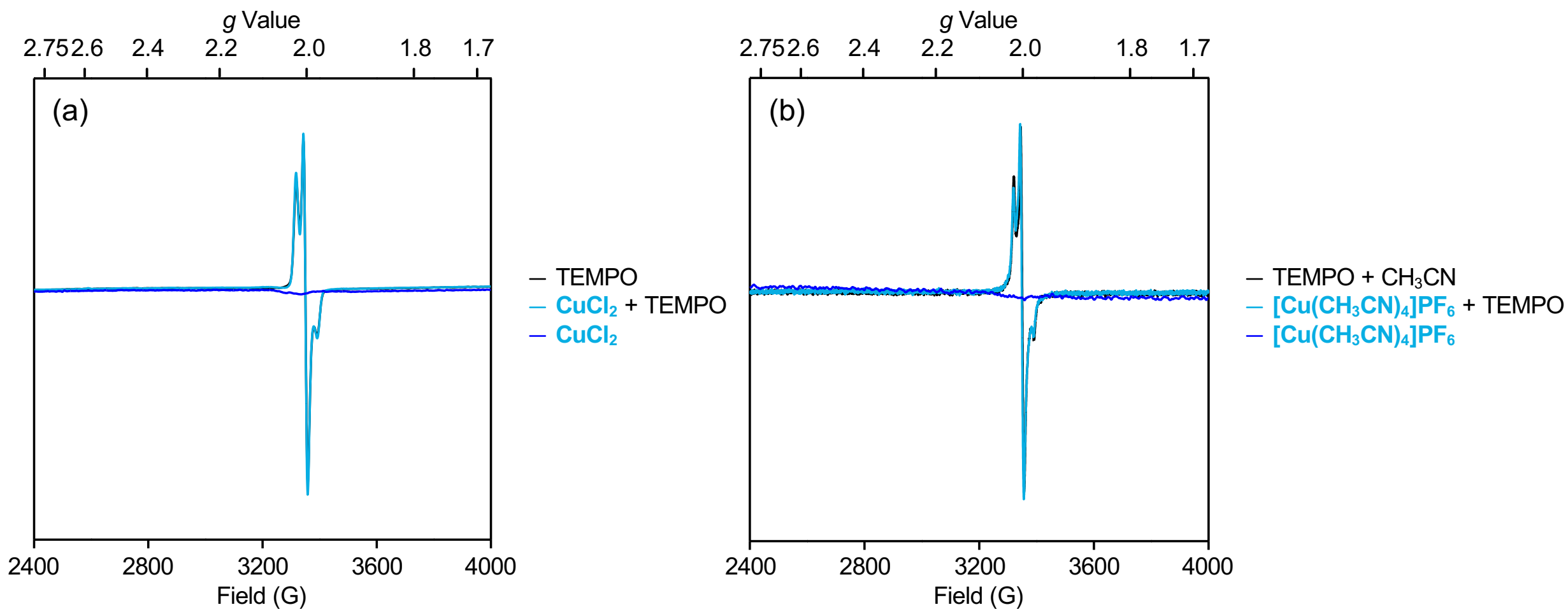

Figure S19. EPR spectra of Cu(I/II), TEMPO, and their mixtures without O_2. Conditions: [TEMPO] = 100 μM (with or without 0.2% v/v CH_3CN); [$CuCl_2$] = 100 μM; [[$Cu(CH_3CN)_4$]PF_6] = 100 μM; 20 mM HEPES, pH 7.4, 150 mM NaCl.

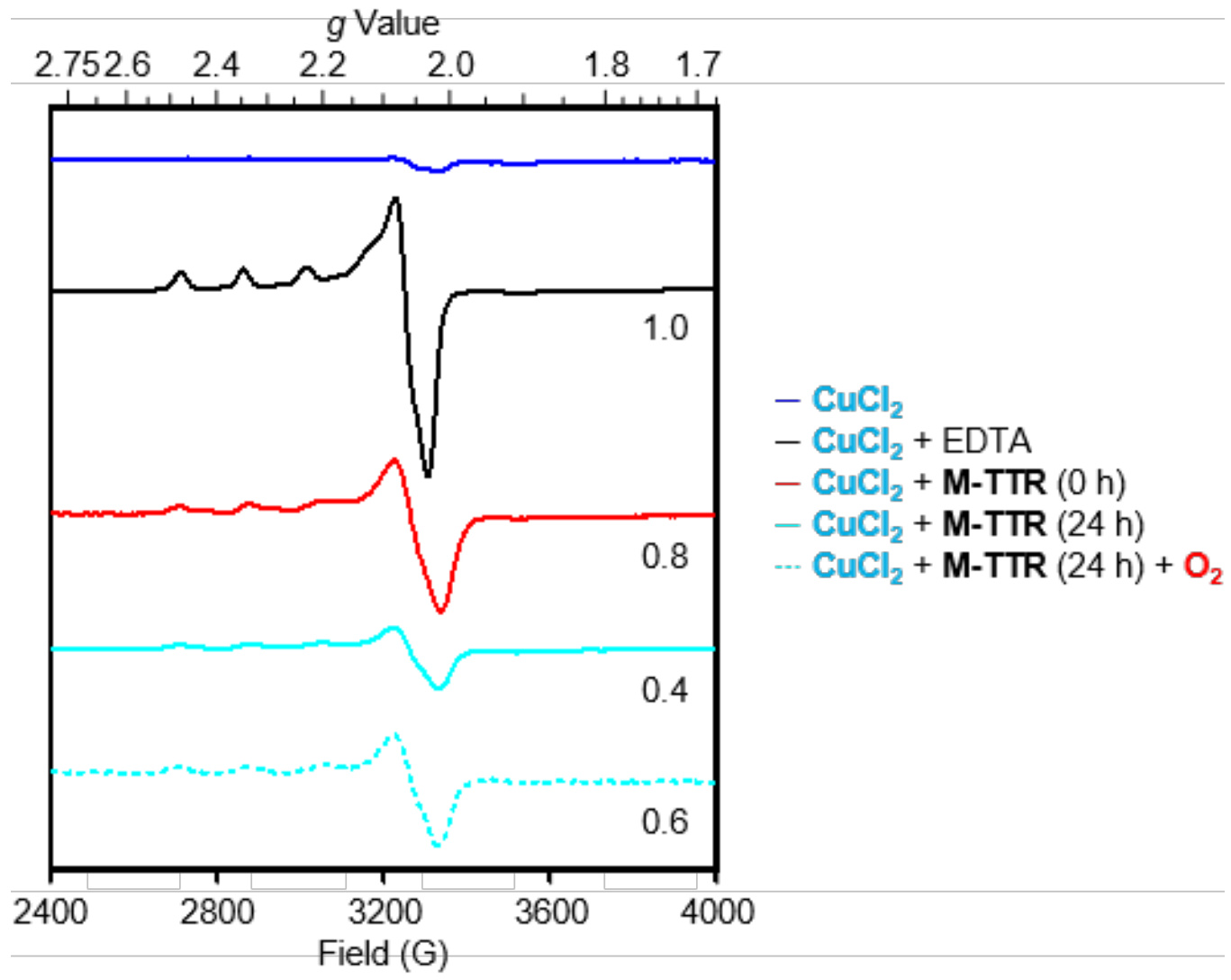

Figure S20. EPR spectra of $CuCl_2$, M-TTR, and their mixtures under anaerobic conditions. The spectrum of M-TTR with $CuCl_2$ incubated without O_2 for 24 h (cyan, solid line) was obtained, followed by exposure to O_2 for 20 min (cyan, dashed line). The numbers below each spectrum indicate the spin number relative to that of Cu(II)(EDTA) complex. Conditions: [M-TTR] = 100 μM; [$CuCl_2$] = 100 μM; 20 mM HEPES, pH 7.4, 150 mM NaCl.

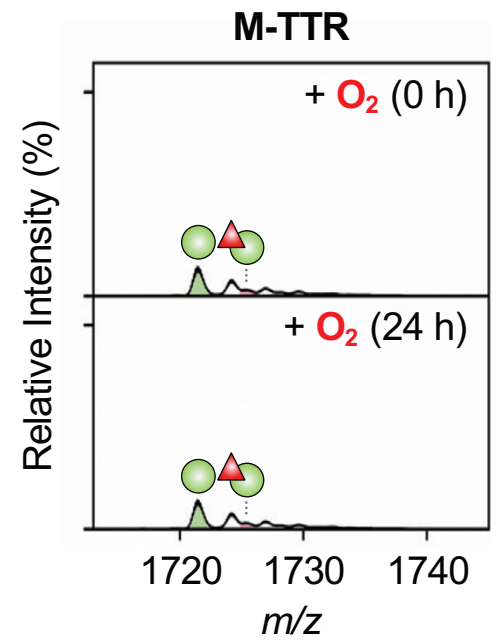

Figure S21. MS spectra of M-TTR incubated without Cu(II) under aerobic conditions. The peaks of native M-TTR are highlighted in green. Conditions: [M-TTR] = 100 μM; 20 mM ammonium acetate, pH 7.4; 37 °C; 24 h incubation; quiescent conditions. The samples were diluted 10-fold before injection into the mass spectrometer. The measurements were conducted in triplicate.

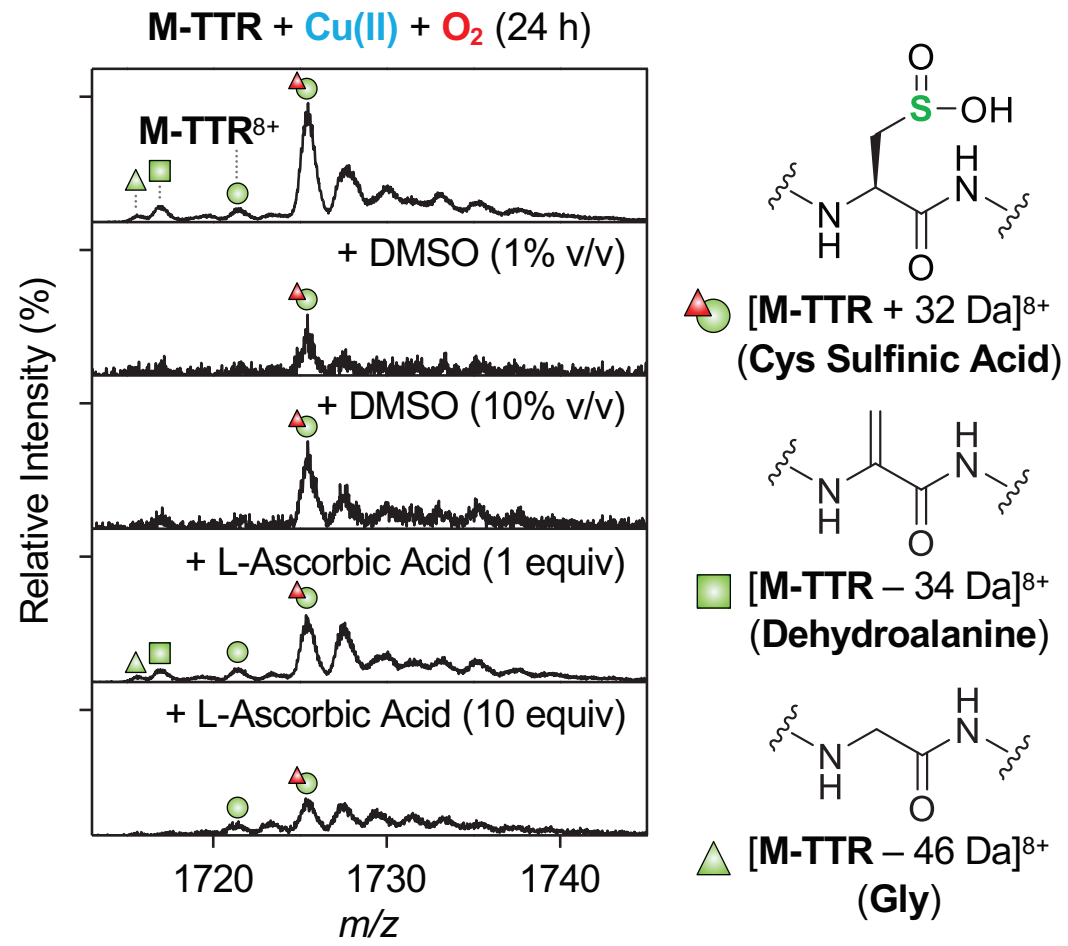

Figure S22. MS spectra of Cu(II)-treated M-TTR incubated with either DMSO or ascorbic acid under aerobic conditions. The peaks of native M-TTR are highlighted in green. Conditions: [M-TTR] = 100 μM; [$CuCl_2$] = 100 μM; 1% or 10% v/v DMSO; [L-ascorbic acid] = 100 μM and 1000 μM; 20 mM ammonium acetate, pH 7.4; 37 °C; 24 h incubation; quiescent conditions. The samples were diluted 10-fold before injection into the mass spectrometer. The measurements were conducted in triplicate.

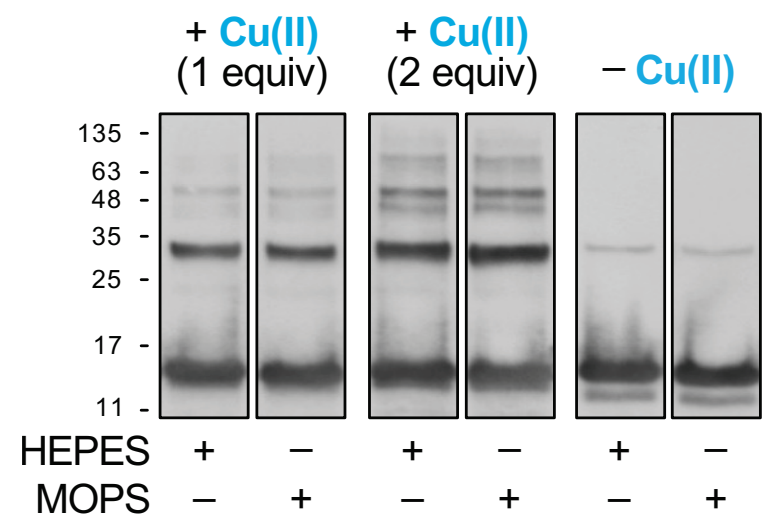

Figure S23. Size distribution of the M-TTR incubated with or without Cu(II) in MOPS buffer probed by SDS–PAGE with Western blotting using an anti-TTR antibody. Conditions: [M-TTR] = 25 μM; [$CuCl_2$] = 25 μM and 50 μM; 20 mM HEPES or MOPS, pH 7.4, 150 mM NaCl; 37 °C; 24 h incubation; quiescent conditions.

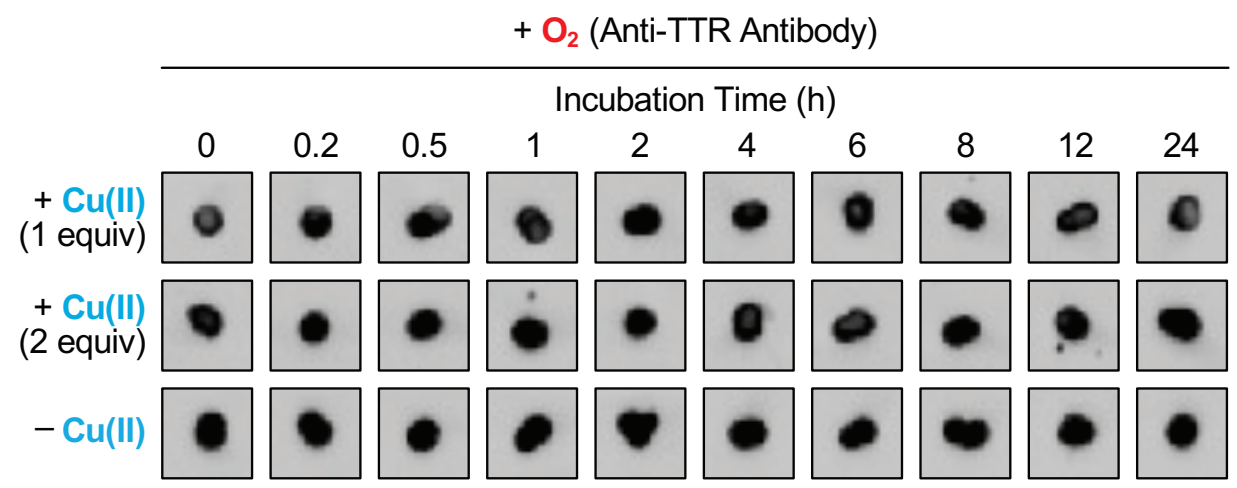

Figure S24. Dot blots of Cu(II)-treated and untreated protein samples using an anti-TTR antibody. The measurements were conducted in triplicate. Conditions: [M-TTR] = 25 μM; [$CuCl_2$] = 25 μM and 50 μM; 20 mM HEPES, pH 7.4, 150 mM NaCl; 37 °C; 24 h incubation; quiescent conditions.

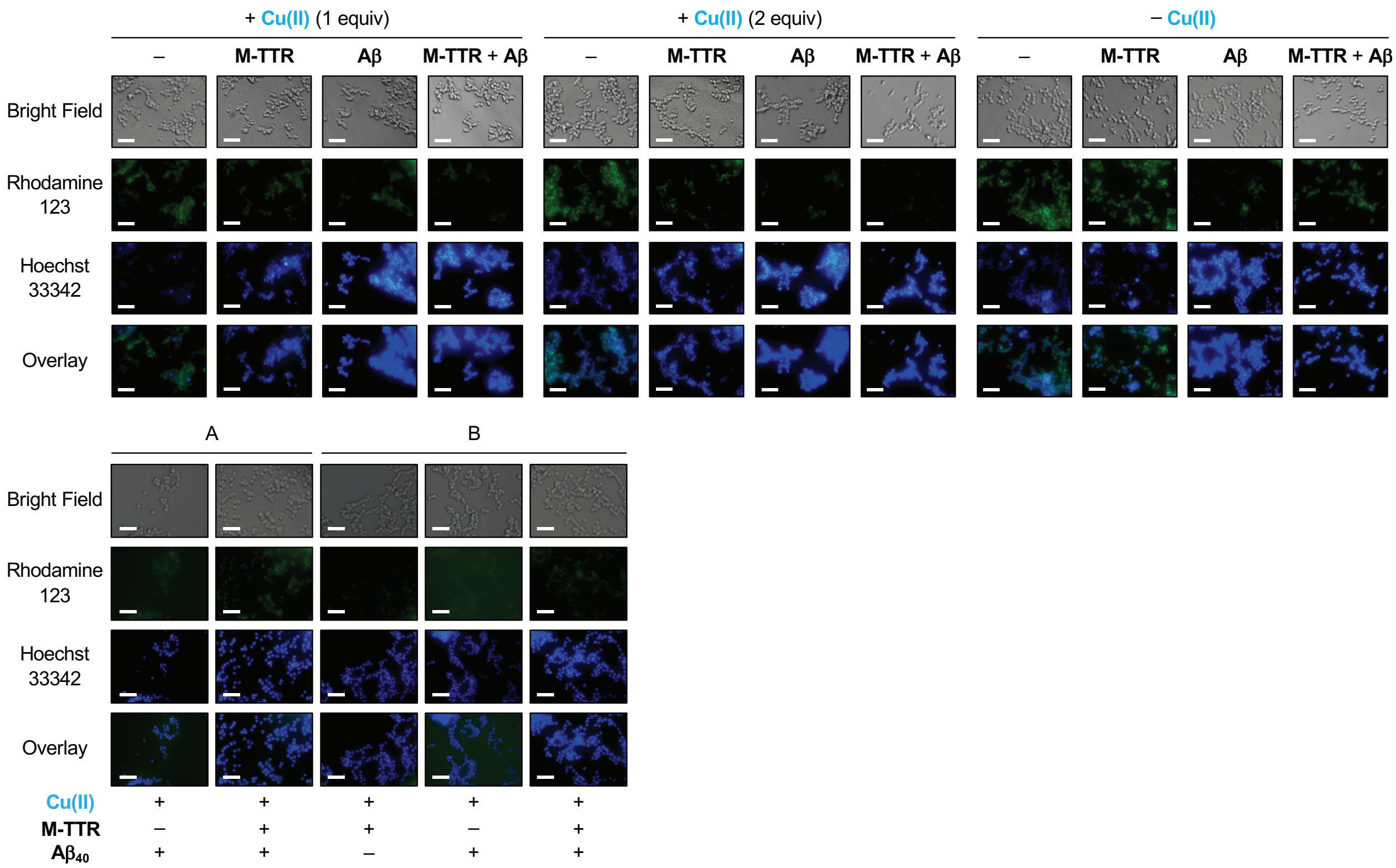

Figure S25. Apoptosis of the cells treated with or without M-TTR, Cu(II), $A\beta_{40}$, or their mixtures. Conditions: [M-TTR] = 25 μM; [$A\beta_{40}$] = 25 μM; [$CuCl_2$] = 25 μM and 50 μM; 20 mM HEPES, pH 7.4, 150 mM NaCl; non-differentiated 5Y cells. Scale bars, 100 μm.

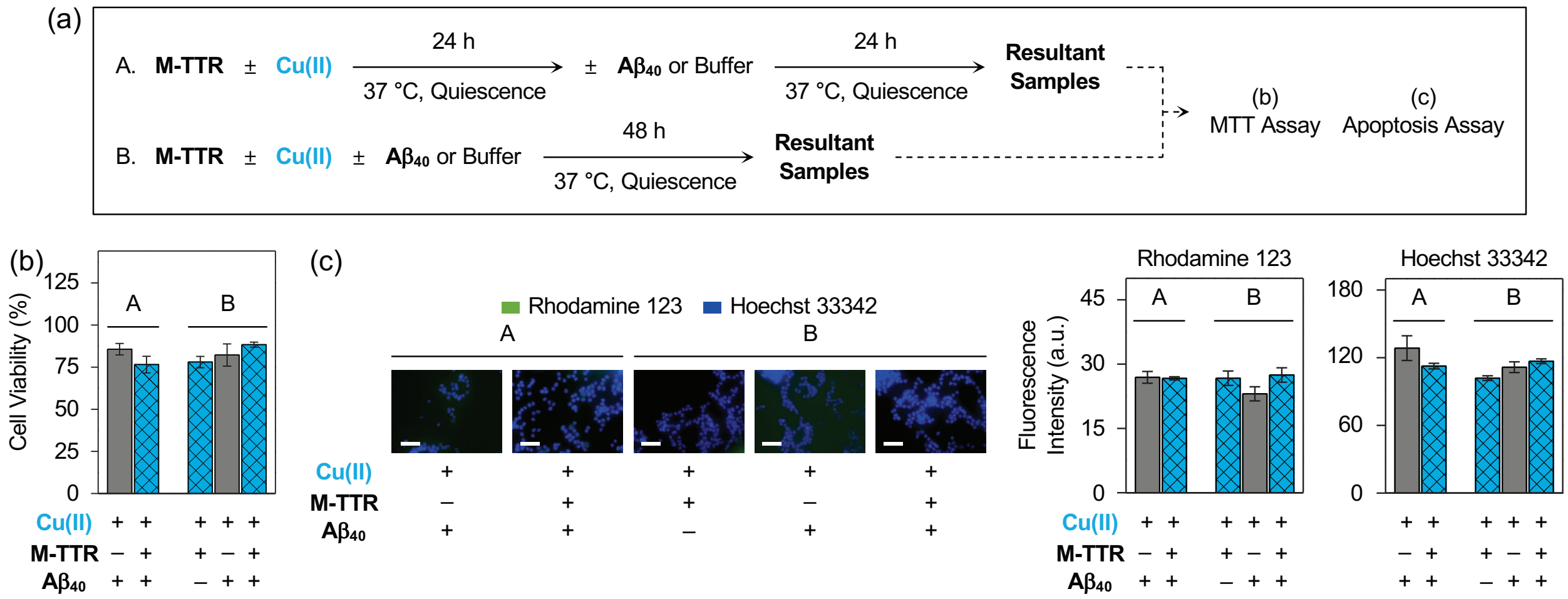

Figure S26. Cell studies for M-TTR, Cu(II), Aβ_{40}, or their mixtures. (a) Scheme of the experiments. (b) Cell survival upon incubation of the resultant samples. Cell viability, analyzed by the MTT assay, was calculated in comparison to that with an equivalent amount of the buffered solution (20 mM HEPES, pH 7.4, 150 mM NaCl). Conditions: [M-TTR] = 25 μM; [Aβ_{40}] = 25 μM; [$CuCl_2$] = 50 μM; non-differentiated 5Y cells. Error bars denote s.e.m. for n = 6 examined over three independent experiments. (c) Overlay of the rhodamine 123- and Hoechst 33342-stained cells treated with the resultant samples. The fluorescence intensity was quantified by the ImageJ software. Error bars represent s.e.m. for n = 22–138 cells. Conditions: [M-TTR] = 25 μM; [Aβ_{40}] = 25 μM; [$CuCl_2$] = 50 μM; 20 mM HEPES, pH 7.4, 150 mM NaCl; non-differentiated 5Y cells. Scale bars, 100 μm.

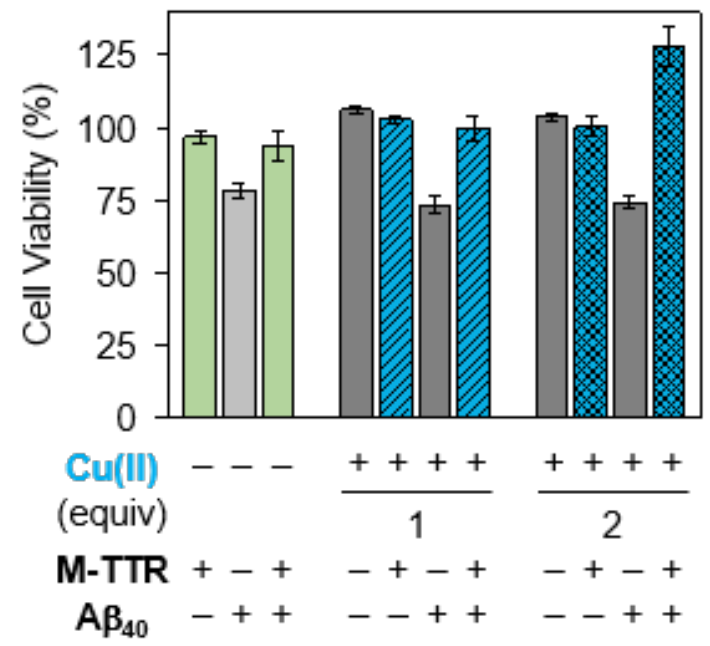

Figure S27. Survival of 5Y cells treated with M-TTR, Cu(II), $A\beta_{40}$, or their mixtures in serum-containing media. M-TTR, Cu(II), and $A\beta_{40}$, individually or in the indicated combinations, were preincubated for 24 h in the buffered solution (20 mM HEPES, pH 7.4, 150 mM NaCl). The resulting samples were subsequently added to 5Y cells cultured in media containing 10% v/v FBS. After 24 h of treatment, cell viability was determined by the MTT assay and calculated relative to that of cells treated with an equivalent volume of the buffered solution. Conditions: [M-TTR] = 25 μM; [$A\beta_{40}$] = 25 μM; [$CuCl_2$] = 25 μM and 50 μM. Error bars represent s.e.m. (*n* = 6) from three independent experiments.

References

1. Oroz, J., Kim, J. H., Chang, B. J. & Zweckstetter, M. Mechanistic basis for the recognition of a misfolded protein by the molecular chaperone Hsp90. *Nat. Struct. Mol. Biol.* **24**, 407–413 (2017).
2. Jazaj, D., Ghadami, S. A., Bemporad, F., & Chiti, F. Probing conformational changes of monomeric transthyretin with second derivative fluorescence. *Sci. Rep.* **9**, 10988 (2019).
3. Thordarson, P. Determining association constants from titration experiments in supramolecular chemistry. *Chem. Soc. Rev.* **40**, 1305–1323 (2011).
4. Harris, D. C. Quantitative chemical analysis. W. H. Freeman and Company (2010).
5. Yatsunyk, L. A. & Rosenzweig, A. C. Cu(I) Binding and Transfer by the N Terminus of the Wilson Disease Protein. *J. Biol. Chem.* **282**, 8622–8631 (2007).
6. Neese, F. The program EPR. *QCPE Bull.* **15**, 5 (1995).
7. Lieberman, R. L., Arciero, D. M., Hooper, A. B. & Rosenzweig, A. C. Crystal structure of a novel red copper protein from *Nitrosomonas europaea*. *Biochemistry* **40**, 5674–5681 (2001).
8. Palmieri, L. d. C. et al. Novel Zn^{2+}-binding sites in human transthyretin—Implications for amyloidogenesis and retinol-binding protein recognition. *J. Biol. Chem.* **285**, 31731–31741 (2010).
9. Runge, E. & Gross, E. K. U. Density-functional theory for time-dependent systems. *Phys. Rev. Lett.* **52**, 997–1000 (1984).
10. Frisch, M. J. et al. Gaussian 09. *Revision A* **2** (2016).
11. Zhao, Y. & Truhlar, D. G. The M06 suite of density functionals for main group thermochemistry, thermochemical kinetics, noncovalent interactions, excited states, and transition elements: two new functionals and systematic testing of four M06-class functionals and 12 other functionals. *Theor. Chem. Acc.* **120**, 215–241 (2008).
12. Becke, A. D. Density-functional exchange-energy approximation with correct asymptotic behavior. *Phys. Rev. A* **38**, 3098–3100 (1988).
13. Perdew, J. P. Density-functional approximation for the correlation energy of the inhomogeneous electron gas. *Phys. Rev. B* **33**, 8822–8824 (1986).
14. Vosko, S. H., Wilk, L. & Nusair, M. Accurate spin-dependent electron liquid correlation energies for local spin density calculations: a critical analysis. *Can. J. Phys.* **58**, 1200–1211 (1980).
15. Lee, C., Yang, W. & Parr, R. G. Development of the Colle-Salvetti correlation-energy formula into a functional of the electron density. *Phys. Rev. B* **37**, 785–789 (1988).
16. Ditchfield, R., Hehre, W. J. & Pople, J. A. Self-consistent molecular-orbital methods. IX. An

extended Gaussian-type basis for molecular-orbital studies of organic molecules. *J. Chem. Phys.* **54**, 724–728 (1971).

17. Francl, M. M. et al. Self-consistent molecular orbital methods. XXIII. A polarization-type basis set for second-row elements. *J. Chem. Phys.* **77**, 3654–3665 (1982).
18. Hariharan, P. C. & Pople, J. A. The influence of polarization functions on molecular orbital hydrogenation energies. *Theor. Chim. Acta* **28**, 213–222 (1973).
19. Hehre, W. J., Ditchfield, R. & Pople, J. A. Self—consistent molecular orbital methods. XII. Further extensions of Gaussian—type basis sets for use in molecular orbital studies of organic molecules. *J. Chem. Phys.* **56**, 2257–2261 (1972).
20. Rassolov, V. A., Pople, J. A., Ratner, M. A. & Windus, T. L. 6-31G* basis set for atoms K through Zn. *J. Chem. Phys.* **109**, 1223–1229 (1998).
21. Barone, V. & Cossi, M. Quantum calculation of molecular energies and energy gradients in solution by a conductor solvent model. *J. Phys. Chem. A* **102**, 1995–2001 (1998).
22. Camaioni, D. M. & Schwerdtfeger, C. A. Comment on "accurate experimental values for the free energies of hydration of H^+, $OH^–$, and H_3O^+". *J. Phys. Chem. A* **109**, 10795–10797 (2005).
23. Neese F. The ORCA program system. *WIREs Comput. Mol. Sci.* **2**, 73–78 (2012).
24. Neese F. Software update: the ORCA program system, version 4.0. *WIREs Comput. Mol. Sci.* **8**, e1327 (2018).
25. Neese F. Prediction and interpretation of the ^{57}Fe isomer shift in Mössbauer spectra by density functional theory. *Inorg. Chim. Acta* **337**, 181–192 (2002).
26. Rega, N., Cossi, M. & Barone, V. Development and validation of reliable quantum mechanical approaches for the study of free radicals in solution. *J. Chem. Phys.* **105**, 11060–11067 (1996).
27. Mruk, D. D. & Cheng, C. Y. Enhanced chemiluminescence (ECL) for routine immunoblotting: an inexpensive alternative to commercially available kits. *Spermatogenesis* **1**, 121–122 (2011).
28. Yi, Y. et al. Impact of sphingosine and acetylsphingosines on the aggregation and toxicity of metal-free and metal-treated amyloid-β. *Chem. Sci.* **12**, 2456–2466 (2021).
29. Thomson, A. C. et al. The effects of serum removal on gene expression and morphological plasticity markers in differentiated SH-SY5Y cells. *Cell. Mol. Neurobiol.* **42**, 1829–1839 (2022).
30. Reixach, N., Deechongkit, S., Jiang, X., Kelly, J. W. & Buxbaum, J. N. Tissue damage in the amyloidoses: transthyretin monomers and nonnative oligomers are the major cytotoxic

species in tissue culture. *Proc. Natl. Acad. Sci. USA* **101**, 2817–2822 (2004).

31. Ramkumar, M. et al. Neuroprotective effect of Demethoxycurcumin, a natural derivative of Curcumin on rotenone induced neurotoxicity in SH-SY 5Y neuroblastoma cells. *BMC Complement. Altern. Med.* **17**, 217 (2017).
32. Luo, Z., Zhao, Y., Wang, Y., Yang, X. & Zhao, B. Protective effect of theaflavins on neuron against 6-hydroxydopamine-induced apoptosis in SH-SY5Y cells. *J. Clin. Biochem. Nutr.* **50**, 133–138 (2012).
33. Feng, C. et al. Lycopene protects human SH-SY5Y neuroblastoma cells against hydrogen peroxide-induced death via inhibition of oxidative stress and mitochondria-associated apoptotic pathways. *Mol. Med. Rep.* **13**, 4205–4214 (2016).
34. Gao, K. et al. Lyciumamide A, a dimer of phenolic amide, protects against NMDA-induced neurotoxicity and potential mechanisms in vitro. *J. Mol. Histol.* **52**, 449–459 (2021).
35. Ainscough, E. W. et al. Spectrochemical studies on the blue copper protein azurin from *Alcaligenes denitrificans*. *Biochemistry* **26**, 71–82 (1987).
36. Penfield, K. W. et al. Spectroscopic studies on plastocyanin single crystals: a detailed electronic structure determination of the blue copper active site. *J. Am. Chem. Soc.* **103**, 4382–4388 (1981).
37. LaCroix, L. B. et al. Spectroscopic and geometric variations in perturbed blue copper centers: electronic structures of stellacyanin and cucumber basic protein. *J. Am. Chem. Soc.* **120**, 9621–9631 (1998).
38. Solomon, E. I. Spectroscopic methods in bioinorganic chemistry: blue to green to red copper sites. *Inorg. Chem.* **45**, 8012–8025 (2006).
39. Zhekova, H. R., Seth, M. & Ziegler, T. A magnetic and electronic circular dichroism study of azurin, plastocyanin, cucumber basic protein, and nitrite reductase based on time-dependent density functional theory calculations. *J. Phys. Chem. A* **114**, 6308–6321 (2010).
40. Pantoliano, M. W., Valentine, J. S. & Nafie, L. A. Spectroscopic studies of copper(II) bound at the native copper site or substituted at the native zinc site of bovine erythrocuprein (superoxide dismutase). *J. Am. Chem. Soc.* **104**, 6310–6317 (1982).
41. Knowles, P. F. et al. Spectroscopic studies of the active site of galactose oxidase. *Inorg. Chem.* **34**, 3895–3902 (1995).
42. Kooter, I. M. et al. EPR characterization of the mononuclear Cu-containing *Aspergillus japonicus* quercetin 2,3-dioxygenase reveals dramatic changes upon anaerobic binding of substrates. *Eur. J. Biochem.* **269**, 2971–2979 (2002).
43. Basumallick, L. et al. Spectroscopic and density functional studies of the red copper site in

nitrosocyanin: role of the protein in determining active site geometric and electronic structure. *J. Am. Chem. Soc.* **127**, 3531–3544 (2005).
44. Girotto, S. et al. DJ-1 is a copper chaperone acting on SOD1 activation. *J. Biol. Chem.* **289**, 10887–10899 (2014).
45. Hughey IV, J. L. et al. Preparation and characterization of [*rac*-5,7,7,12,14,14,-hexamethyl-1,4,8,11-tetraazocyclotetradecane]copper(II) *o*-mercaptobenzoate hydrate, [Cu(tet b)(*o*-$SC_6H_4CO_2$)]. H_2O, a complex with a CuN_4S (mercaptide) chromophore. *J. Am. Chem. Soc.* **101**, 2617–2623 (1979).
46. Miskowski, V. M., Thich, J. A., Solomon, R. & Schugar, H. J. Electronic spectra of substituted copper(II) thioether complexes. *J. Am. Chem. Soc.* **98**, 8344–8350 (1976).
47. Mandal, S. & Bharadwaj, P. K. Synthesis and characterisation of copper(II) complexes having $N_2S^{\dagger}S$ and $N_2S^{\dagger}O$ ($S^{\dagger}$ = thioether) donor sets. *Indian J. Chem.* **30A**, 948–951 (1991).
48. Rohatgi, A. WebPlotDigitizer version 4.6, https://automeris.io/WebPlotDigitizer (2022).
